# Supplementary figures and images for: UBXN1 maintains ER proteostasis and represses UPR activation by modulating translation
Source: EMBO Rep. 2024 Jan 2;25(2):15. doi: 10.1038/s44319-023-00027-z (PMC10897191; doi:10.1038/s44319-023-00027-z)

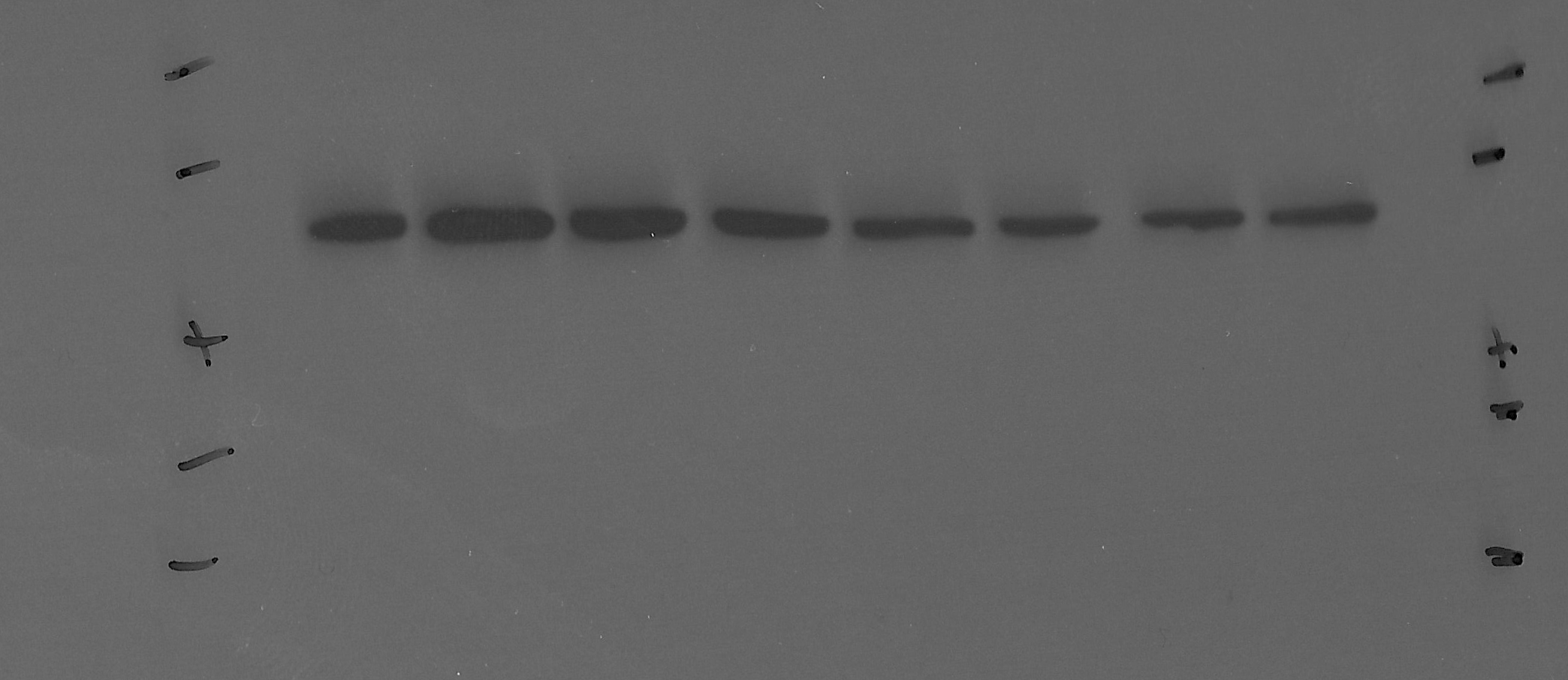

Supplement: Supplementary file 4 — Source Data Fig. 1 [file 44319_2023_27_MOESM4_ESM.zip › Figure 1/1A/western PCNA 1.tif]

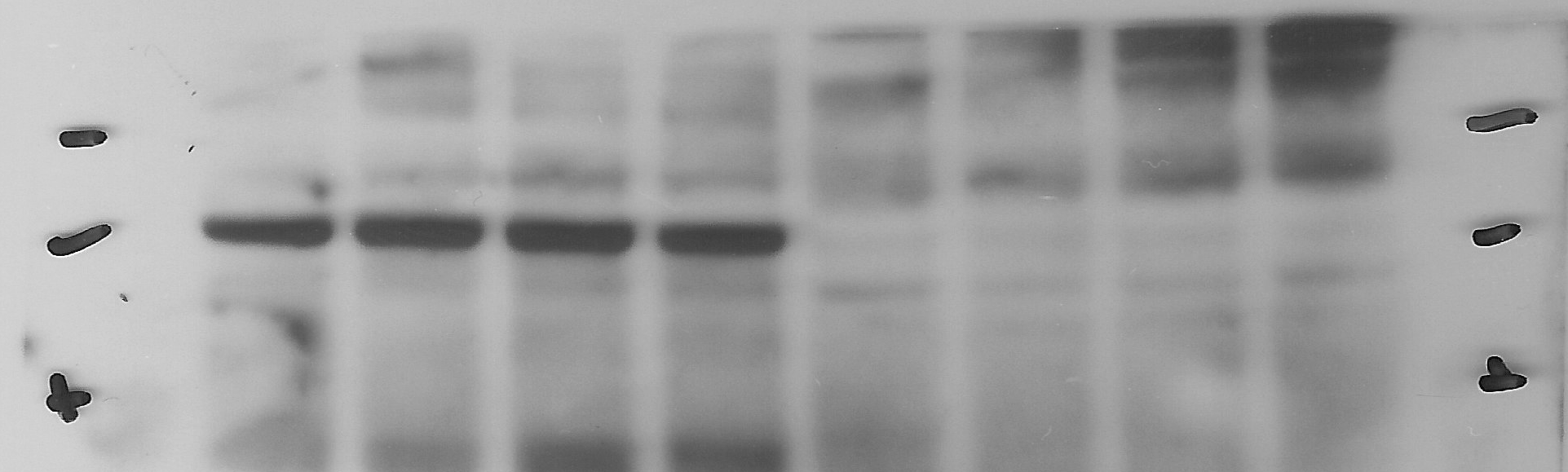

Supplement: Supplementary file 4 — Source Data Fig. 1 [file 44319_2023_27_MOESM4_ESM.zip › Figure 1/1A/western UBXN1.tif]

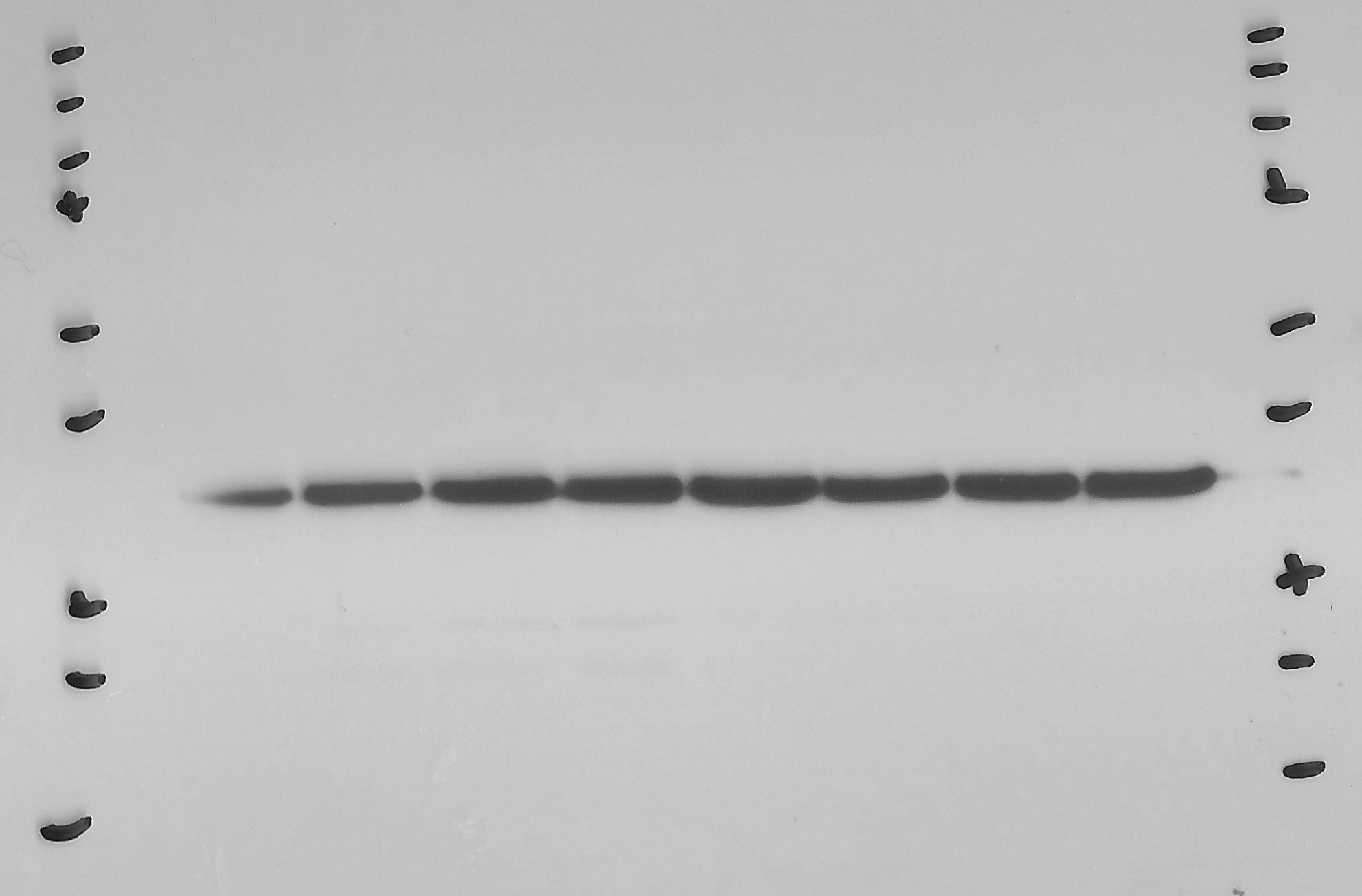

Supplement: Supplementary file 4 — Source Data Fig. 1 [file 44319_2023_27_MOESM4_ESM.zip › Figure 1/1A/western PCNA 2.tif]

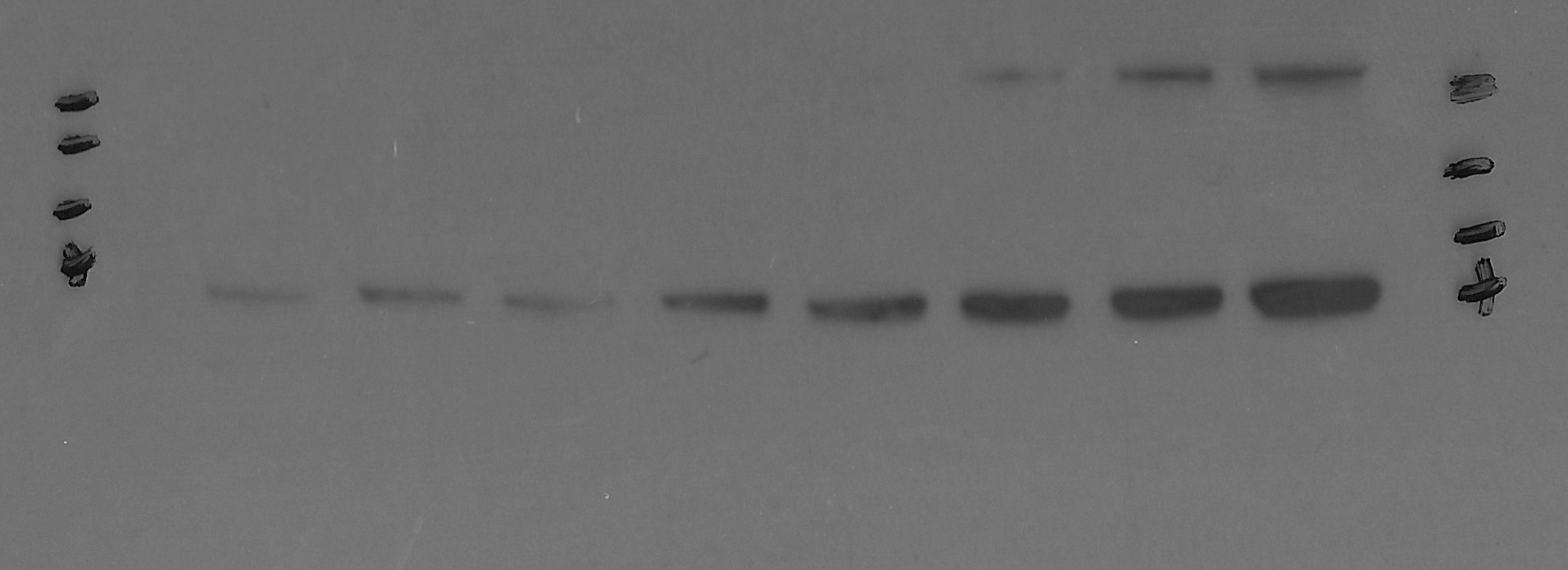

Supplement: Supplementary file 4 — Source Data Fig. 1 [file 44319_2023_27_MOESM4_ESM.zip › Figure 1/1A/western BiP.tif]

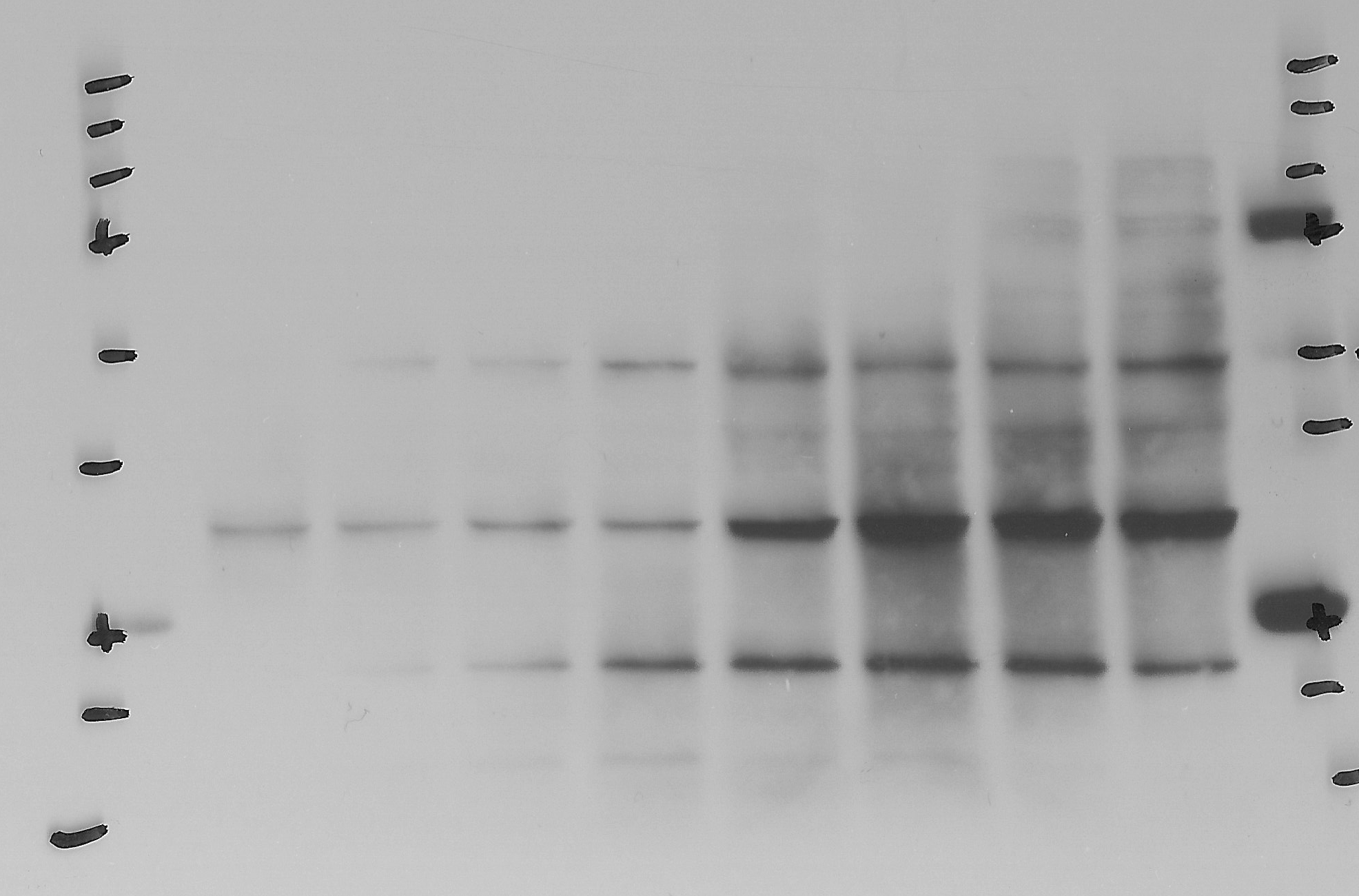

Supplement: Supplementary file 4 — Source Data Fig. 1 [file 44319_2023_27_MOESM4_ESM.zip › Figure 1/1A/western ATF4.tif]

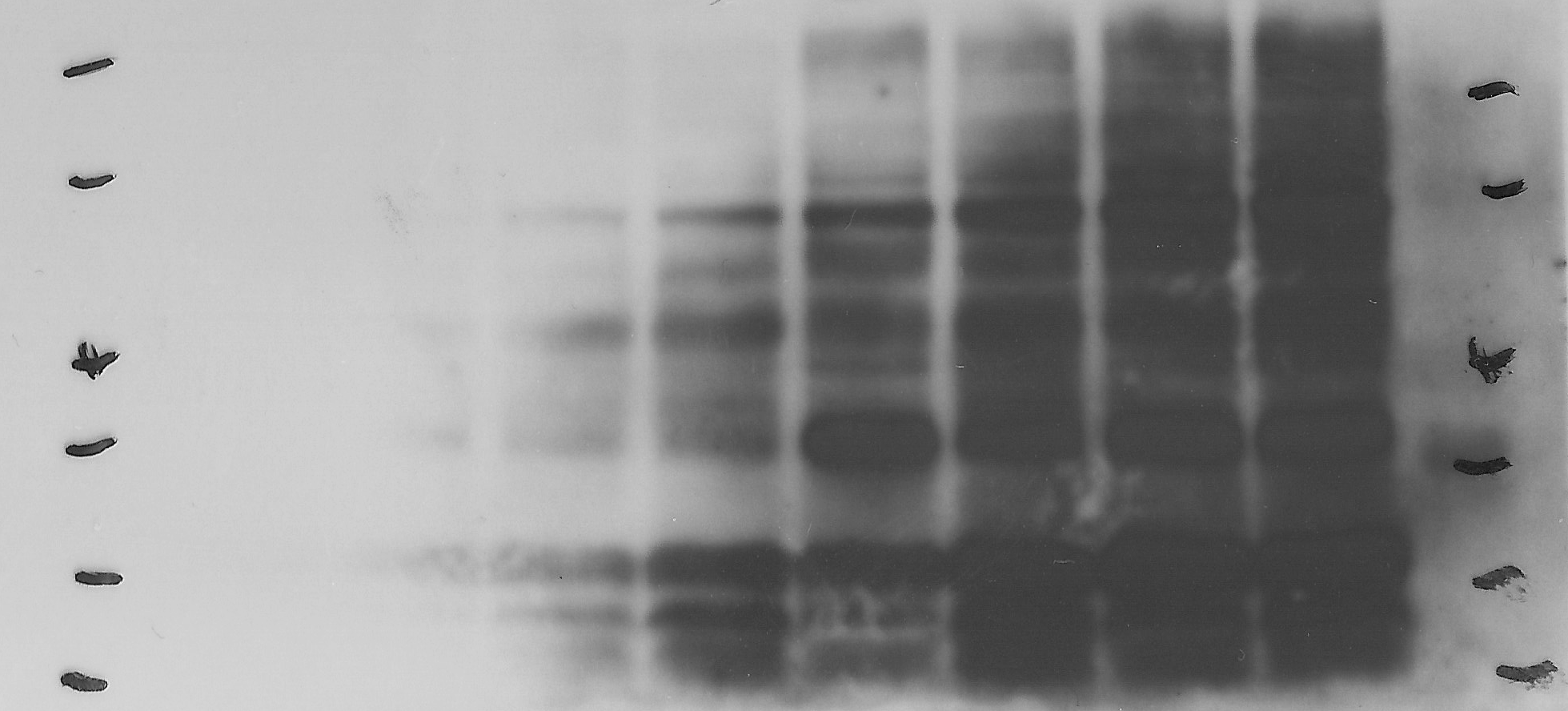

Supplement: Supplementary file 4 — Source Data Fig. 1 [file 44319_2023_27_MOESM4_ESM.zip › Figure 1/1A/western peIF2a.tif]

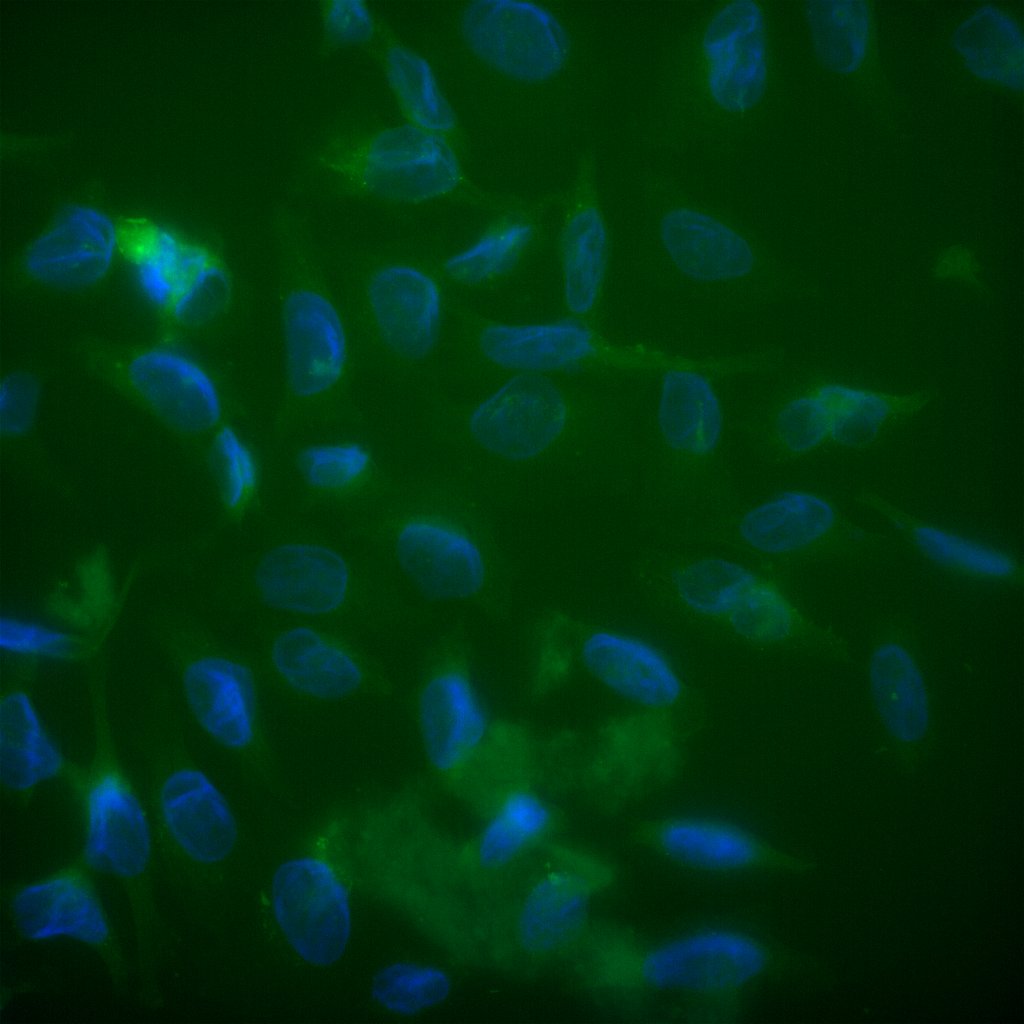

Supplement: Supplementary file 4 — Source Data Fig. 1 [file 44319_2023_27_MOESM4_ESM.zip › Figure 1/1E/Micr Image_siCTRL_Tu.tif]

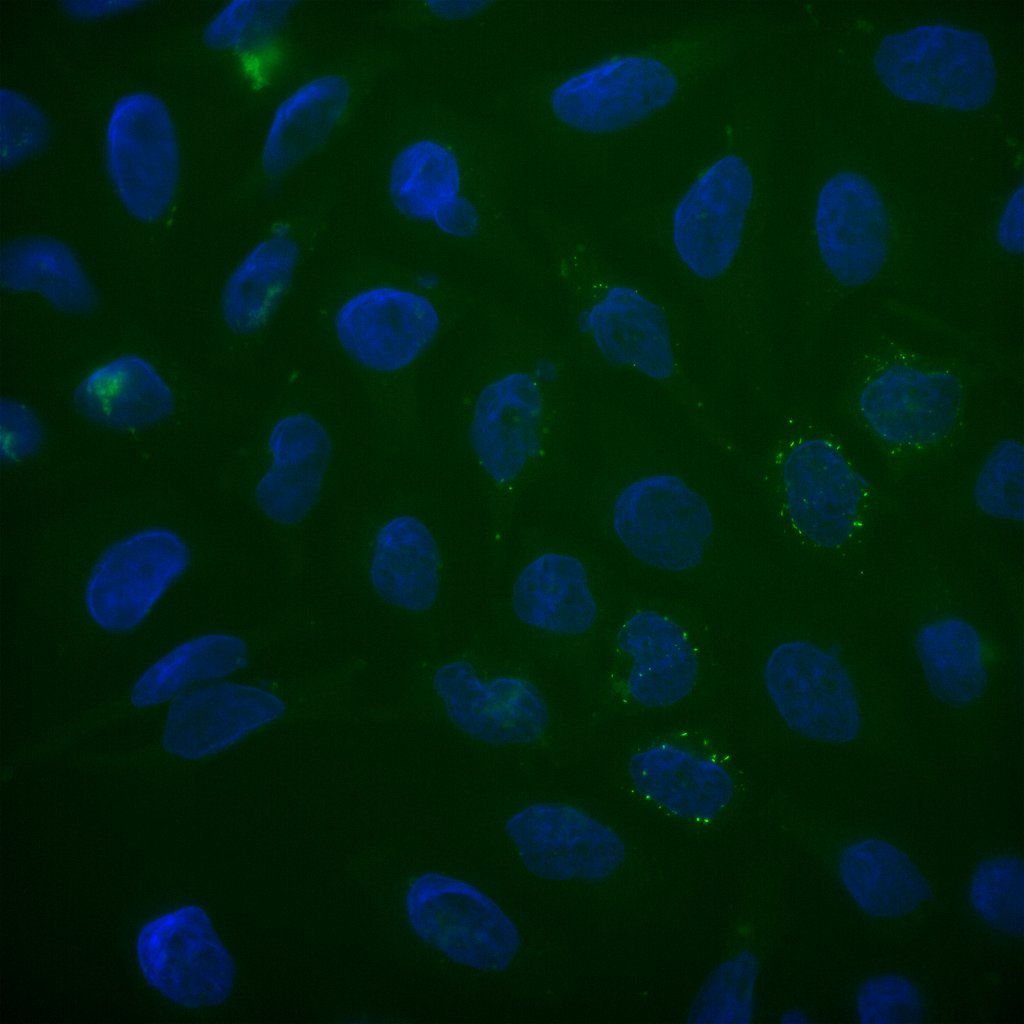

Supplement: Supplementary file 4 — Source Data Fig. 1 [file 44319_2023_27_MOESM4_ESM.zip › Figure 1/1E/Micr Image_siUBXN1_2_Tu_.tif]

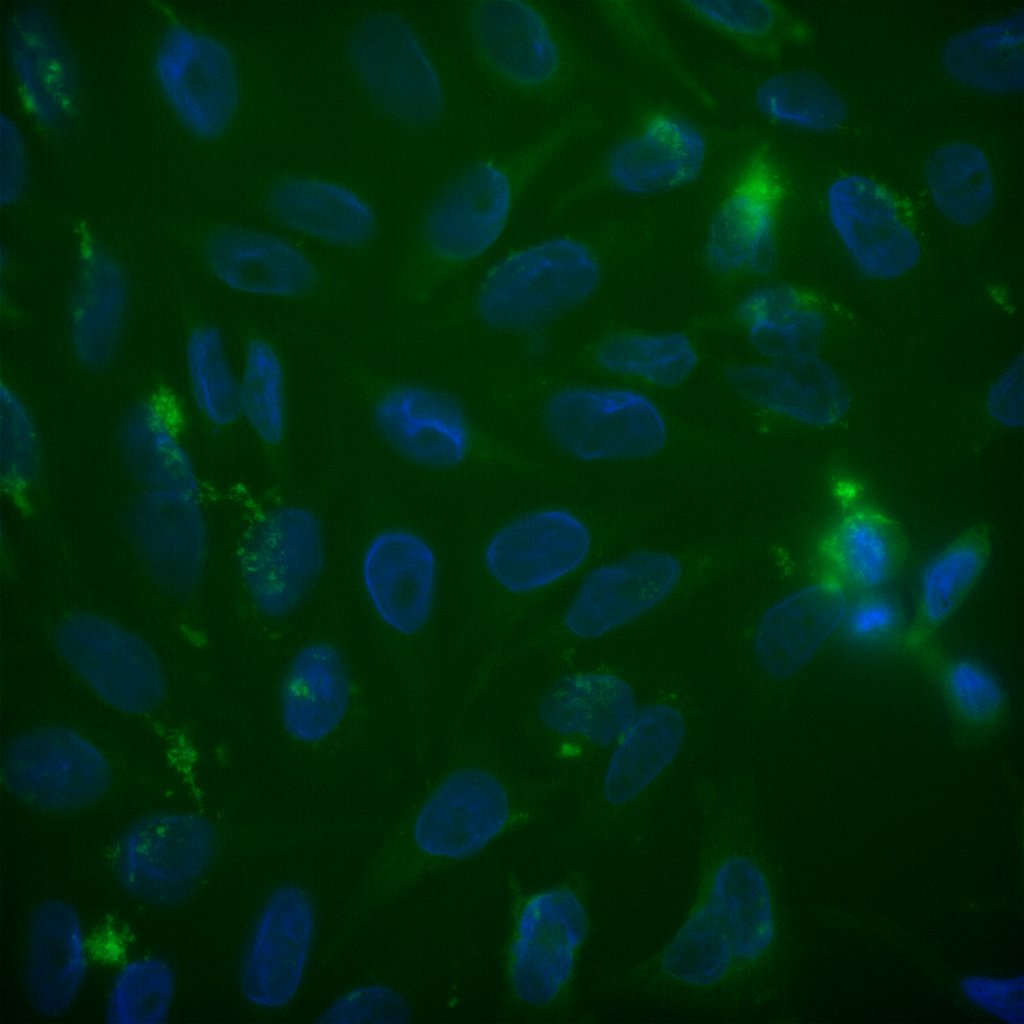

Supplement: Supplementary file 4 — Source Data Fig. 1 [file 44319_2023_27_MOESM4_ESM.zip › Figure 1/1E/Micr Image siUBXN1_2_untreated_.tif]

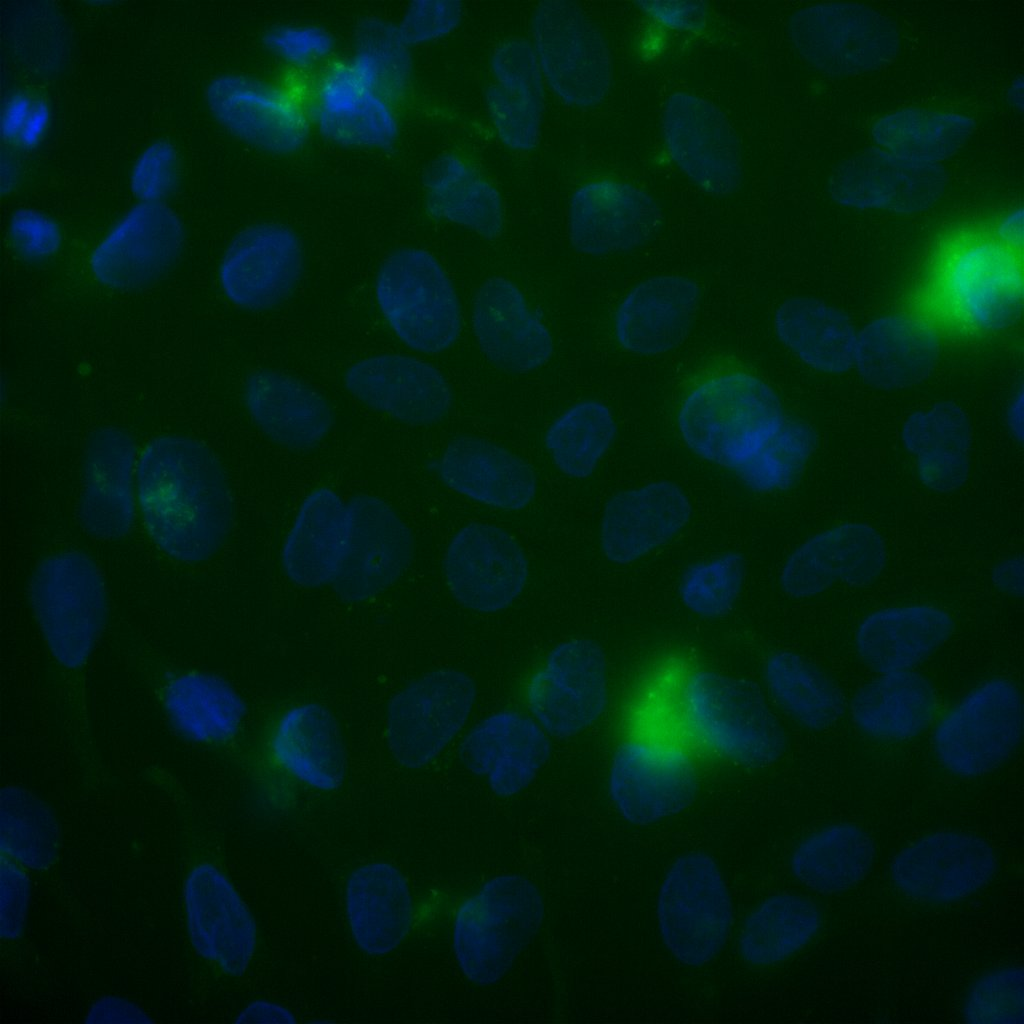

Supplement: Supplementary file 4 — Source Data Fig. 1 [file 44319_2023_27_MOESM4_ESM.zip › Figure 1/1E/Micr Image_siCTRL untreated_.tif]

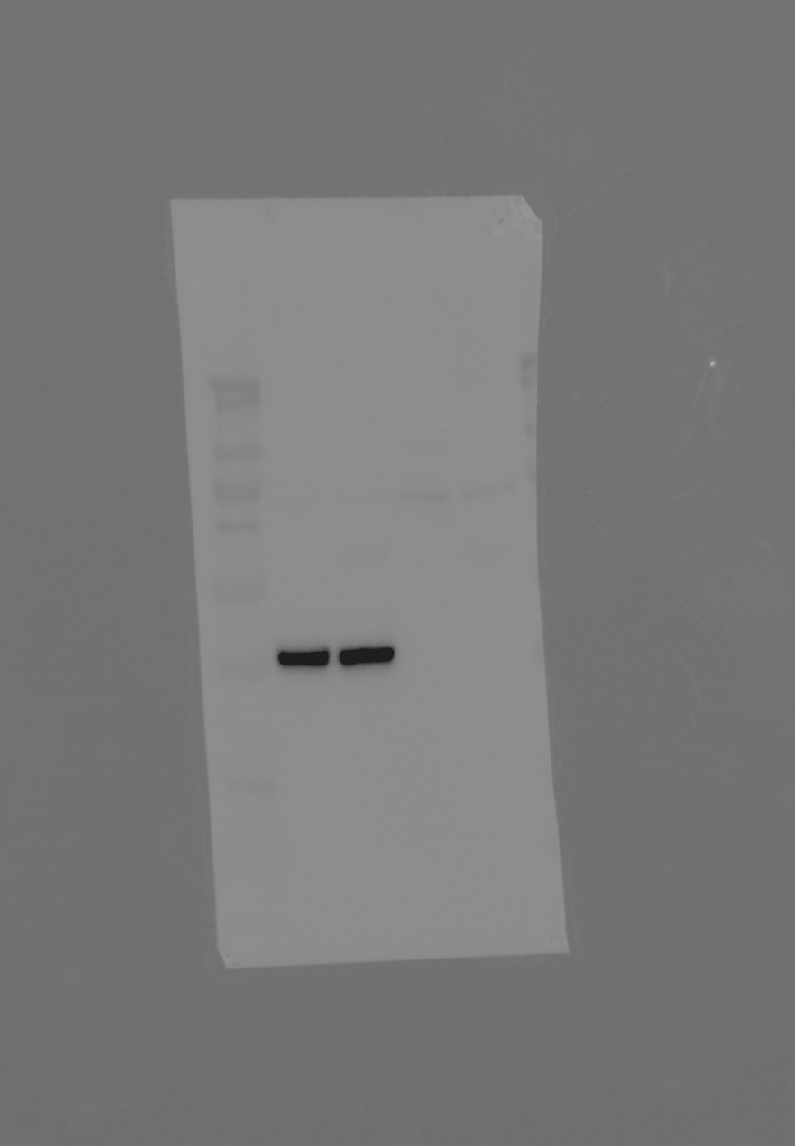

Supplement: Supplementary file 4 — Source Data Fig. 1 [file 44319_2023_27_MOESM4_ESM.zip › Figure 1/1C/western UBXN1.tif]

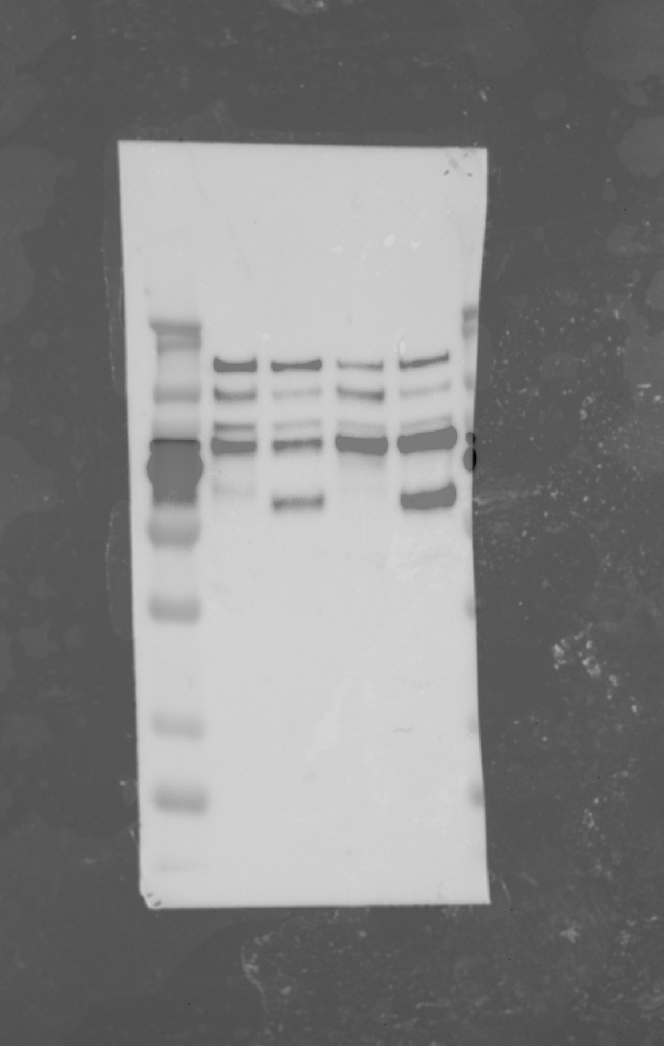

Supplement: Supplementary file 4 — Source Data Fig. 1 [file 44319_2023_27_MOESM4_ESM.zip › Figure 1/1C/Western ATF6.tif]

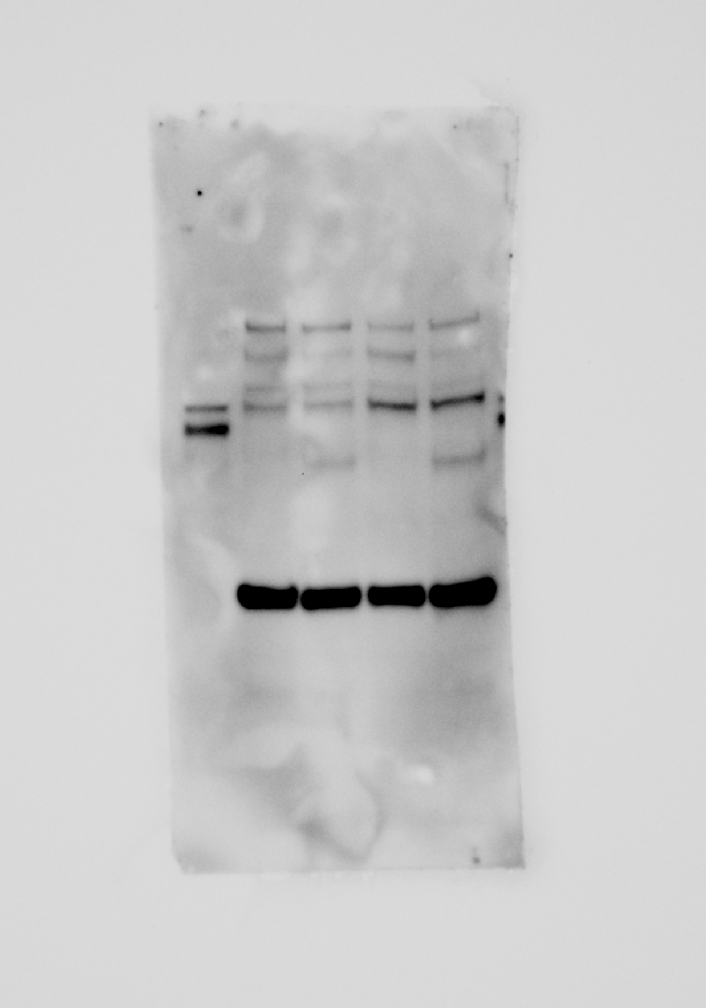

Supplement: Supplementary file 4 — Source Data Fig. 1 [file 44319_2023_27_MOESM4_ESM.zip › Figure 1/1C/western GAPDH.tif]

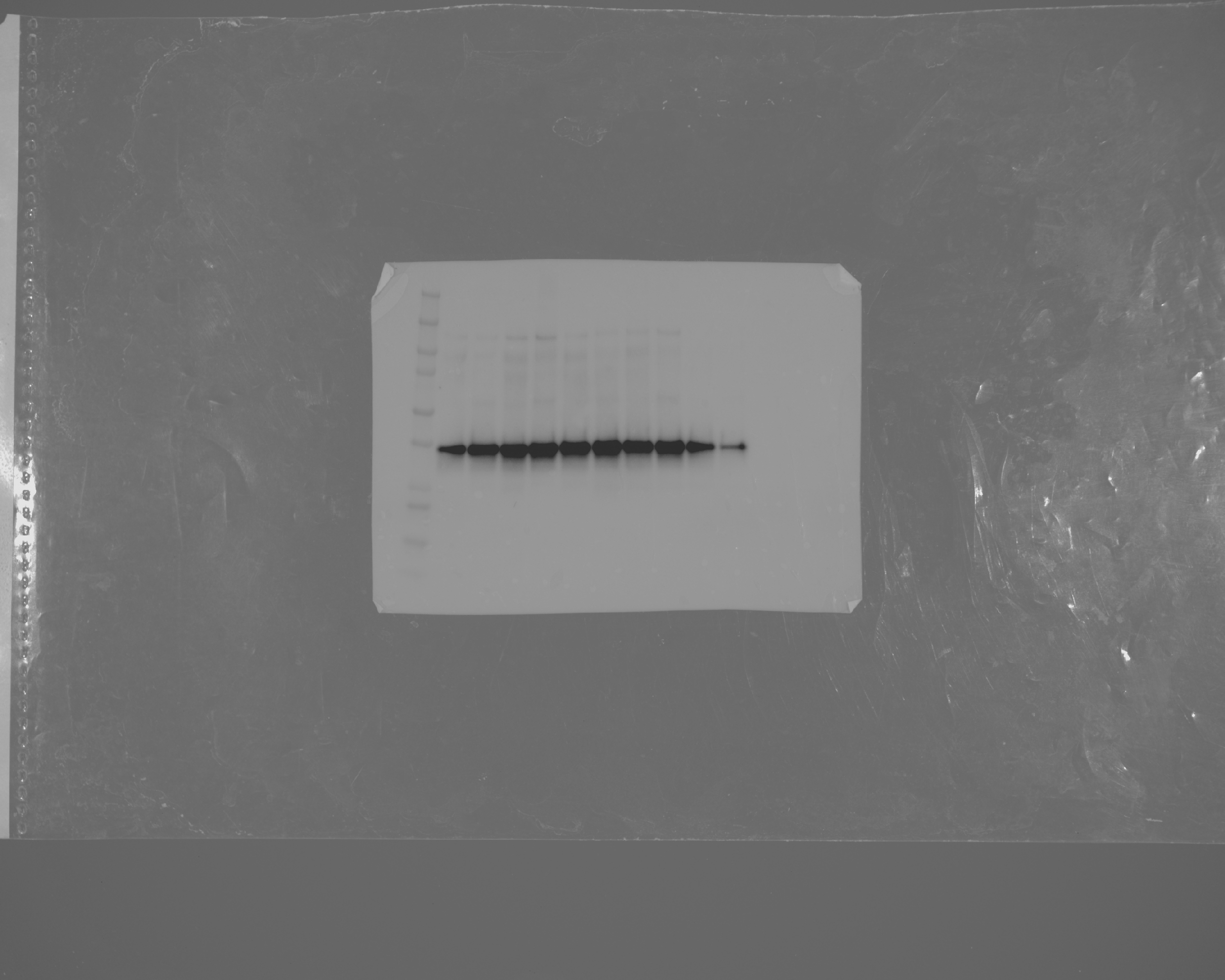

Supplement: Supplementary file 5 — Source Data Fig. 2 [file 44319_2023_27_MOESM5_ESM.zip › Figure 2/2D/Western GAPDH.jpg]

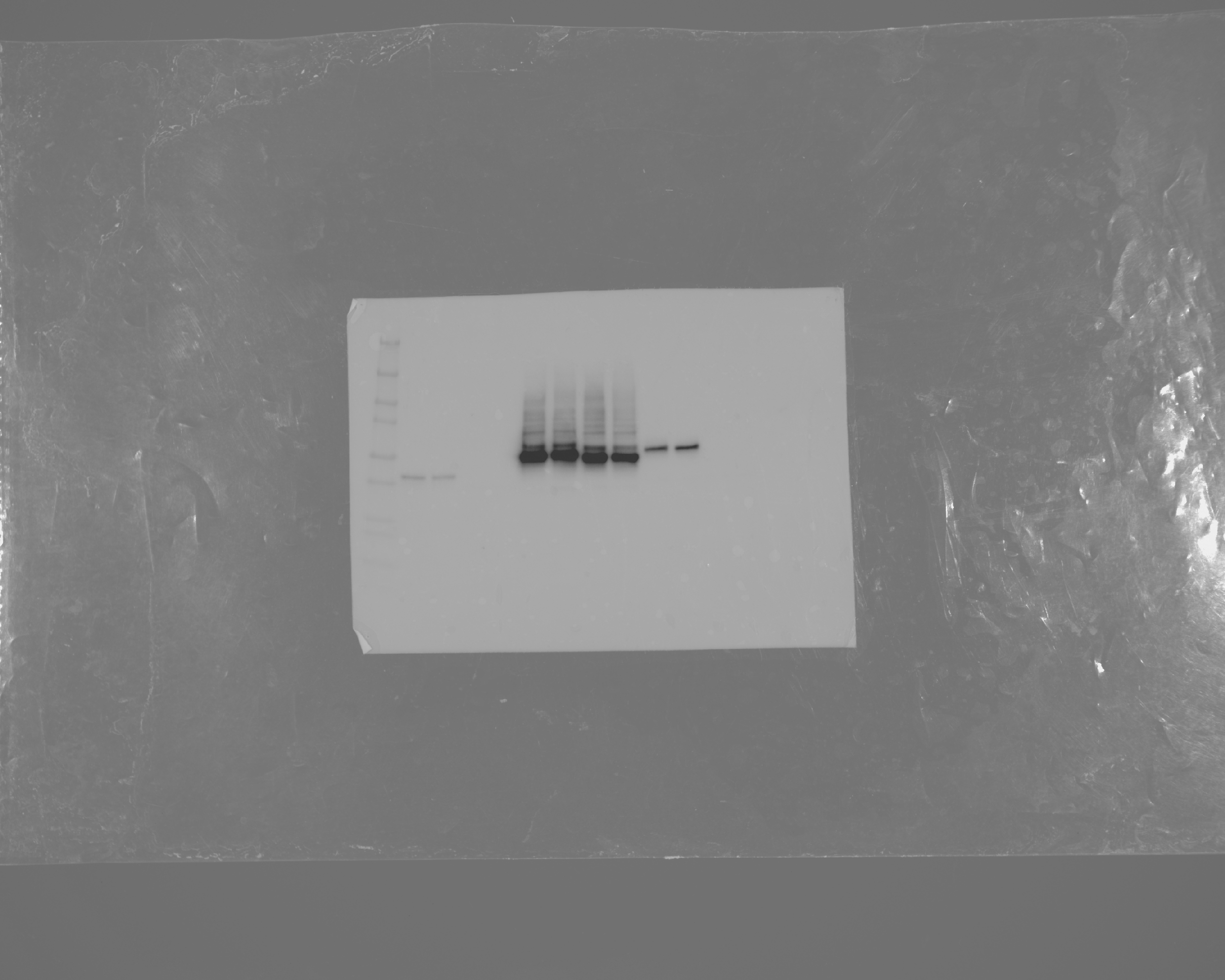

Supplement: Supplementary file 5 — Source Data Fig. 2 [file 44319_2023_27_MOESM5_ESM.zip › Figure 2/2D/Western Myc-UBXN1.jpg]

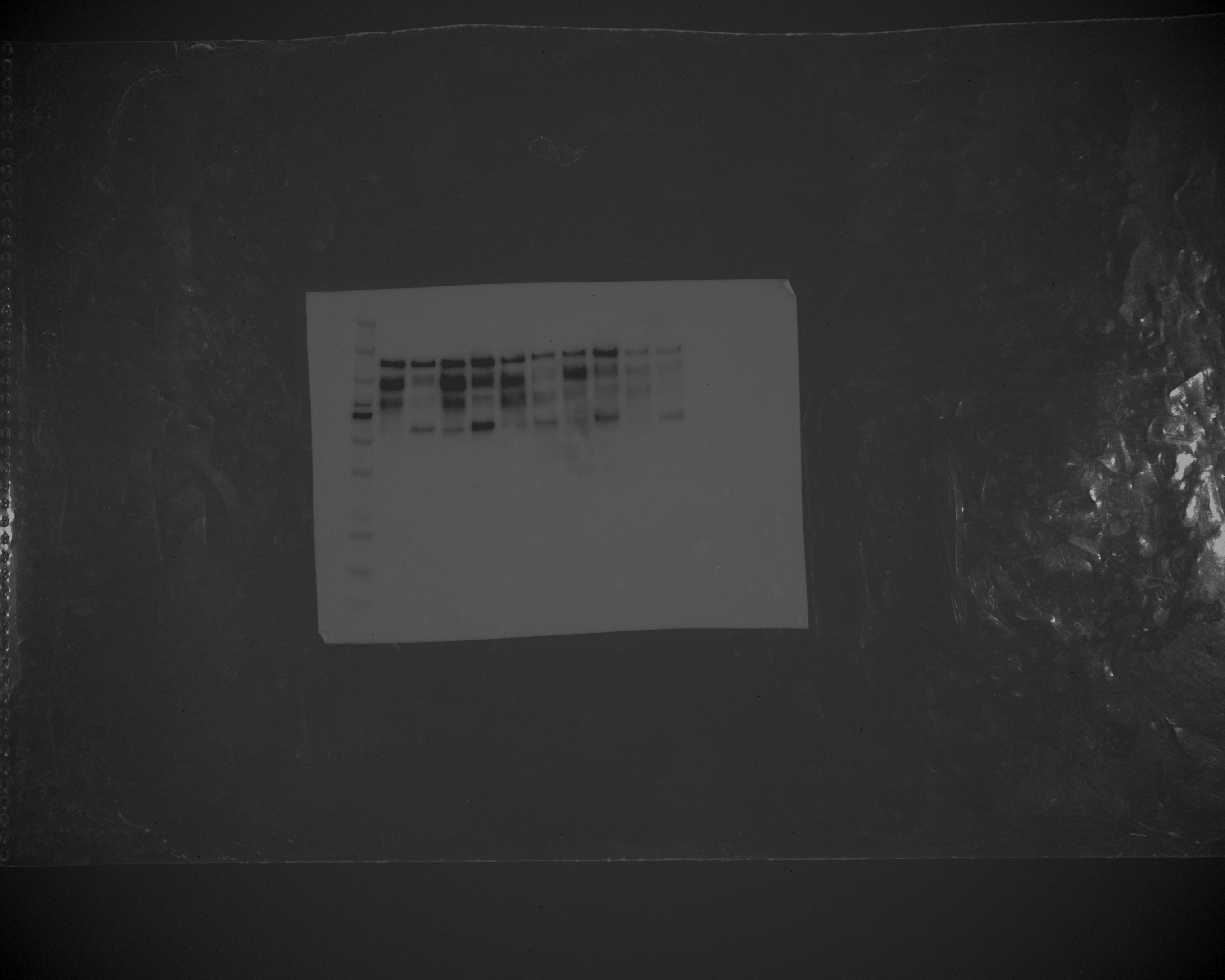

Supplement: Supplementary file 5 — Source Data Fig. 2 [file 44319_2023_27_MOESM5_ESM.zip › Figure 2/2D/Western ATF6.jpg]

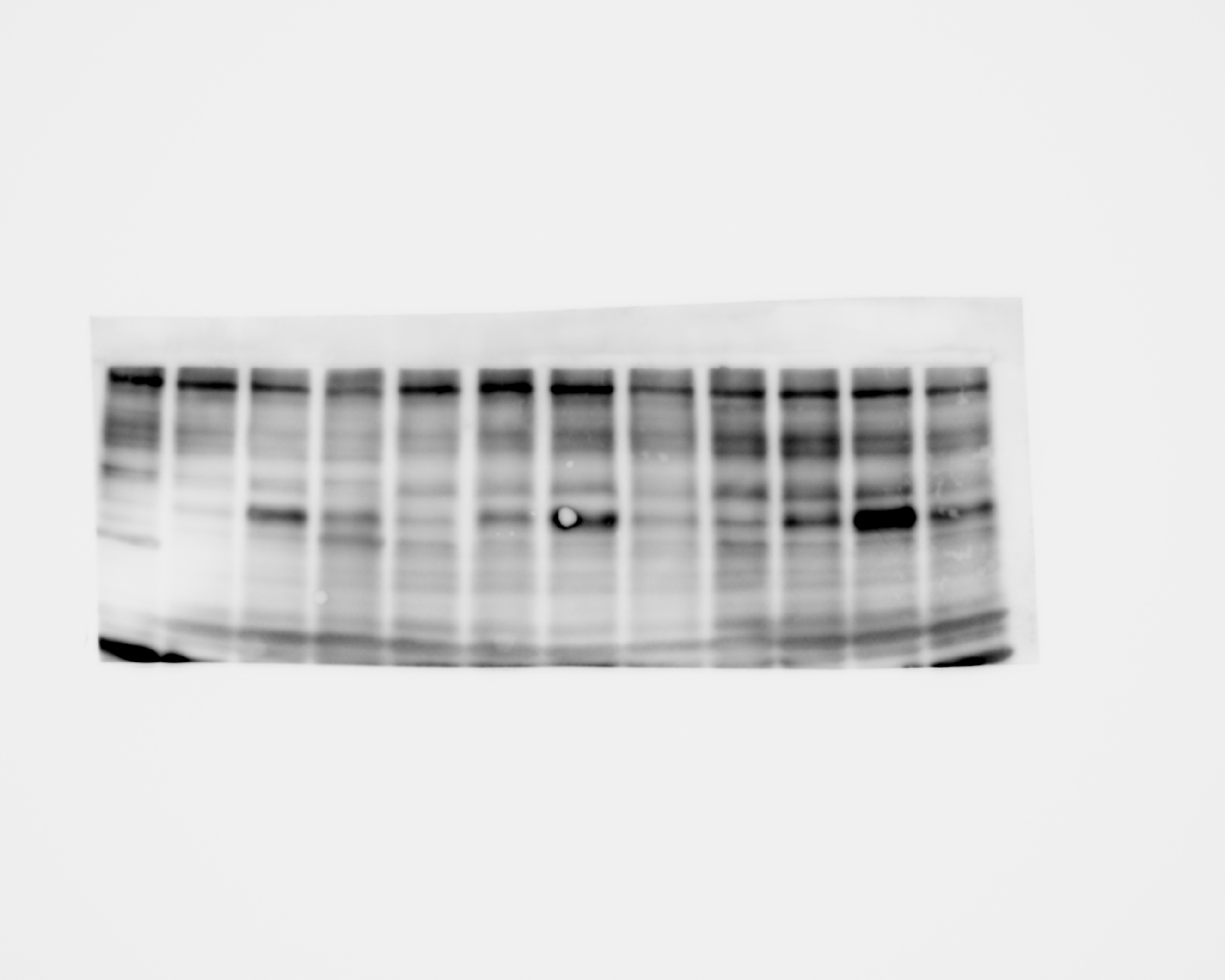

Supplement: Supplementary file 6 — Source Data Fig. 3 [file 44319_2023_27_MOESM6_ESM.zip › Figure 3/3D/western CHOP.tif]

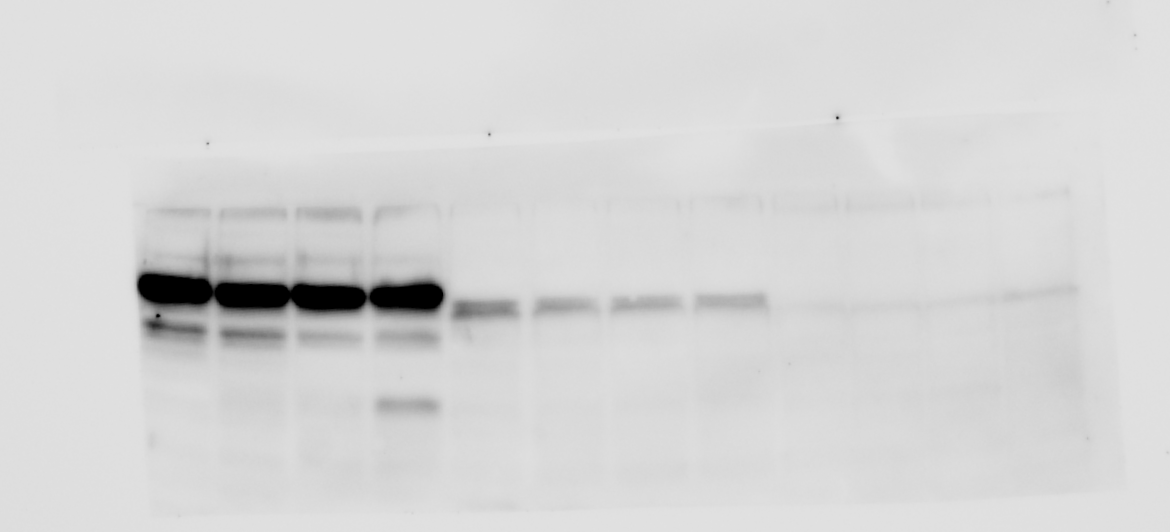

Supplement: Supplementary file 6 — Source Data Fig. 3 [file 44319_2023_27_MOESM6_ESM.zip › Figure 3/3D/western UBXN1.tif]

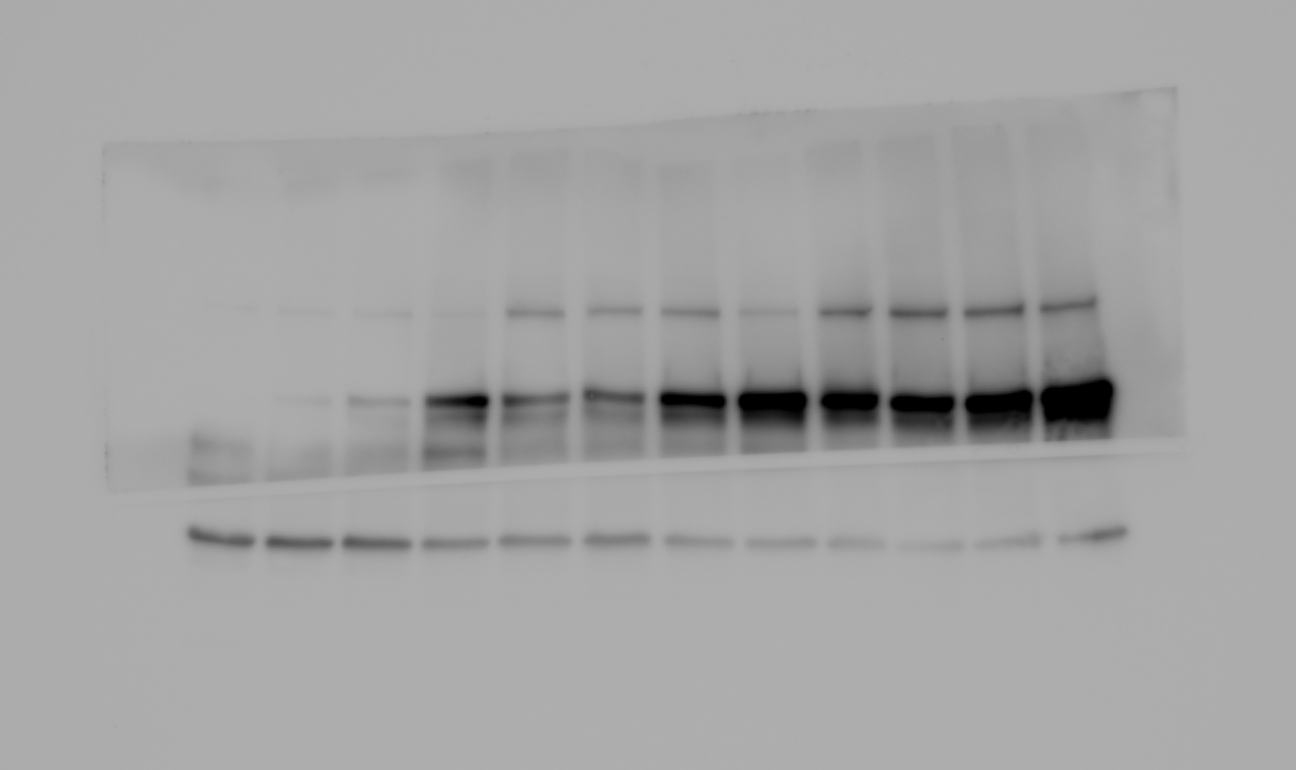

Supplement: Supplementary file 6 — Source Data Fig. 3 [file 44319_2023_27_MOESM6_ESM.zip › Figure 3/3D/western GAPDH.tif]

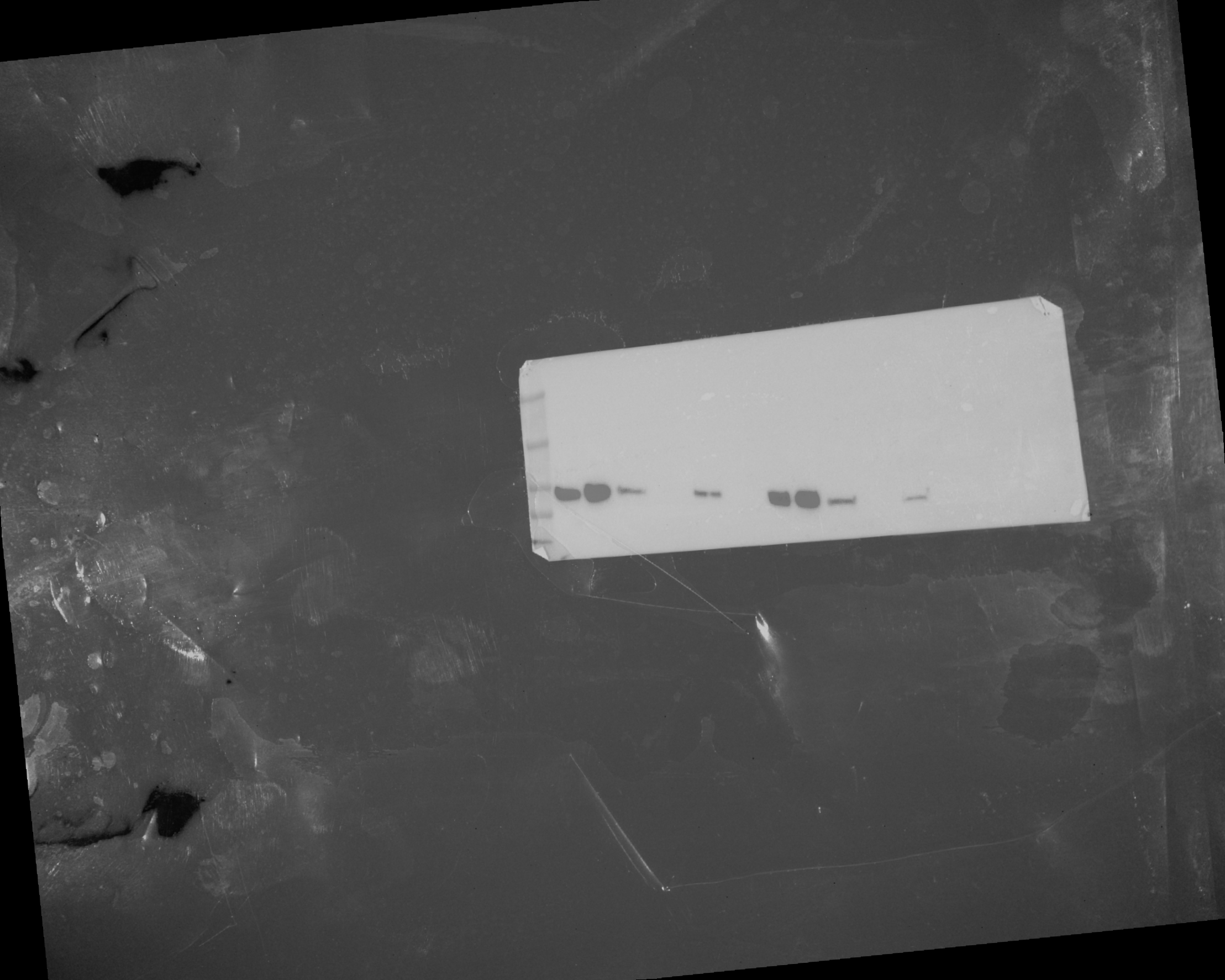

Supplement: Supplementary file 6 — Source Data Fig. 3 [file 44319_2023_27_MOESM6_ESM.zip › Figure 3/EV2C/Western p97.tif]

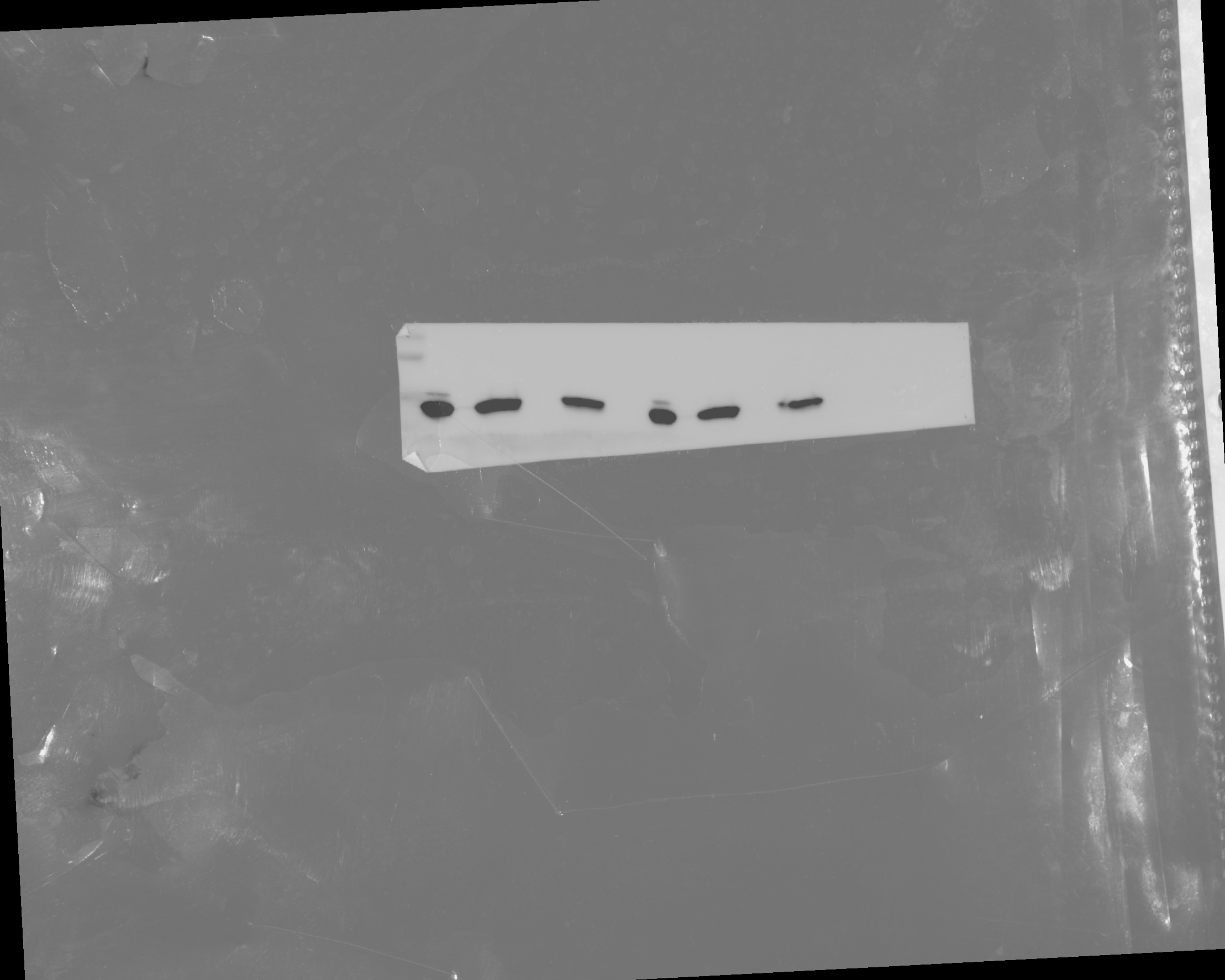

Supplement: Supplementary file 6 — Source Data Fig. 3 [file 44319_2023_27_MOESM6_ESM.zip › Figure 3/EV2C/Western SEC61B.tif]

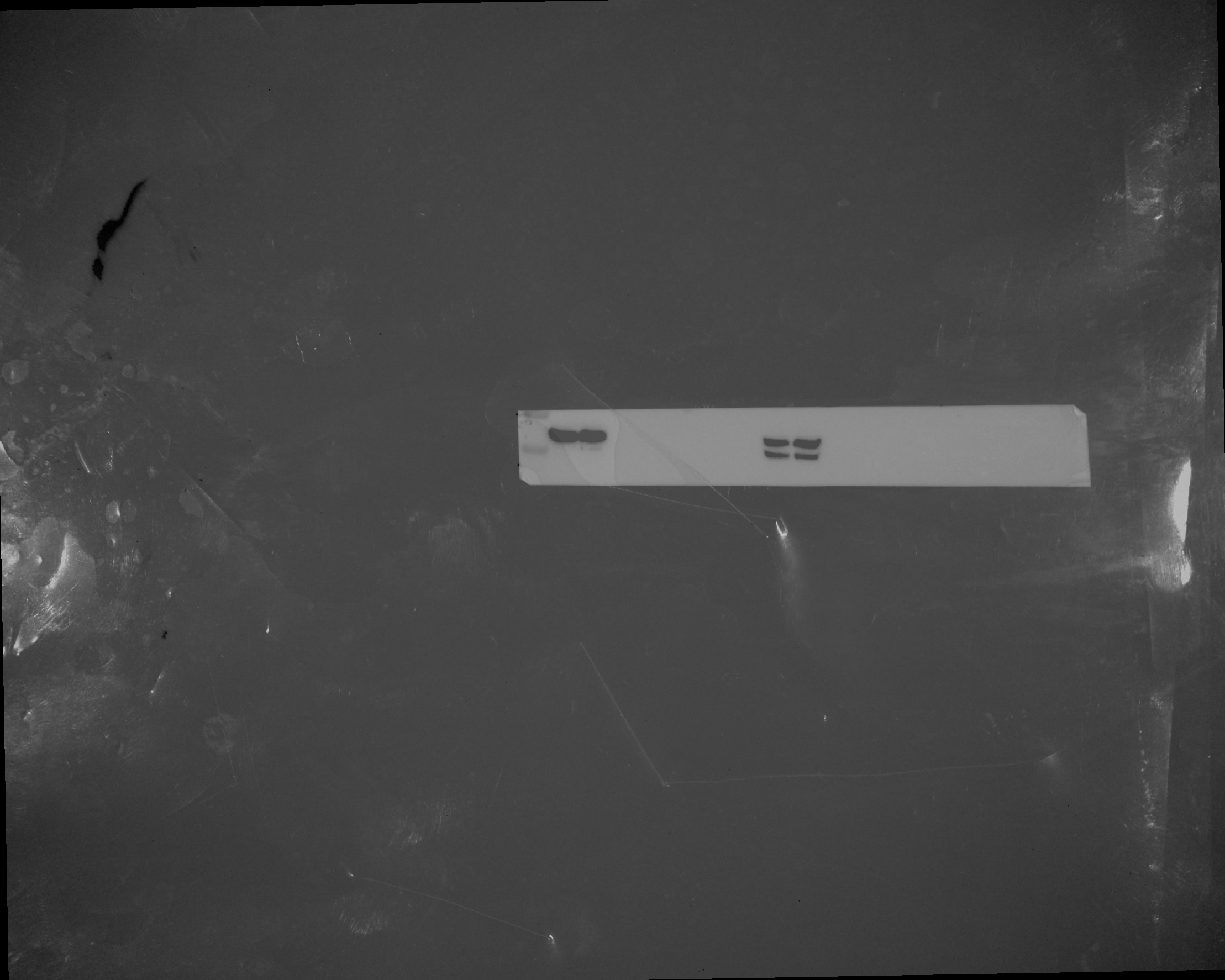

Supplement: Supplementary file 6 — Source Data Fig. 3 [file 44319_2023_27_MOESM6_ESM.zip › Figure 3/EV2C/Western Bactin.tif]

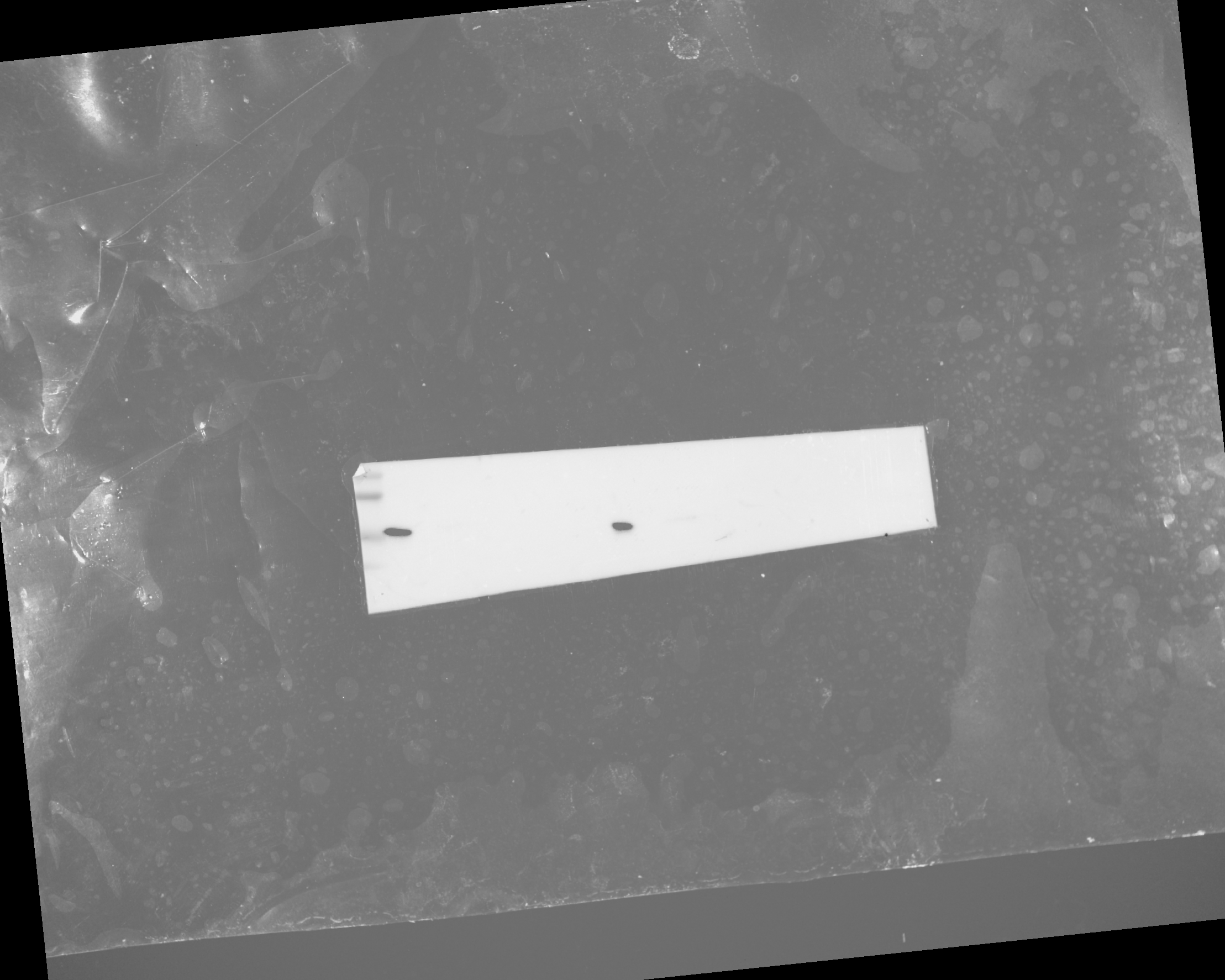

Supplement: Supplementary file 6 — Source Data Fig. 3 [file 44319_2023_27_MOESM6_ESM.zip › Figure 3/EV2C/Western TOMM20.tif]

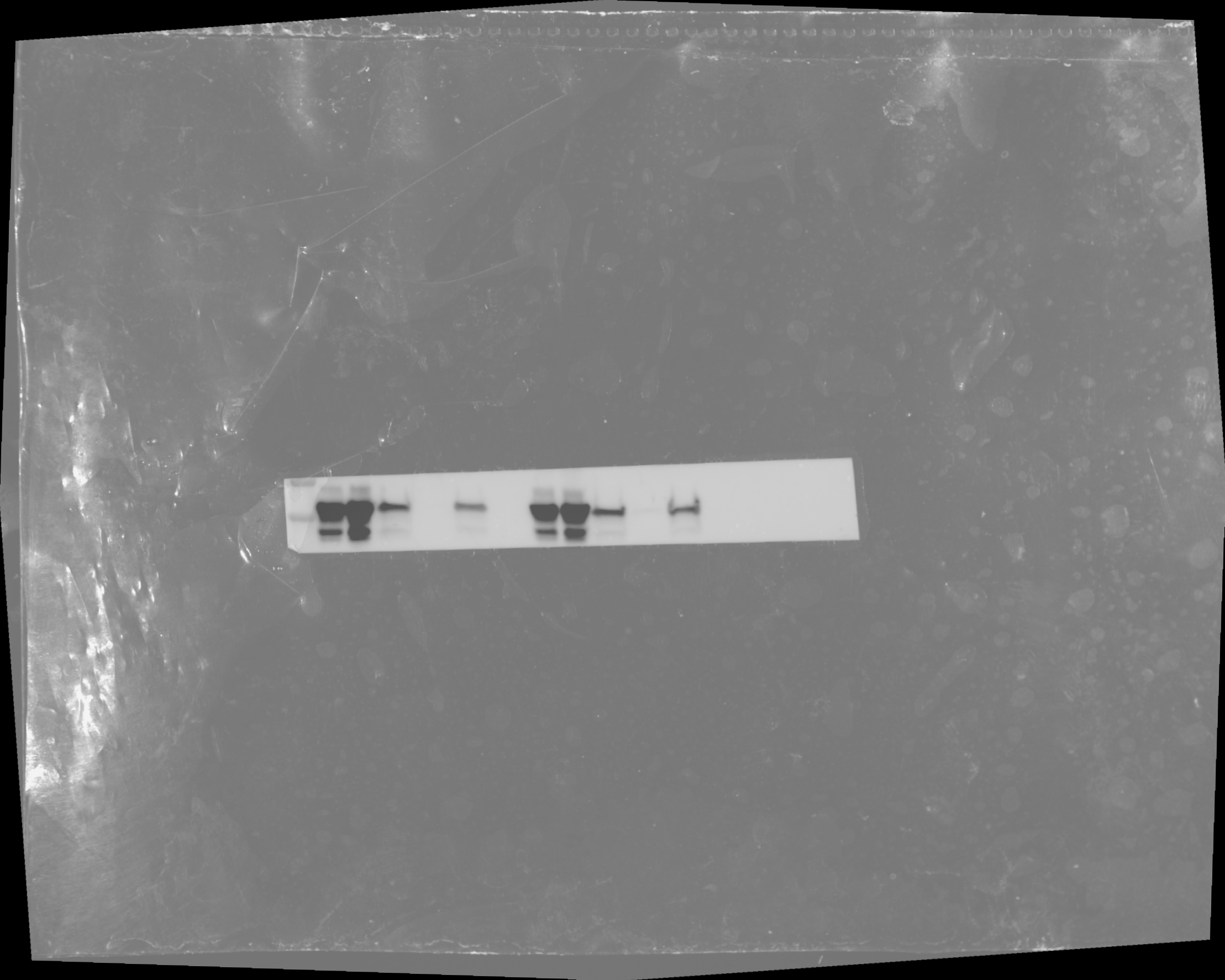

Supplement: Supplementary file 6 — Source Data Fig. 3 [file 44319_2023_27_MOESM6_ESM.zip › Figure 3/EV2C/Western UBXN1.tif]

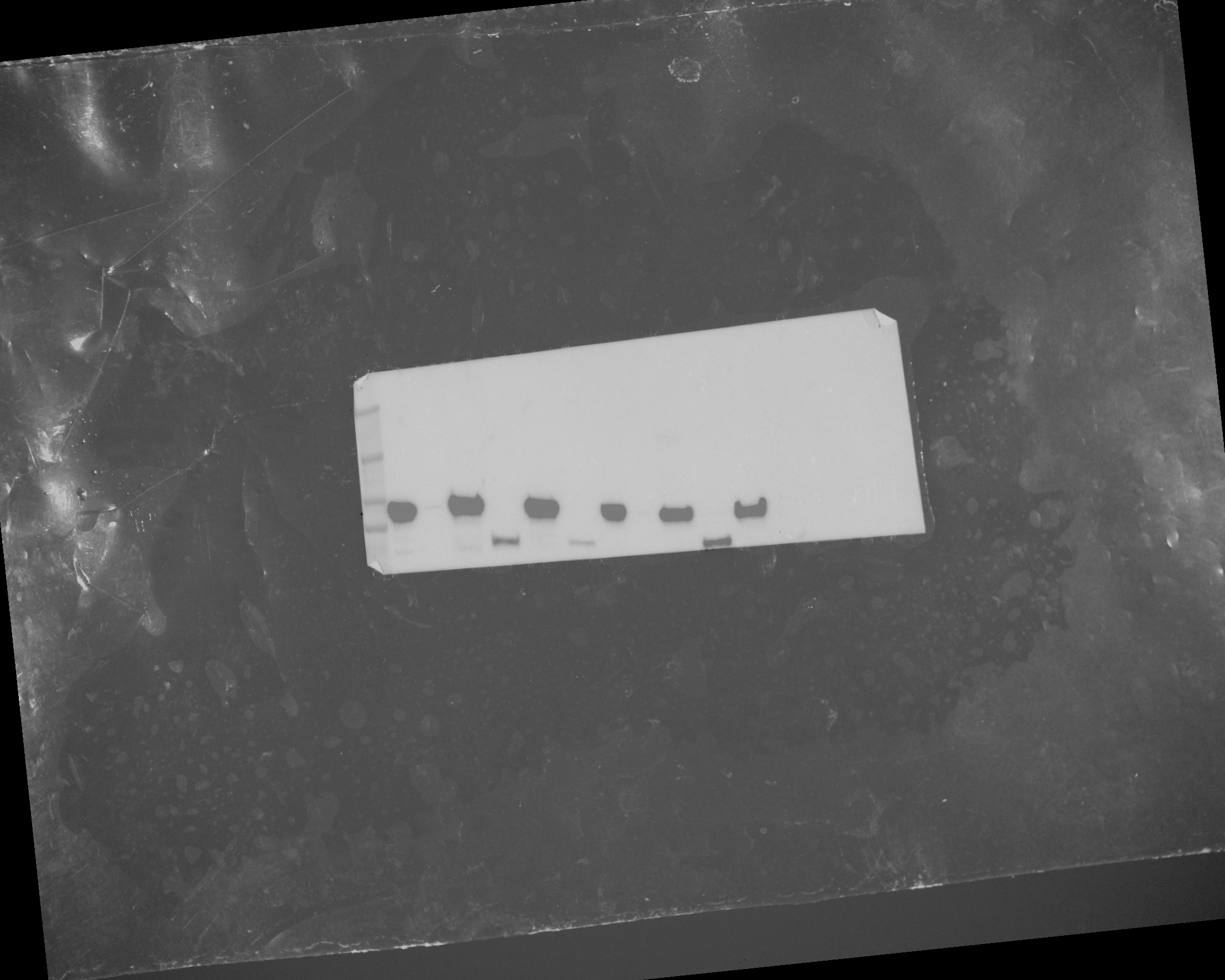

Supplement: Supplementary file 6 — Source Data Fig. 3 [file 44319_2023_27_MOESM6_ESM.zip › Figure 3/EV2C/Western Calnexin.tif]

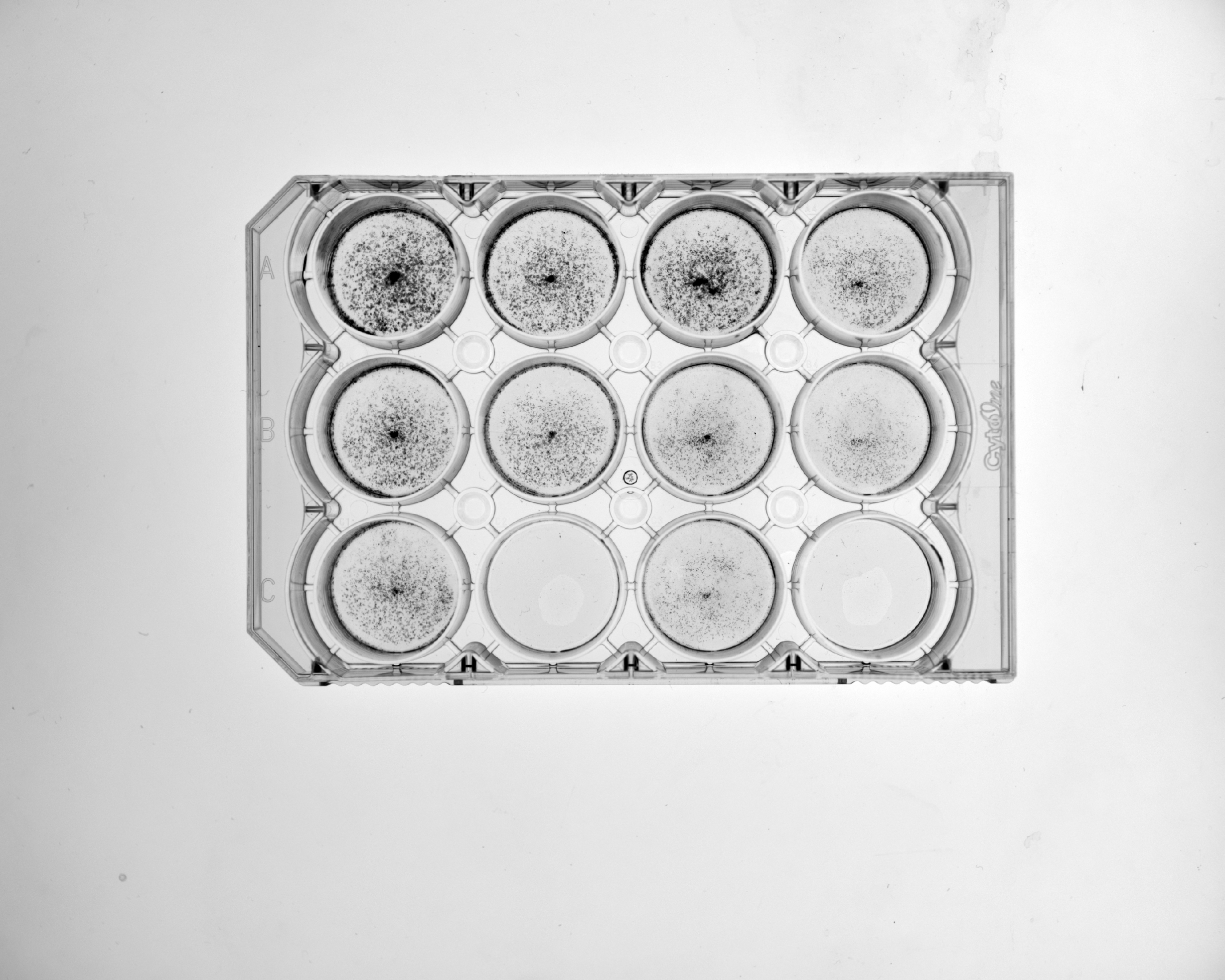

Supplement: Supplementary file 6 — Source Data Fig. 3 [file 44319_2023_27_MOESM6_ESM.zip › Figure 3/3A/Tissue culture plate picture_crystal violet.jpg]

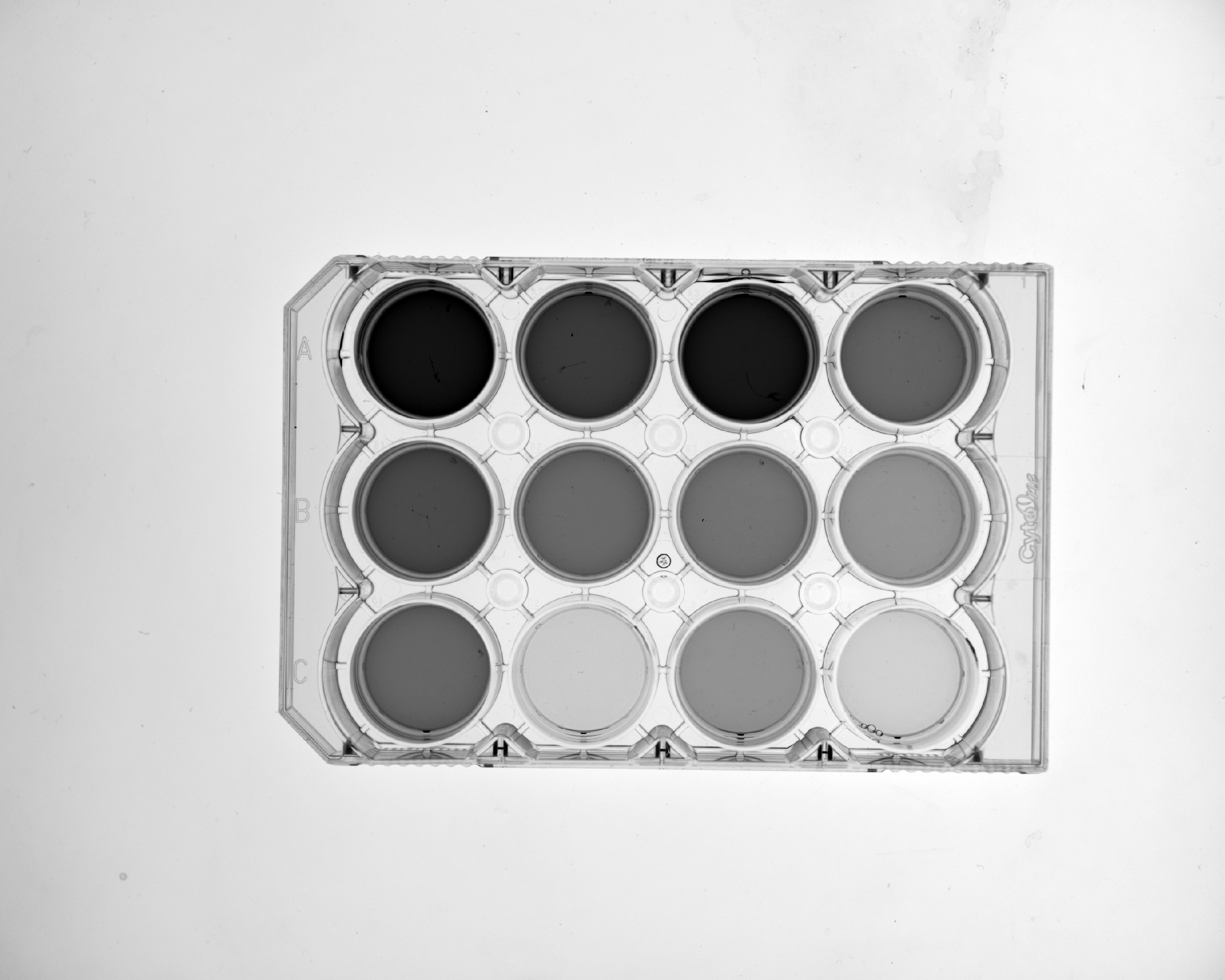

Supplement: Supplementary file 6 — Source Data Fig. 3 [file 44319_2023_27_MOESM6_ESM.zip › Figure 3/3A/tissue culture plate picture_solubilized.jpg]

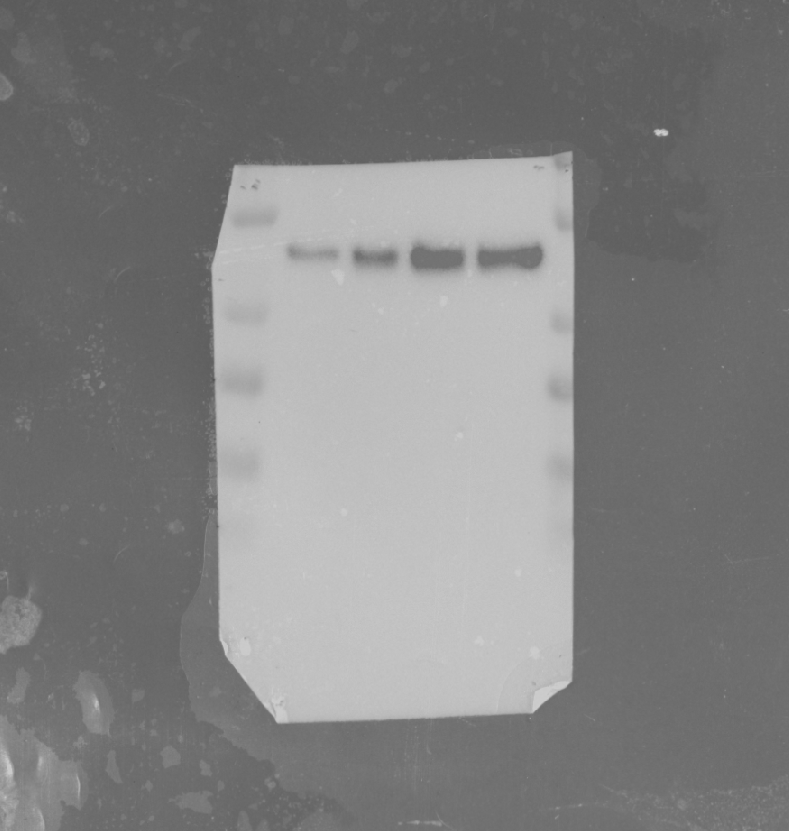

Supplement: Supplementary file 7 — Source Data Fig. 5 [file 44319_2023_27_MOESM7_ESM.zip › Figure 5/5A/western TRAP alpha.tif]

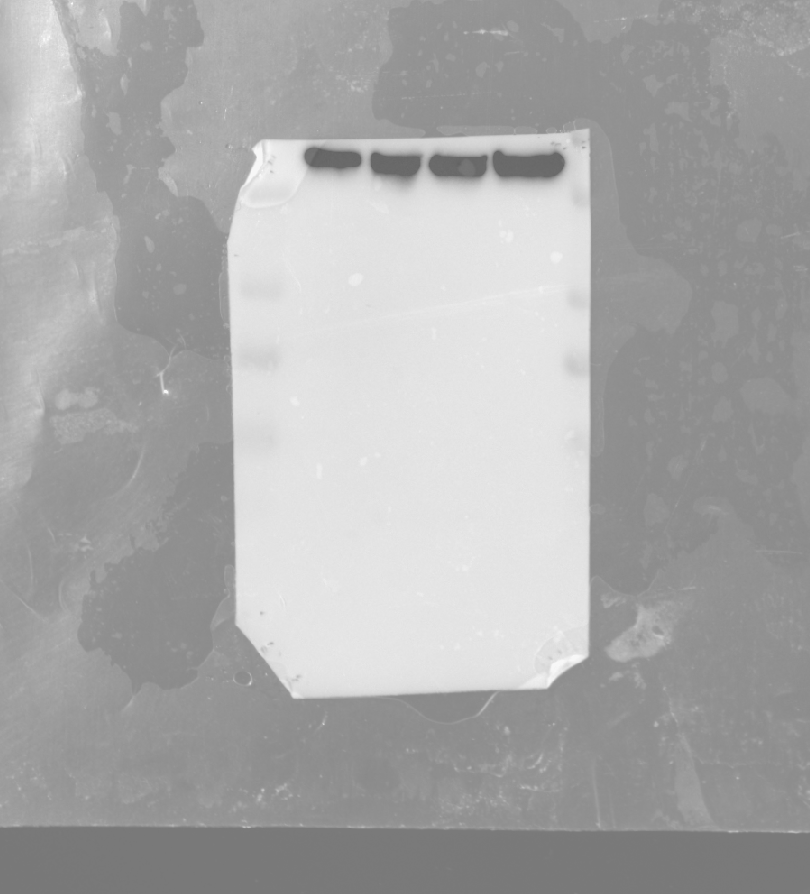

Supplement: Supplementary file 7 — Source Data Fig. 5 [file 44319_2023_27_MOESM7_ESM.zip › Figure 5/5A/western Bactin.tif]

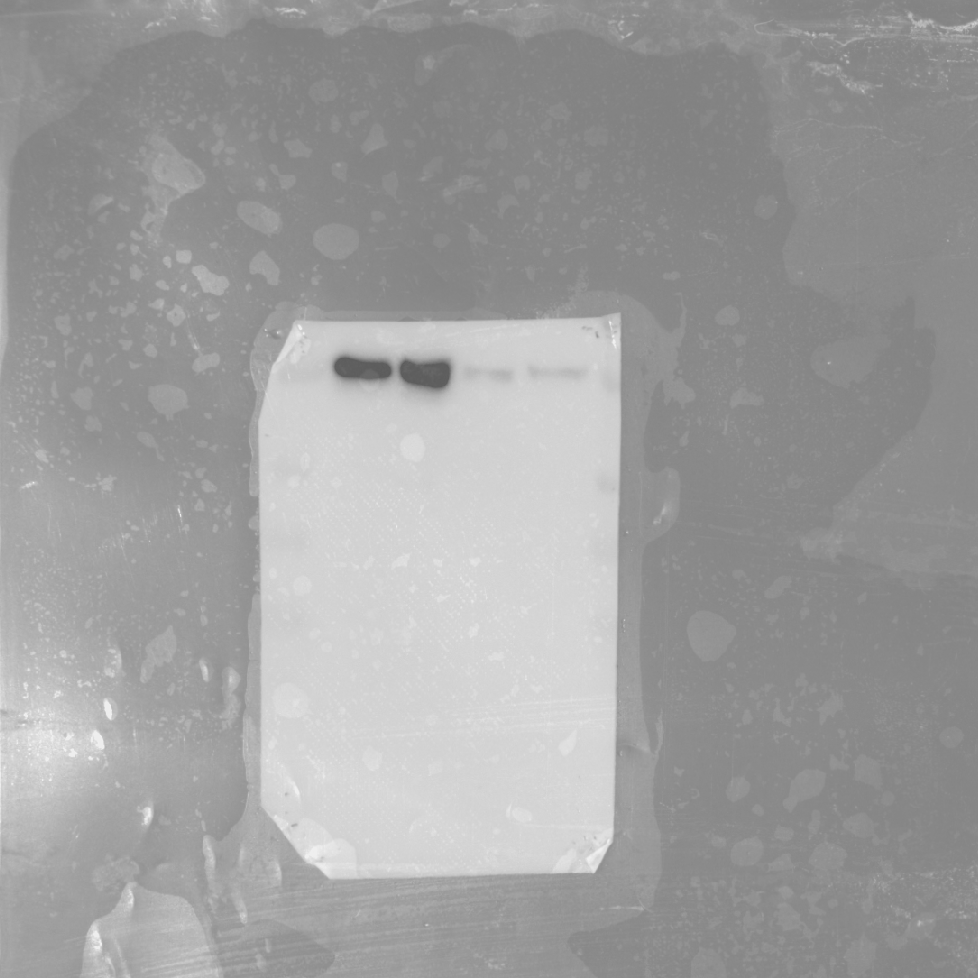

Supplement: Supplementary file 7 — Source Data Fig. 5 [file 44319_2023_27_MOESM7_ESM.zip › Figure 5/5A/western UBXN1.tif]

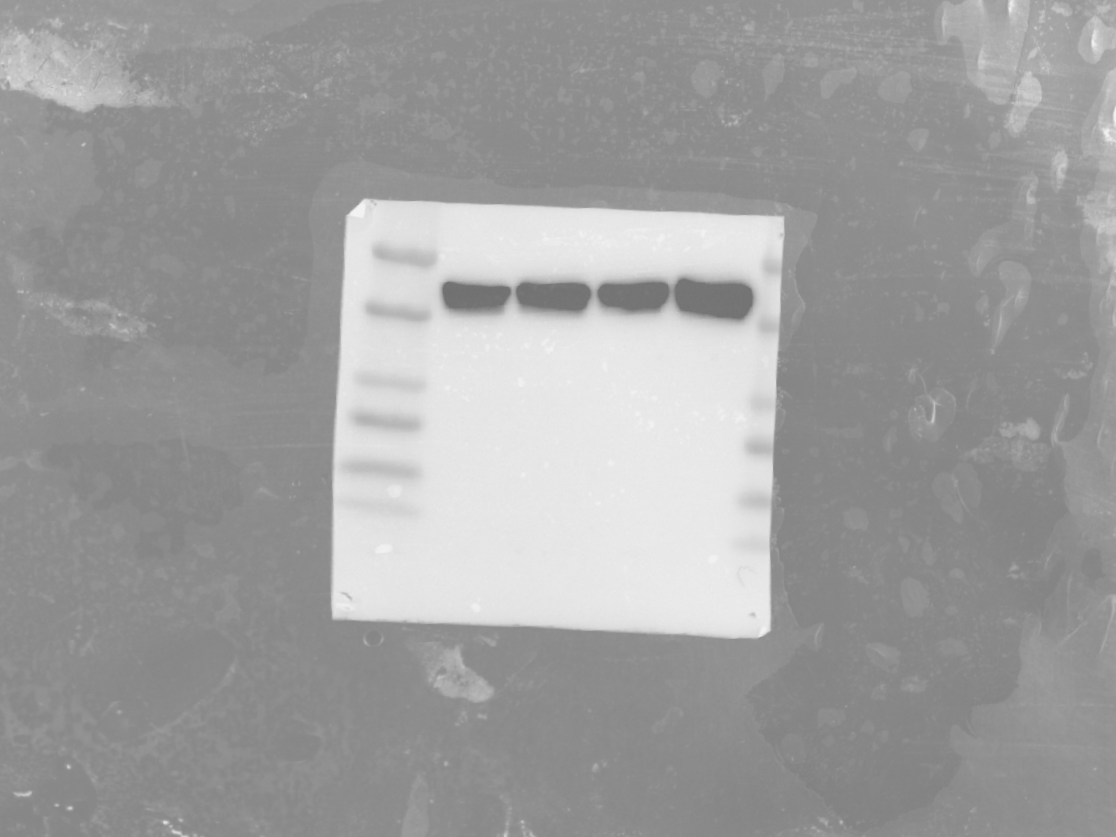

Supplement: Supplementary file 7 — Source Data Fig. 5 [file 44319_2023_27_MOESM7_ESM.zip › Figure 5/5F/western bactin.tif]

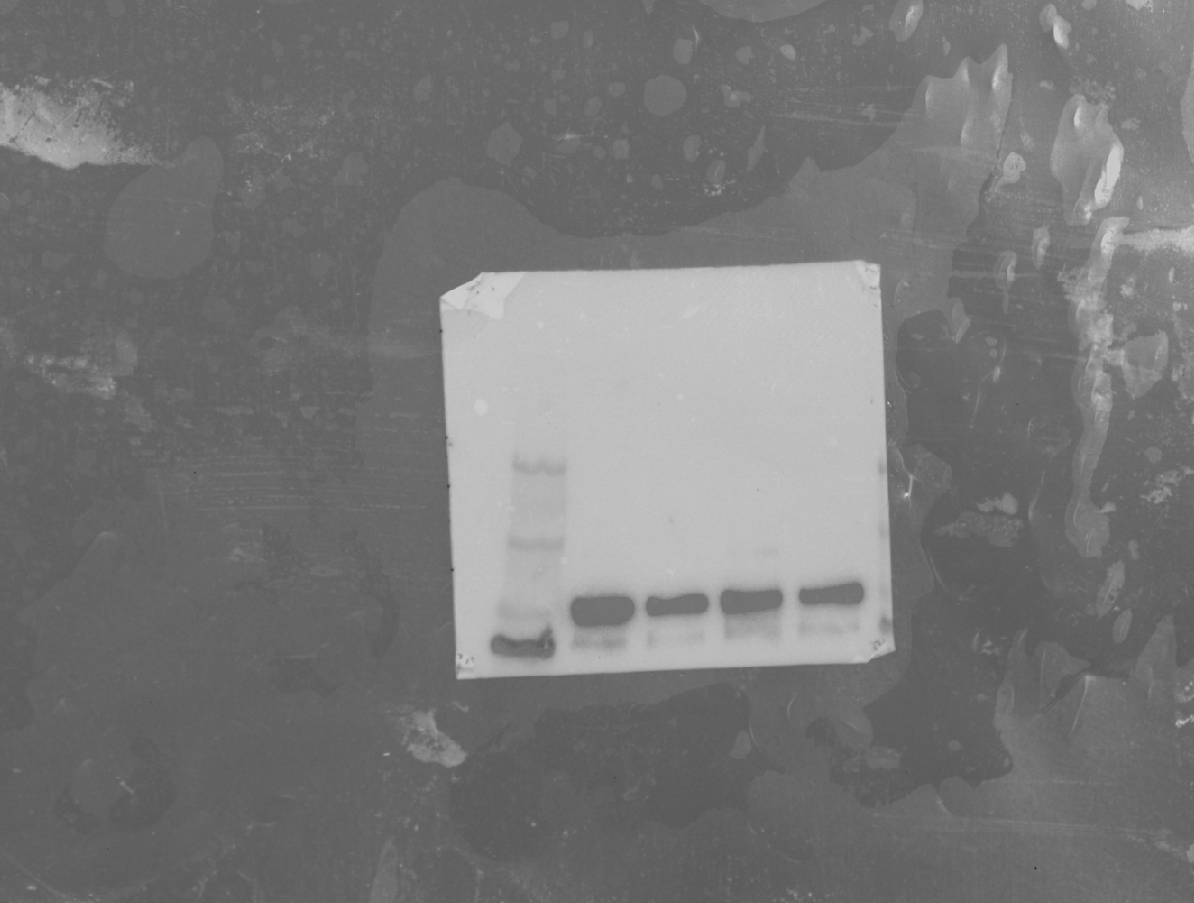

Supplement: Supplementary file 7 — Source Data Fig. 5 [file 44319_2023_27_MOESM7_ESM.zip › Figure 5/5F/western MCM3.tif]

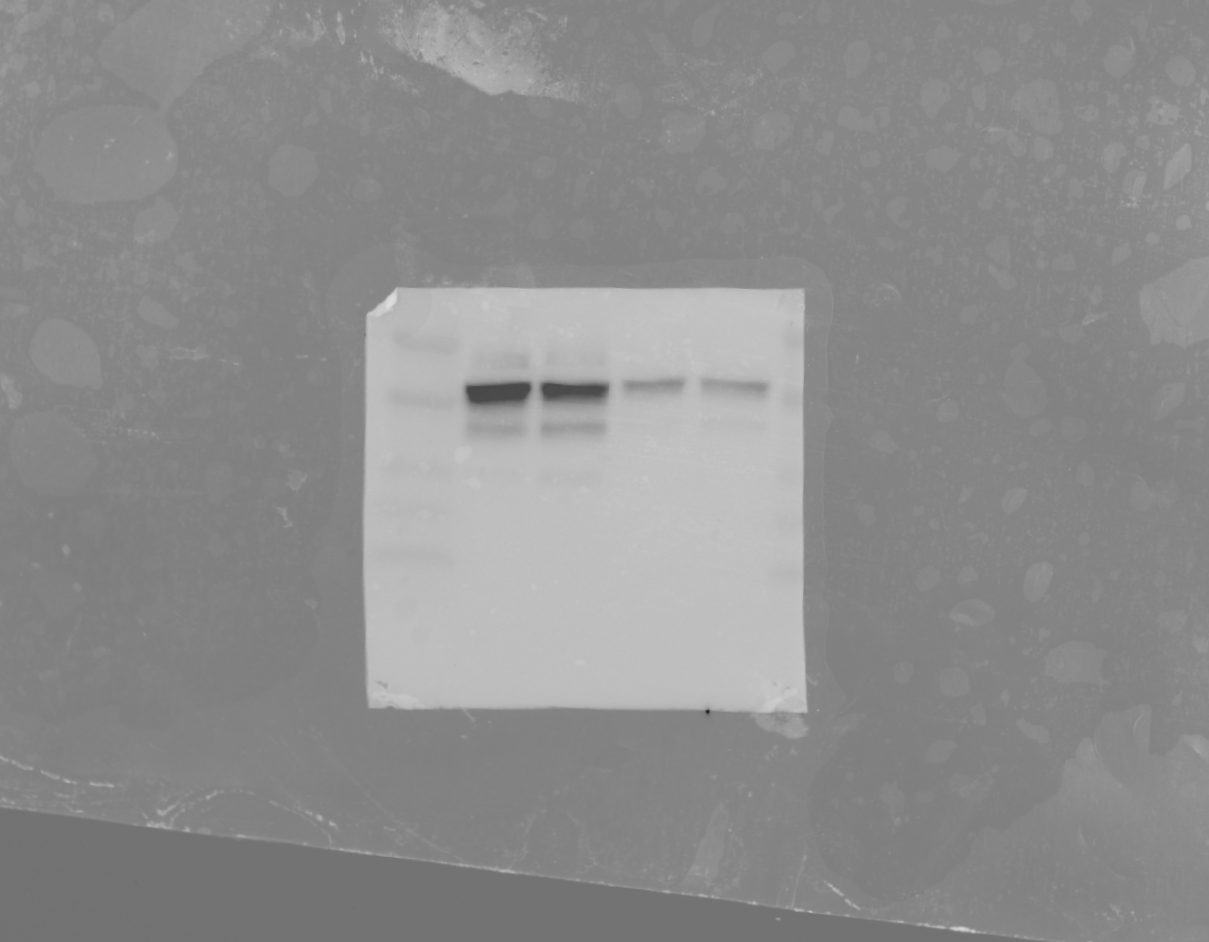

Supplement: Supplementary file 7 — Source Data Fig. 5 [file 44319_2023_27_MOESM7_ESM.zip › Figure 5/5F/western UBXN1.tif]

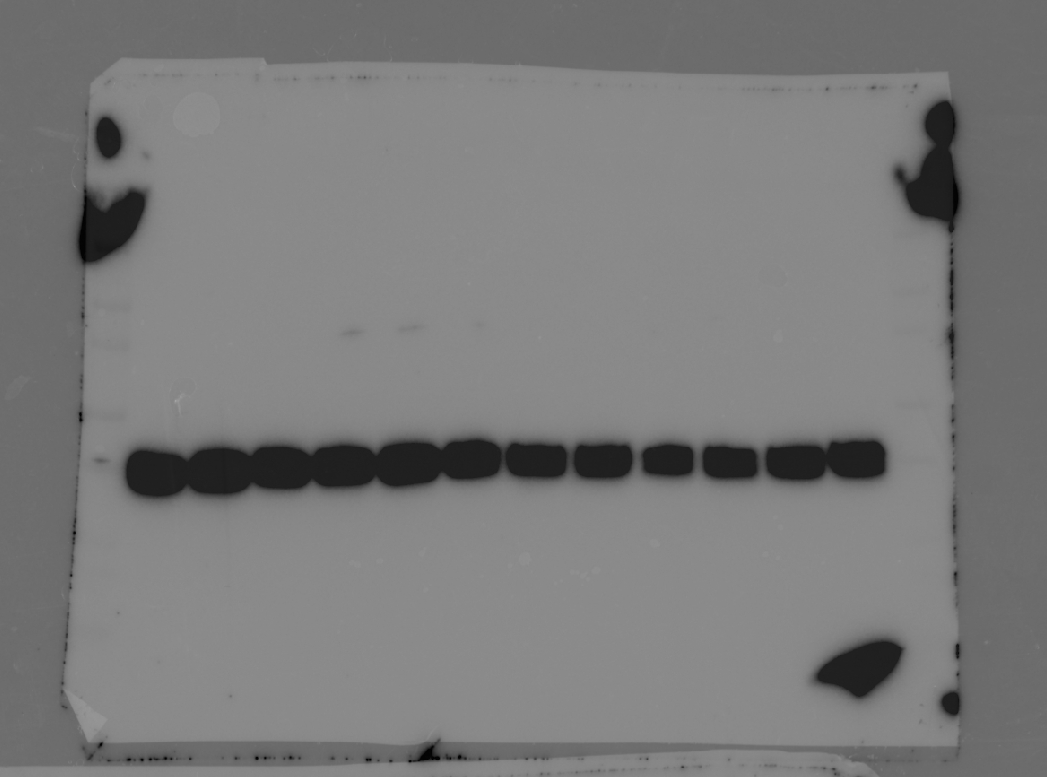

Supplement: Supplementary file 7 — Source Data Fig. 5 [file 44319_2023_27_MOESM7_ESM.zip › Figure 5/5H/Western Bactin.tif]

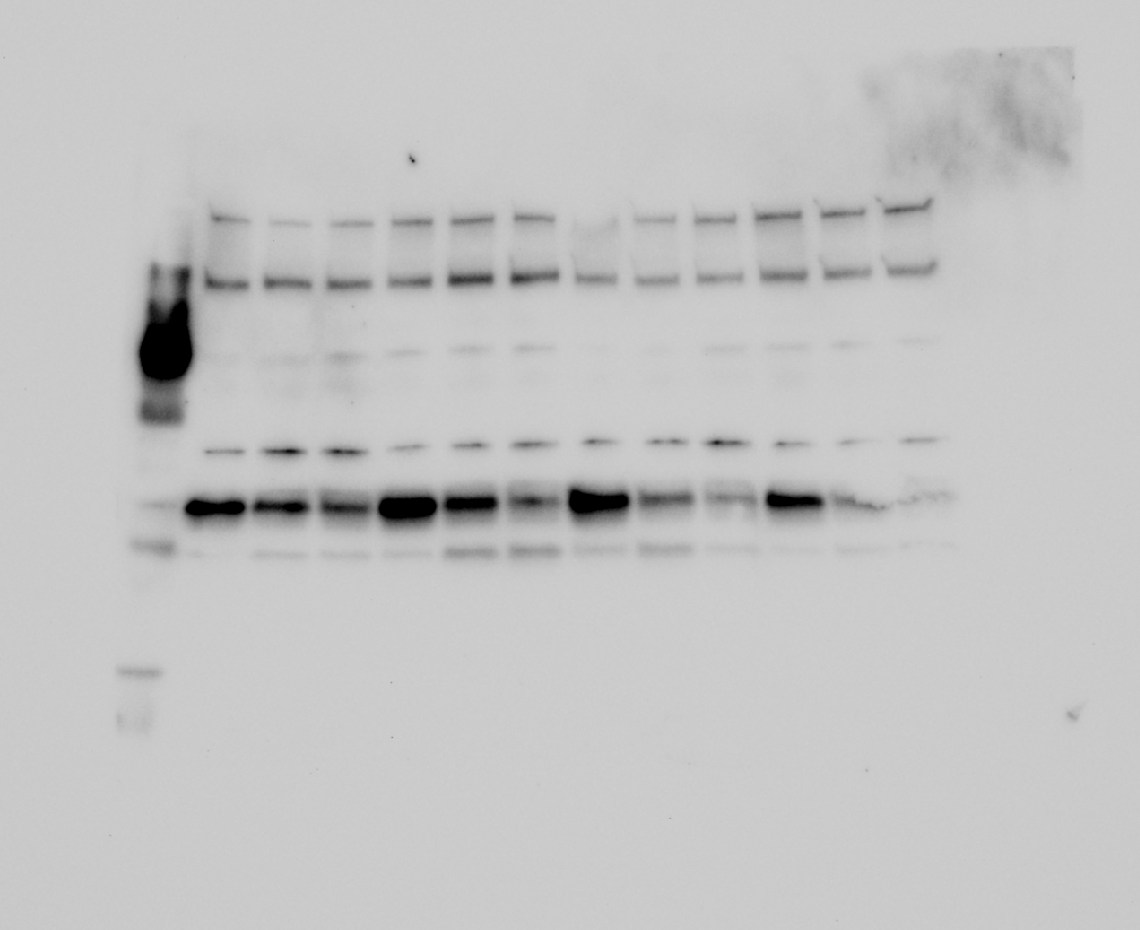

Supplement: Supplementary file 7 — Source Data Fig. 5 [file 44319_2023_27_MOESM7_ESM.zip › Figure 5/5H/Western SCD1.tif]

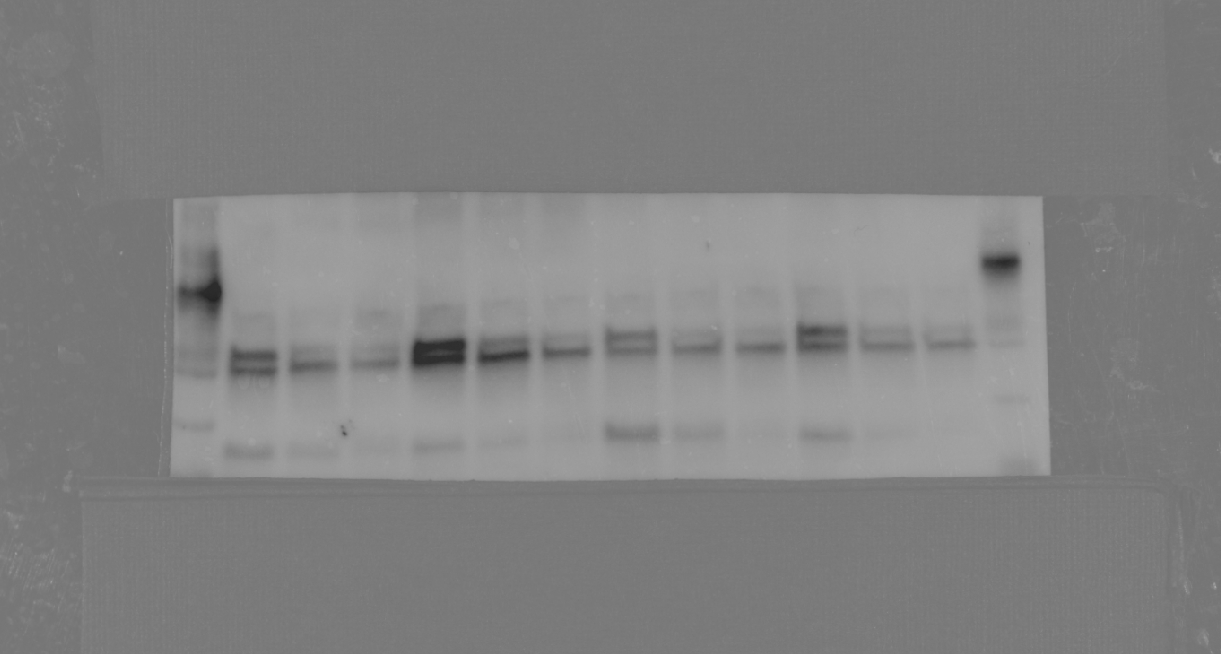

Supplement: Supplementary file 7 — Source Data Fig. 5 [file 44319_2023_27_MOESM7_ESM.zip › Figure 5/5H/Western SQLE.tif]

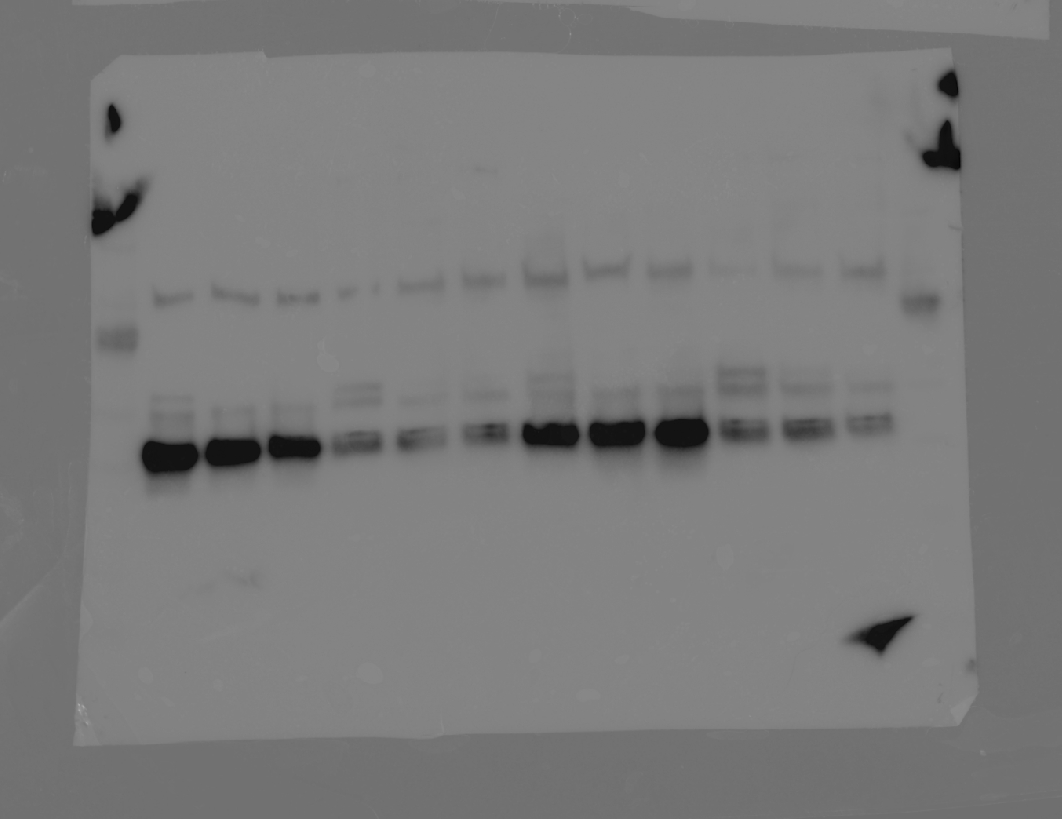

Supplement: Supplementary file 7 — Source Data Fig. 5 [file 44319_2023_27_MOESM7_ESM.zip › Figure 5/5H/Western UBXN1.tif]

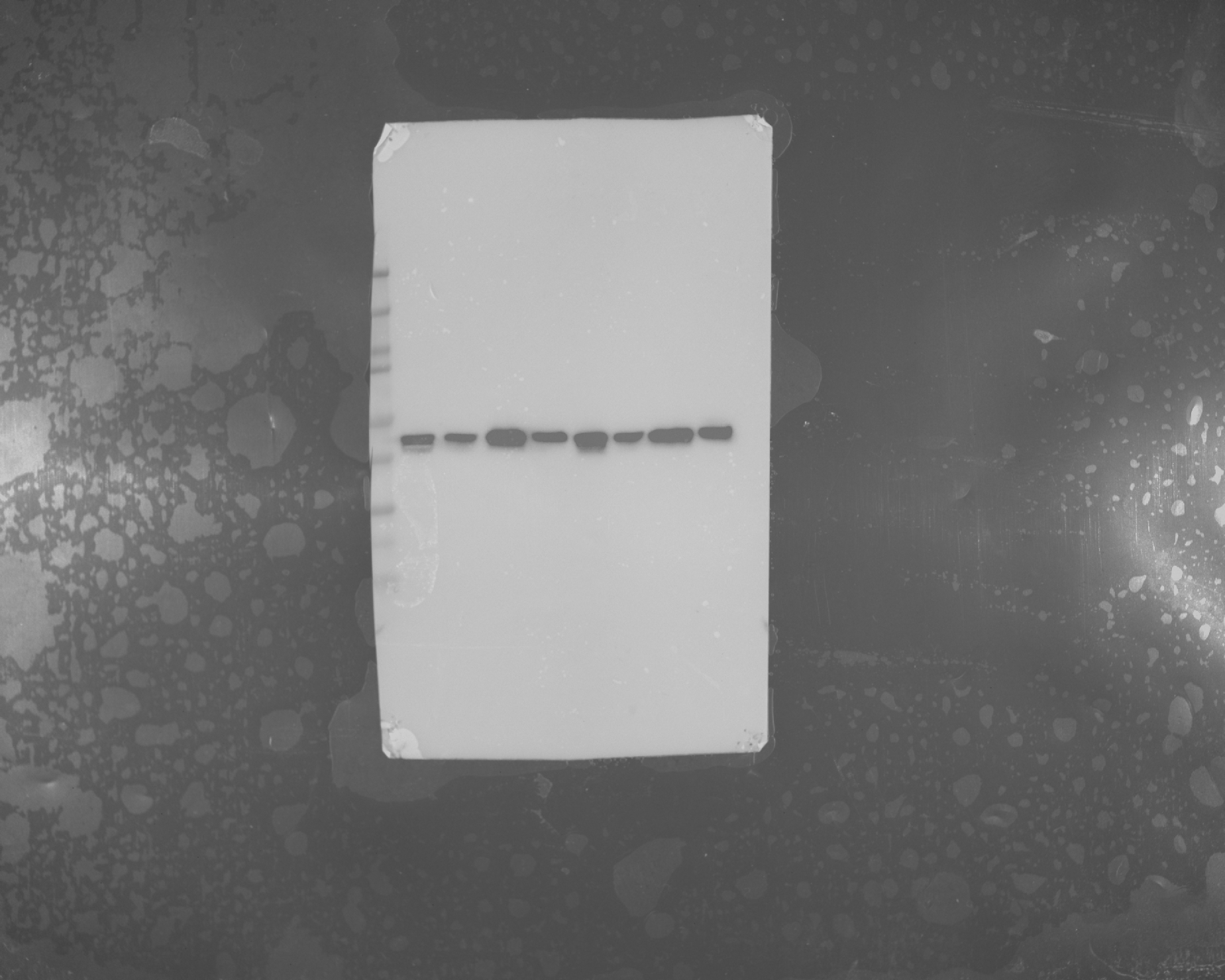

Supplement: Supplementary file 7 — Source Data Fig. 5 [file 44319_2023_27_MOESM7_ESM.zip › Figure 5/5J/Western Bactin.tif]

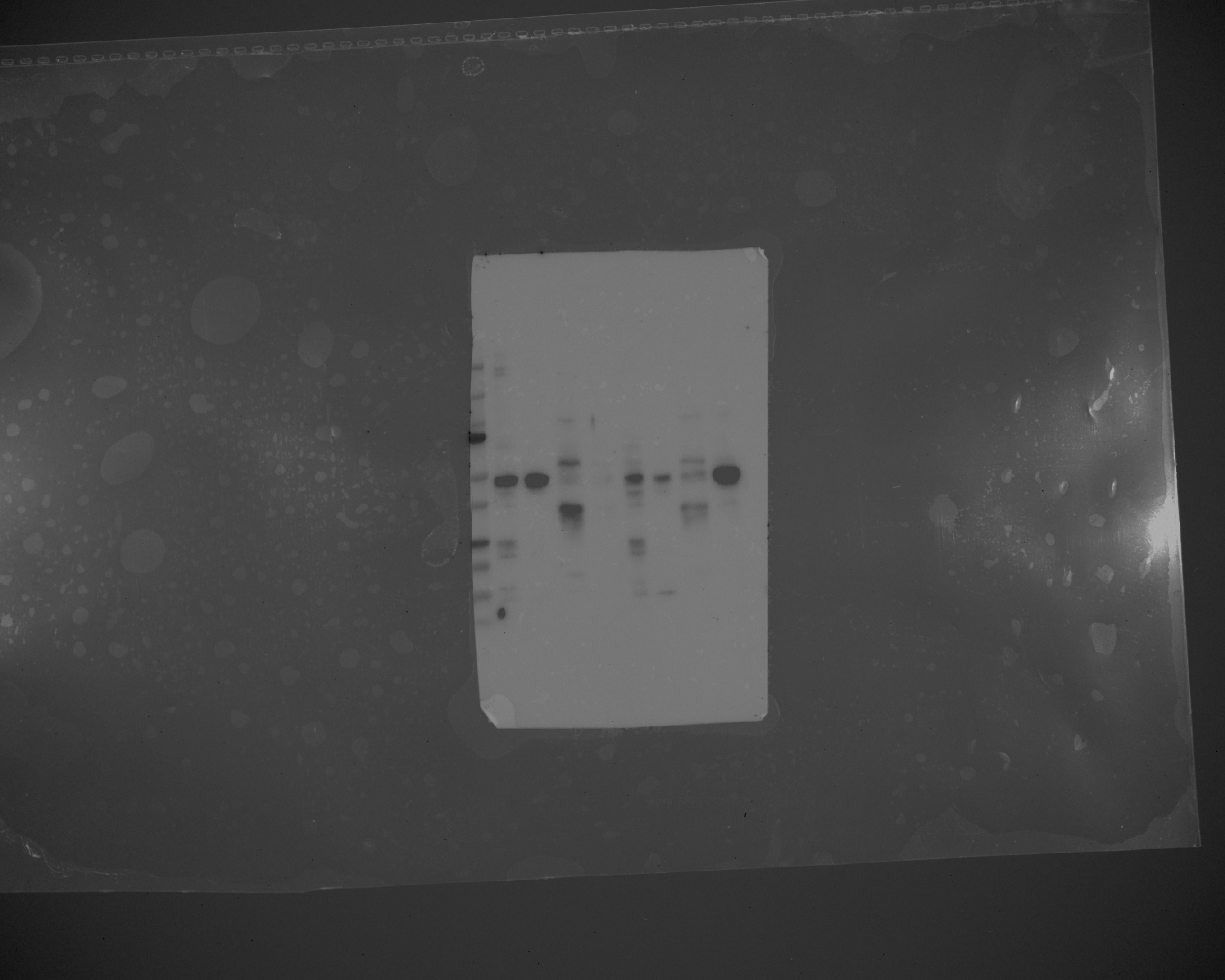

Supplement: Supplementary file 7 — Source Data Fig. 5 [file 44319_2023_27_MOESM7_ESM.zip › Figure 5/5J/Western AGAL.tif]

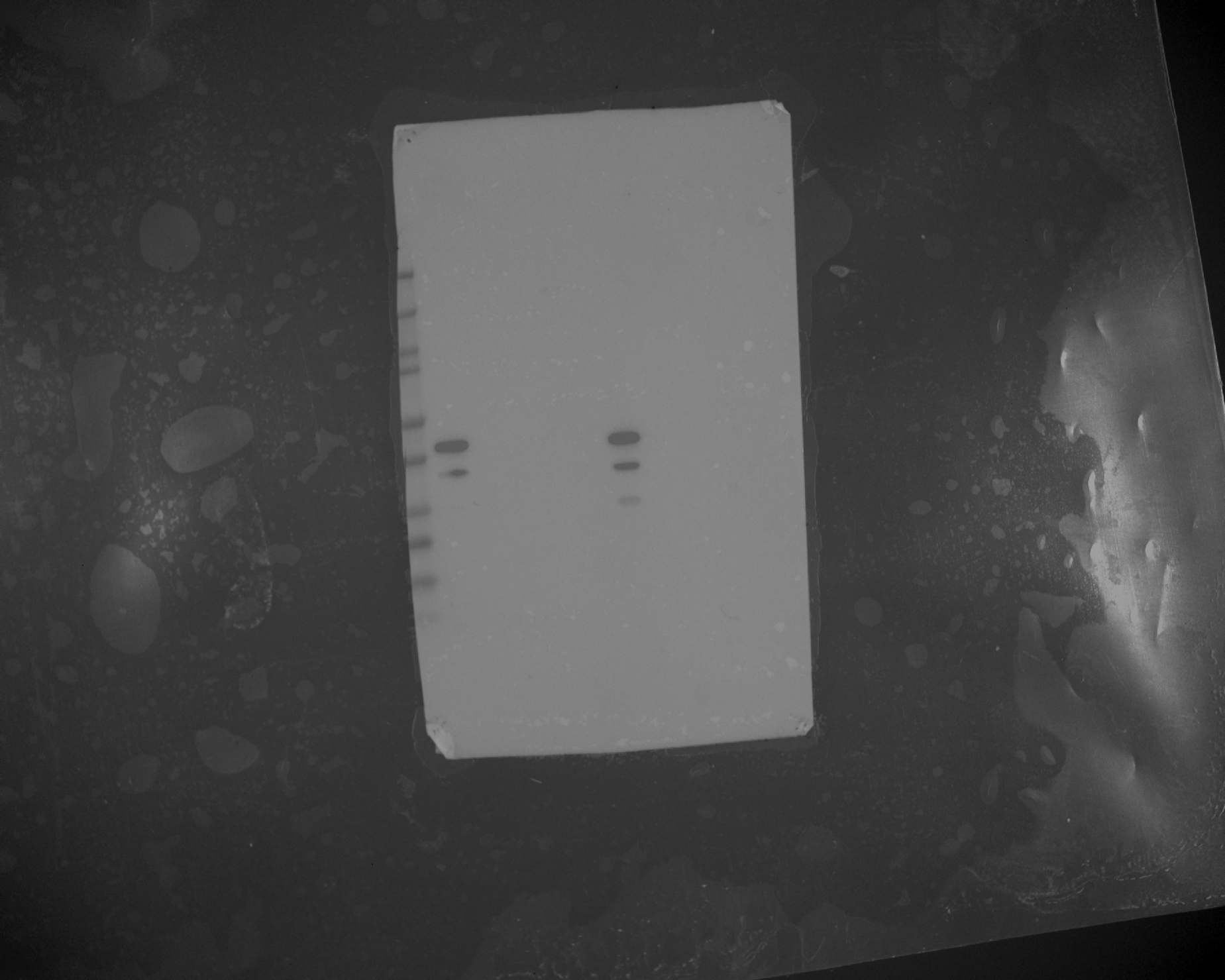

Supplement: Supplementary file 7 — Source Data Fig. 5 [file 44319_2023_27_MOESM7_ESM.zip › Figure 5/5J/Western UBXN1.tif]

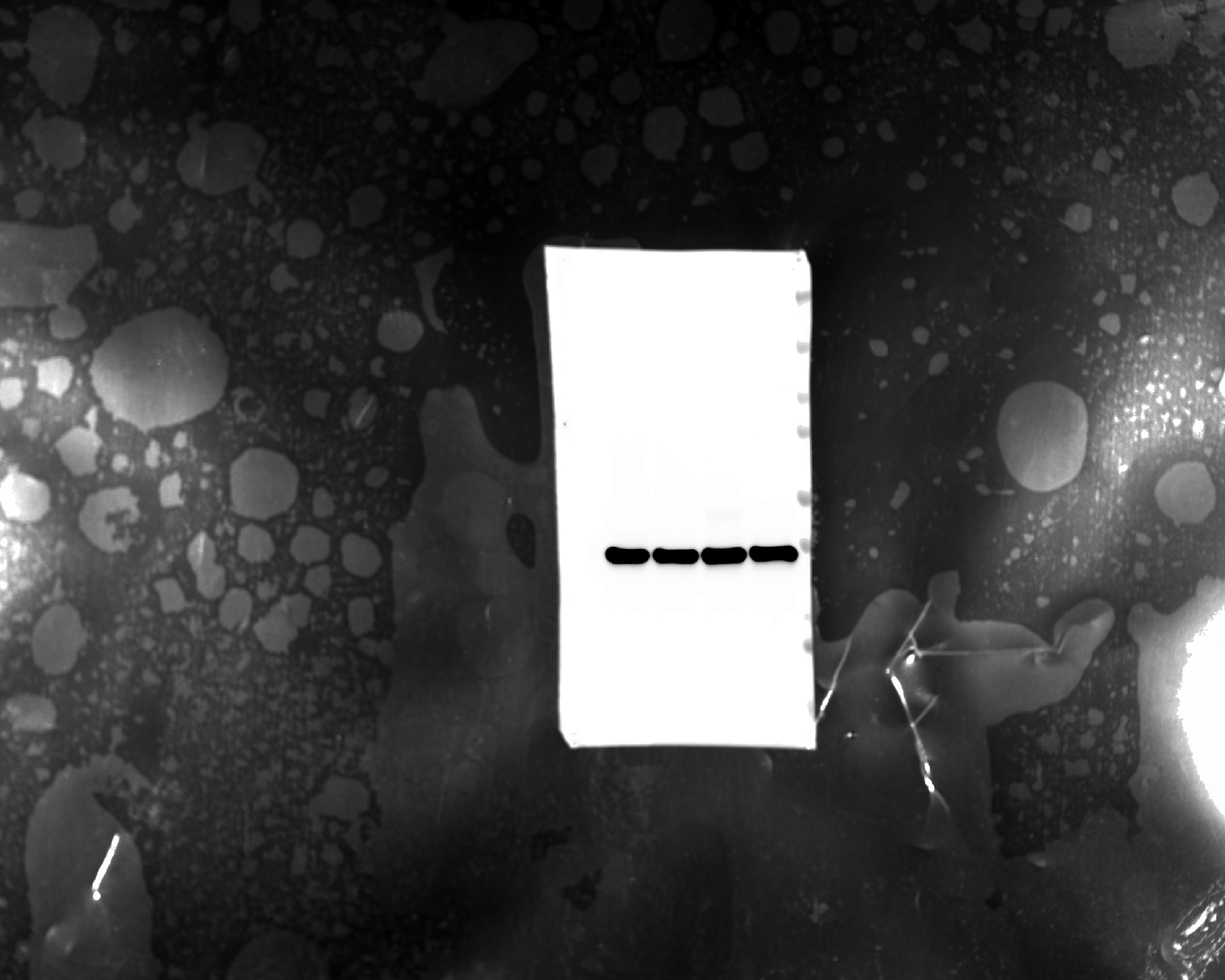

Supplement: Supplementary file 7 — Source Data Fig. 5 [file 44319_2023_27_MOESM7_ESM.zip › Figure 5/5C/western Bactin.tif]

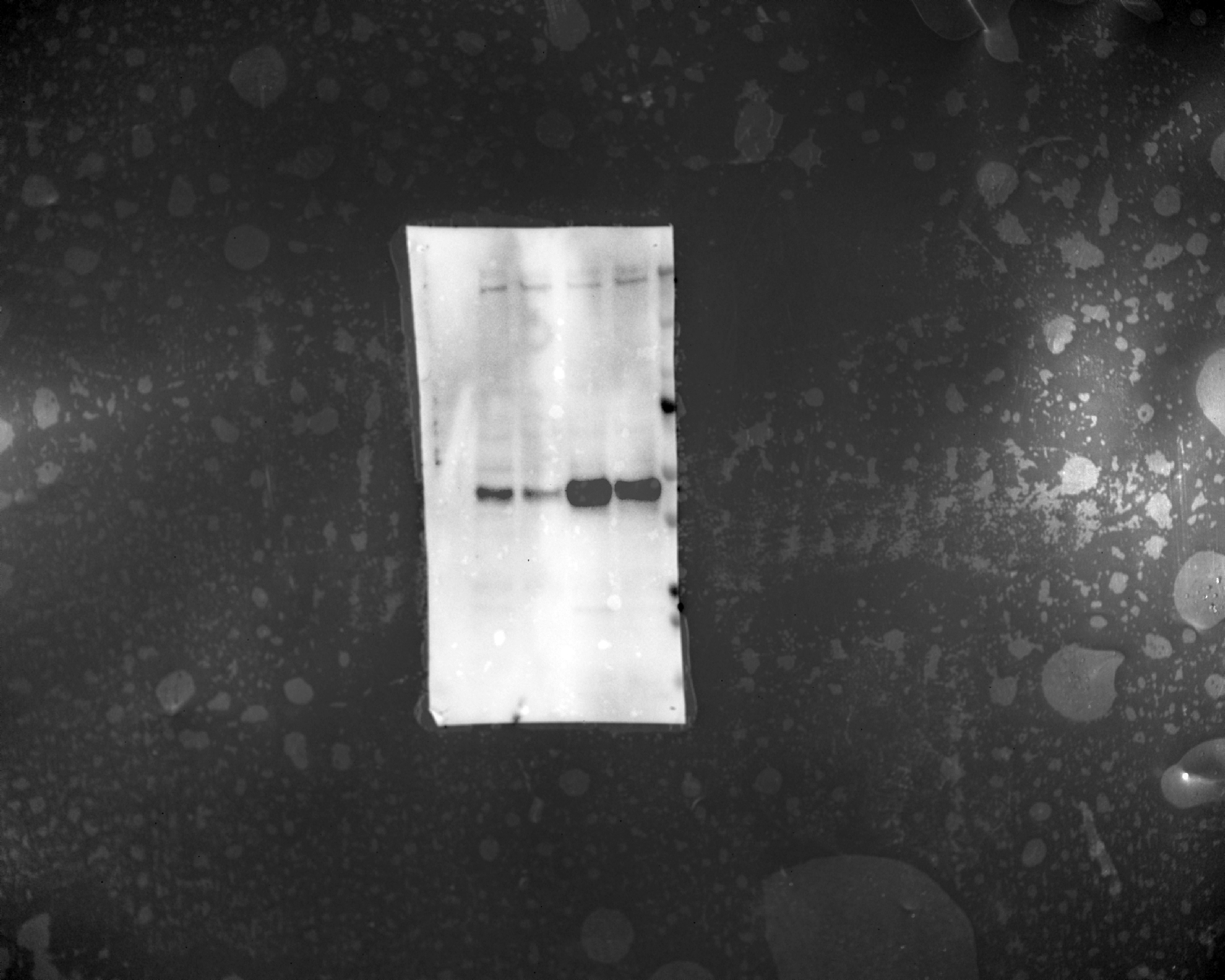

Supplement: Supplementary file 7 — Source Data Fig. 5 [file 44319_2023_27_MOESM7_ESM.zip › Figure 5/5C/Western AGAL.tif]

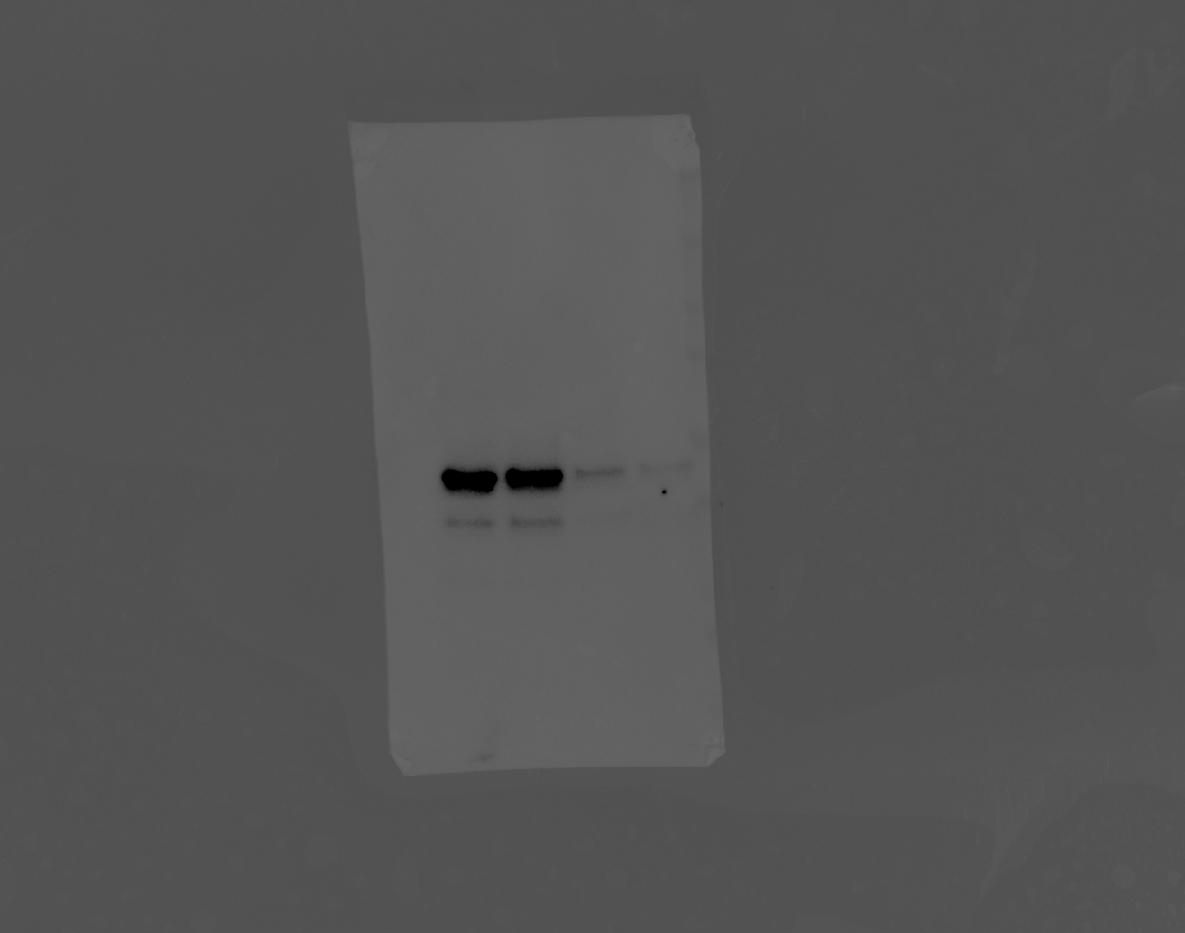

Supplement: Supplementary file 7 — Source Data Fig. 5 [file 44319_2023_27_MOESM7_ESM.zip › Figure 5/5C/western UBXN1.tif]

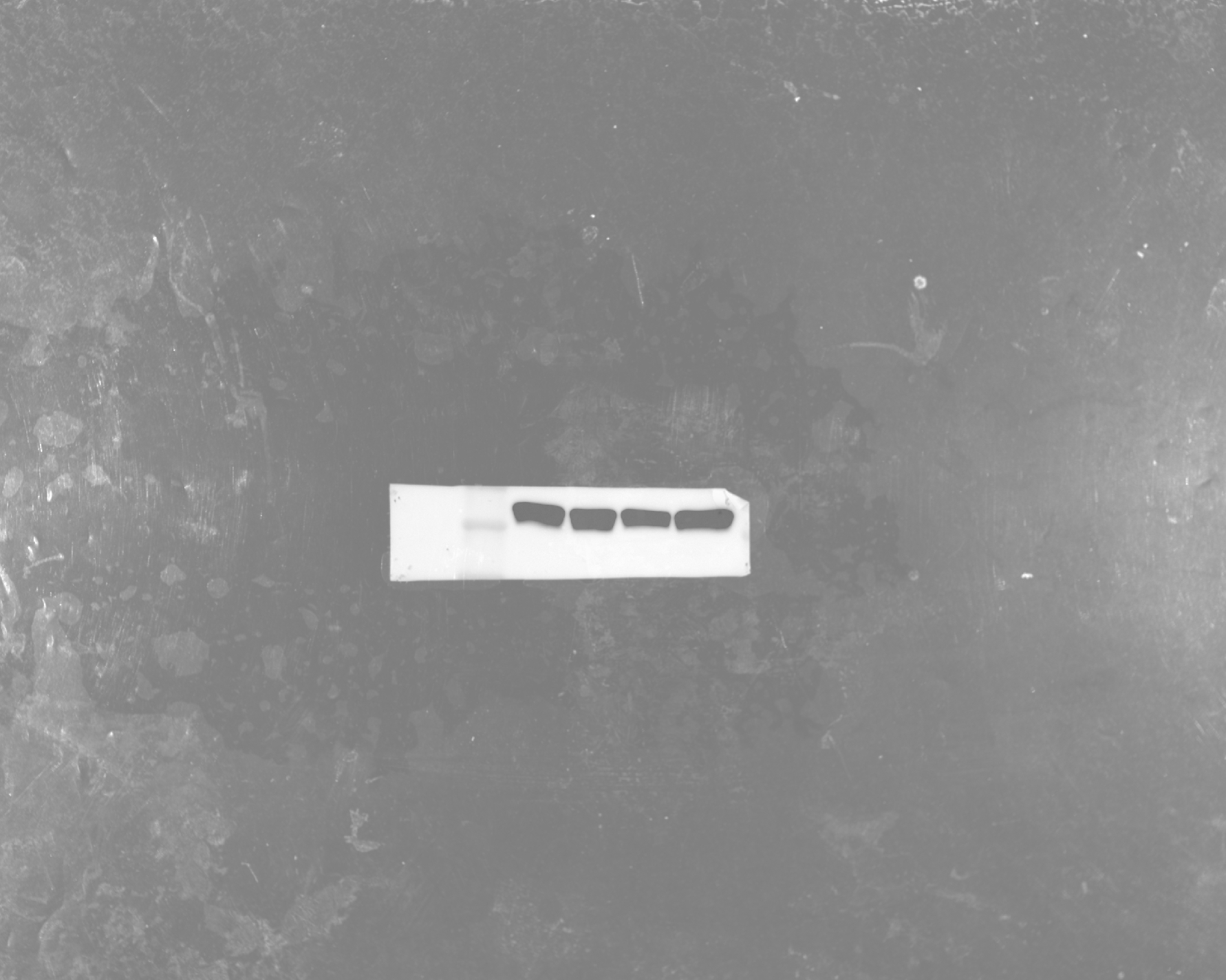

Supplement: Supplementary file 7 — Source Data Fig. 5 [file 44319_2023_27_MOESM7_ESM.zip › Figure 5/5D/western actin.tif]

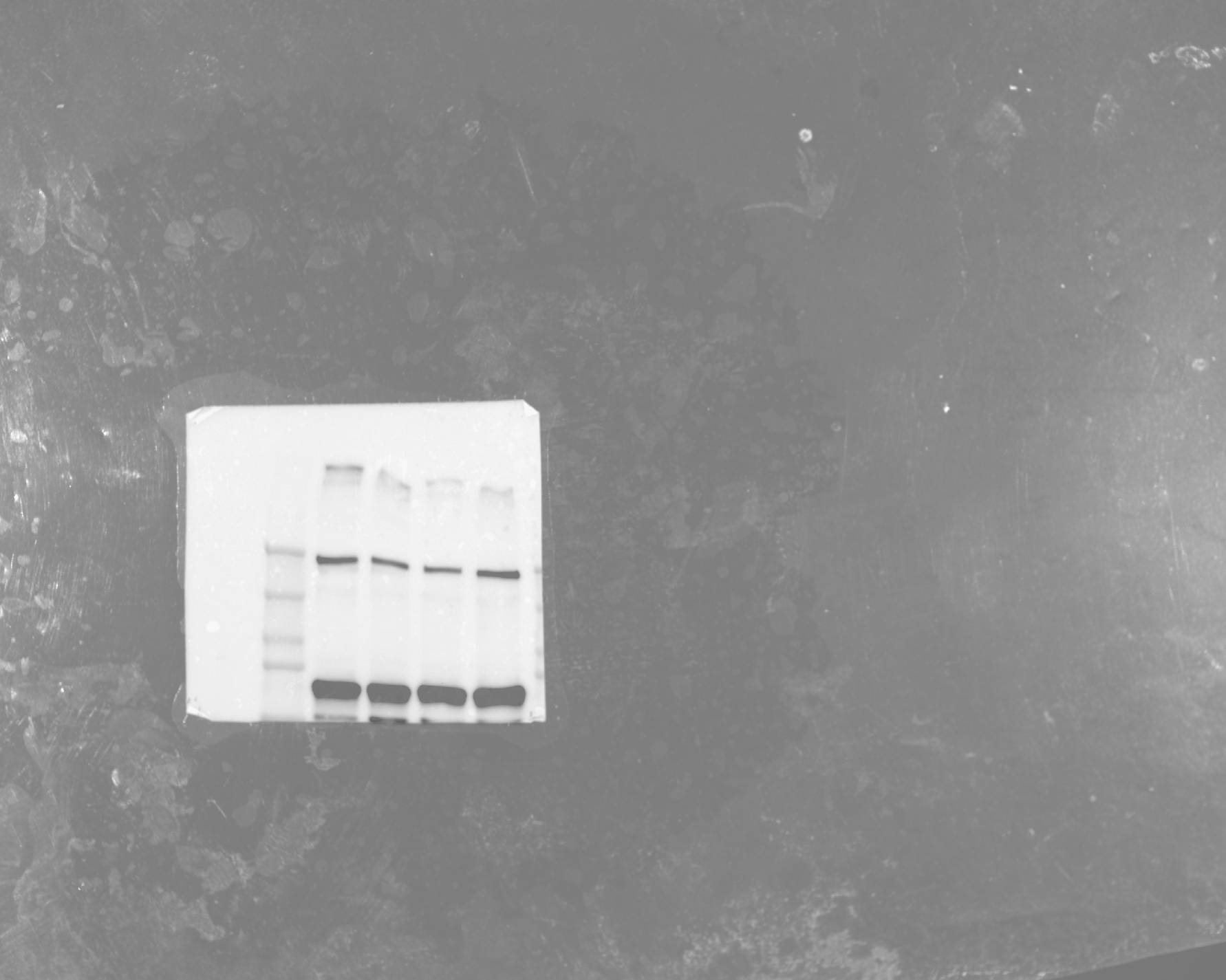

Supplement: Supplementary file 7 — Source Data Fig. 5 [file 44319_2023_27_MOESM7_ESM.zip › Figure 5/5D/western G3BP1.tif]

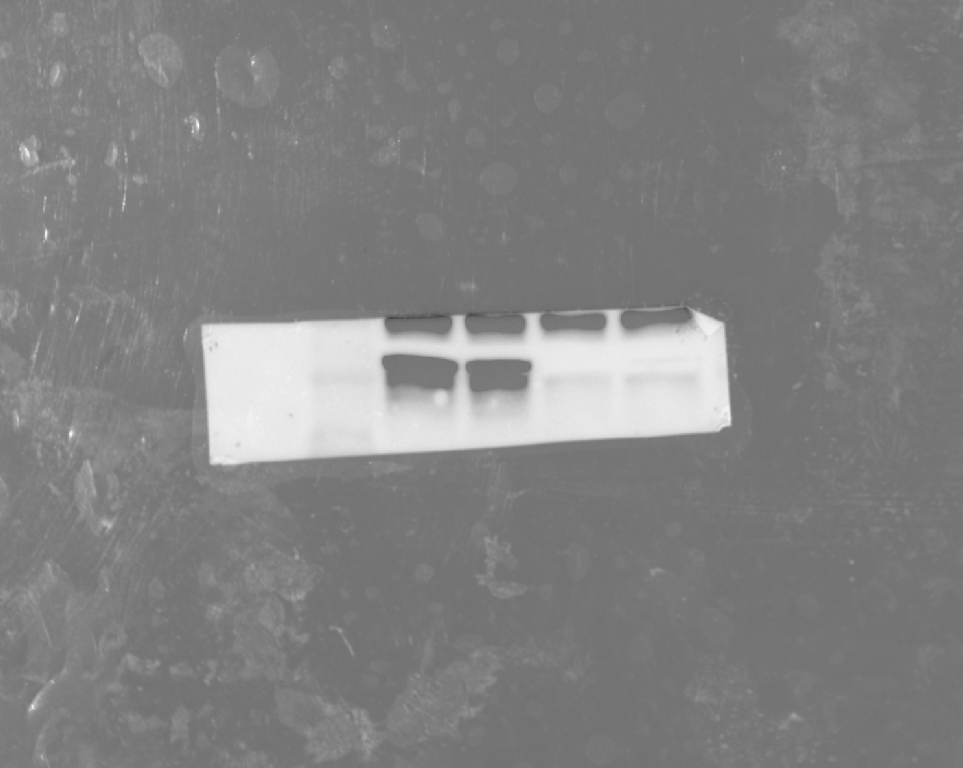

Supplement: Supplementary file 7 — Source Data Fig. 5 [file 44319_2023_27_MOESM7_ESM.zip › Figure 5/5D/western UBXN1.tif]

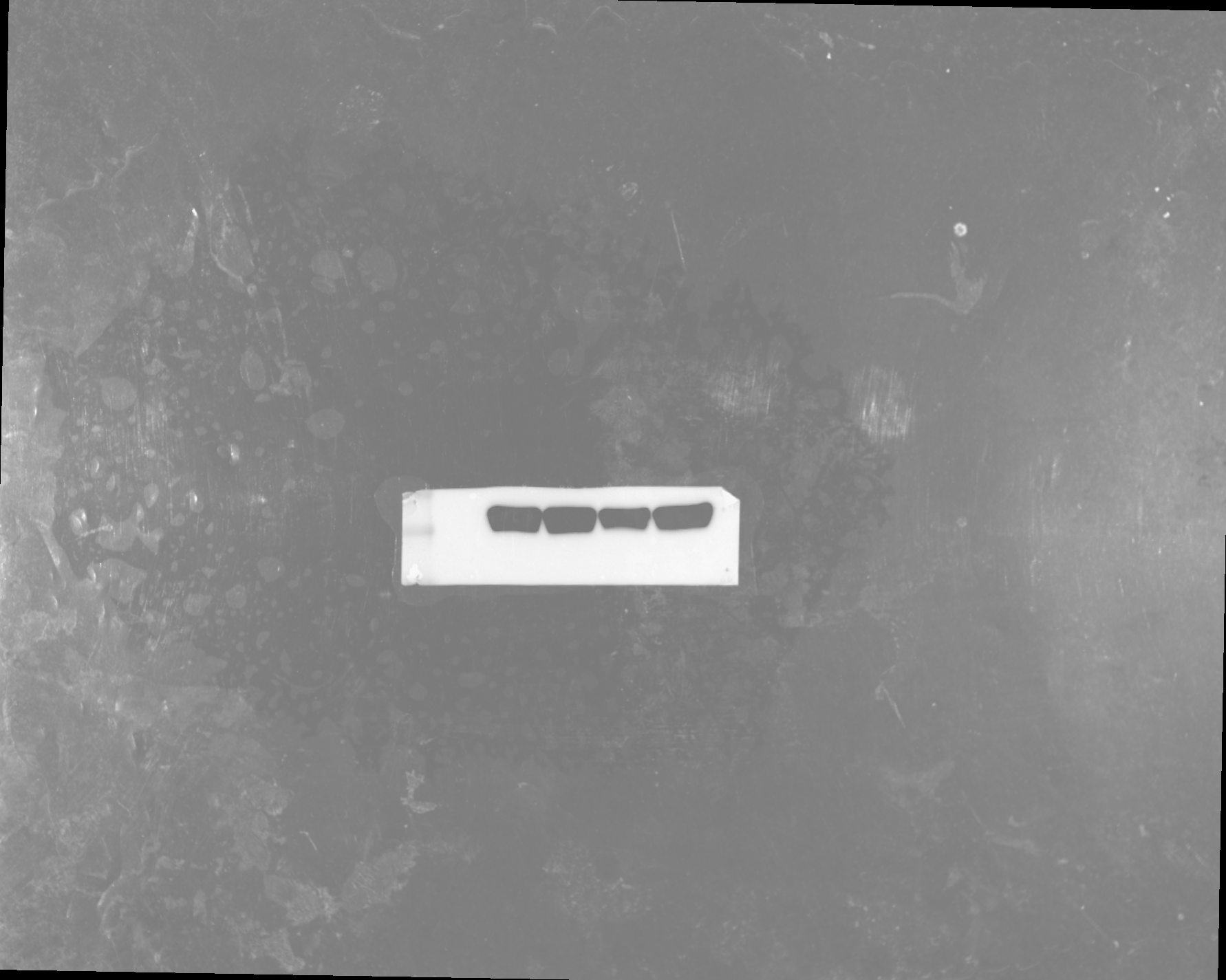

Supplement: Supplementary file 7 — Source Data Fig. 5 [file 44319_2023_27_MOESM7_ESM.zip › Figure 5/5E/western Bactin.tif]

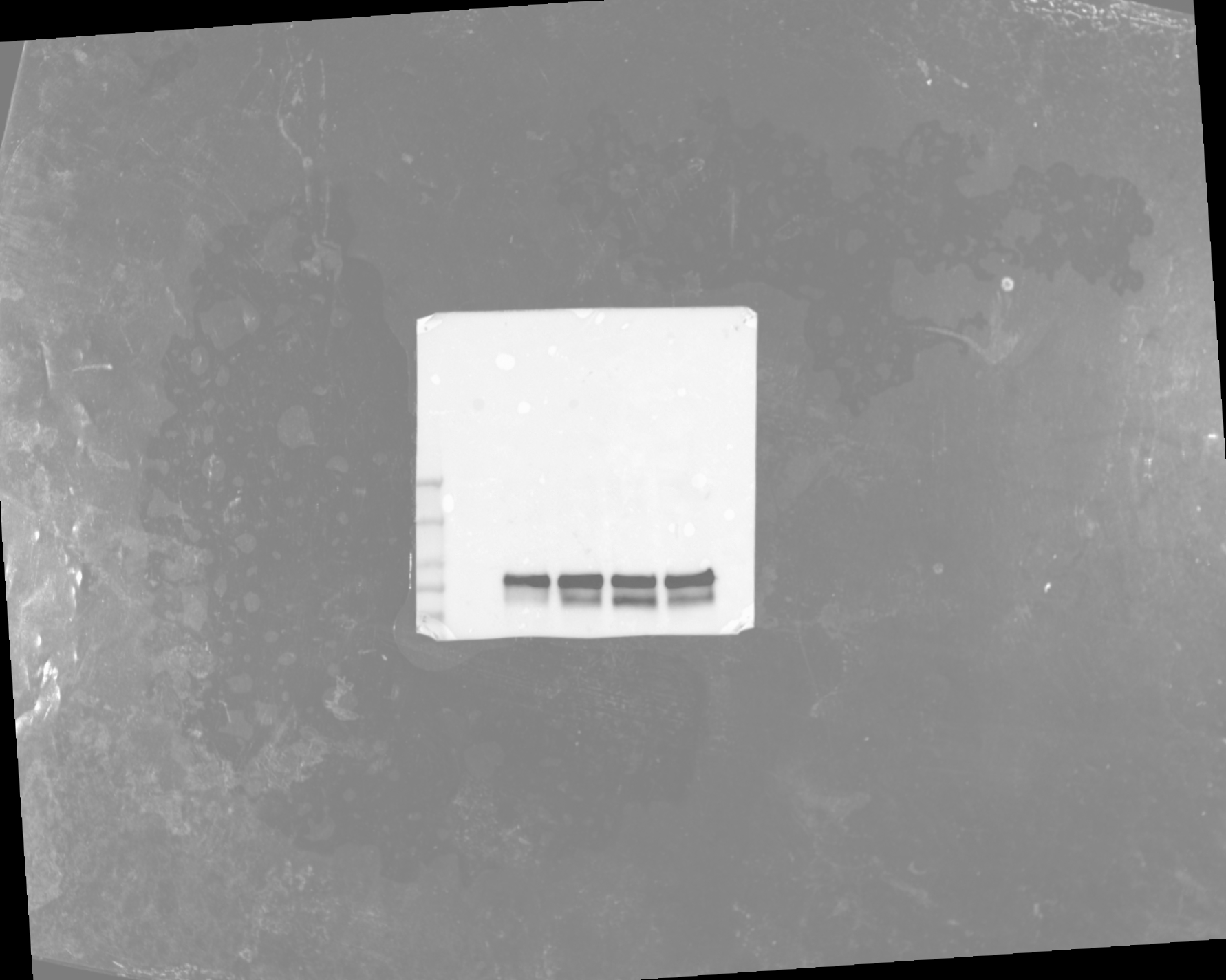

Supplement: Supplementary file 7 — Source Data Fig. 5 [file 44319_2023_27_MOESM7_ESM.zip › Figure 5/5E/western HSP90.tif]

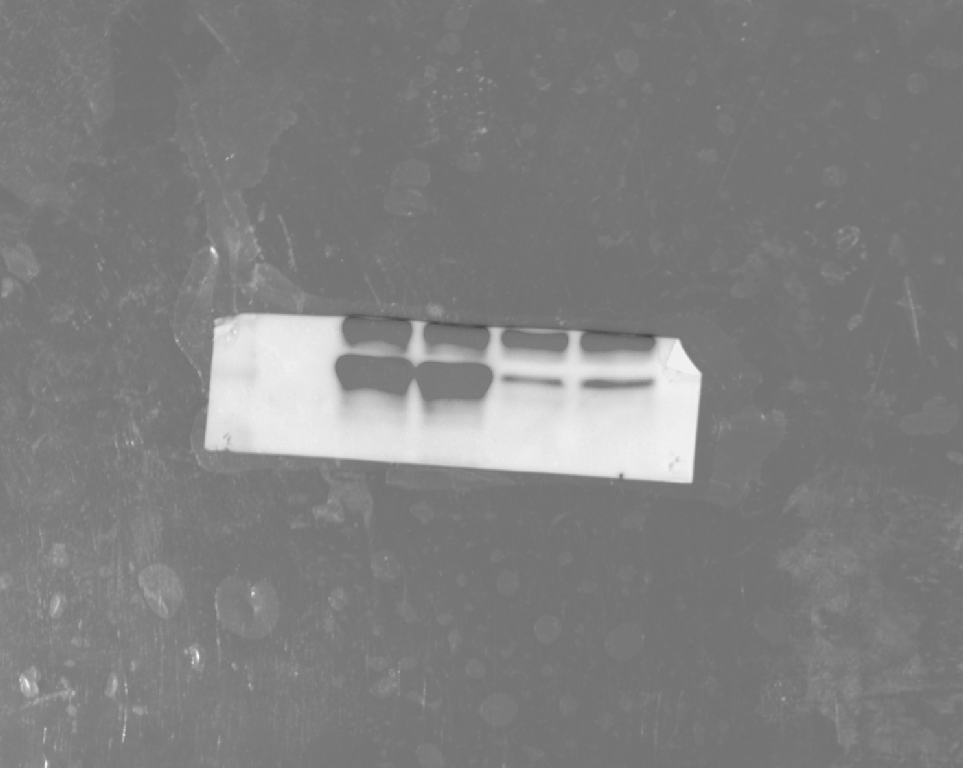

Supplement: Supplementary file 7 — Source Data Fig. 5 [file 44319_2023_27_MOESM7_ESM.zip › Figure 5/5E/western UBXN1.tif]

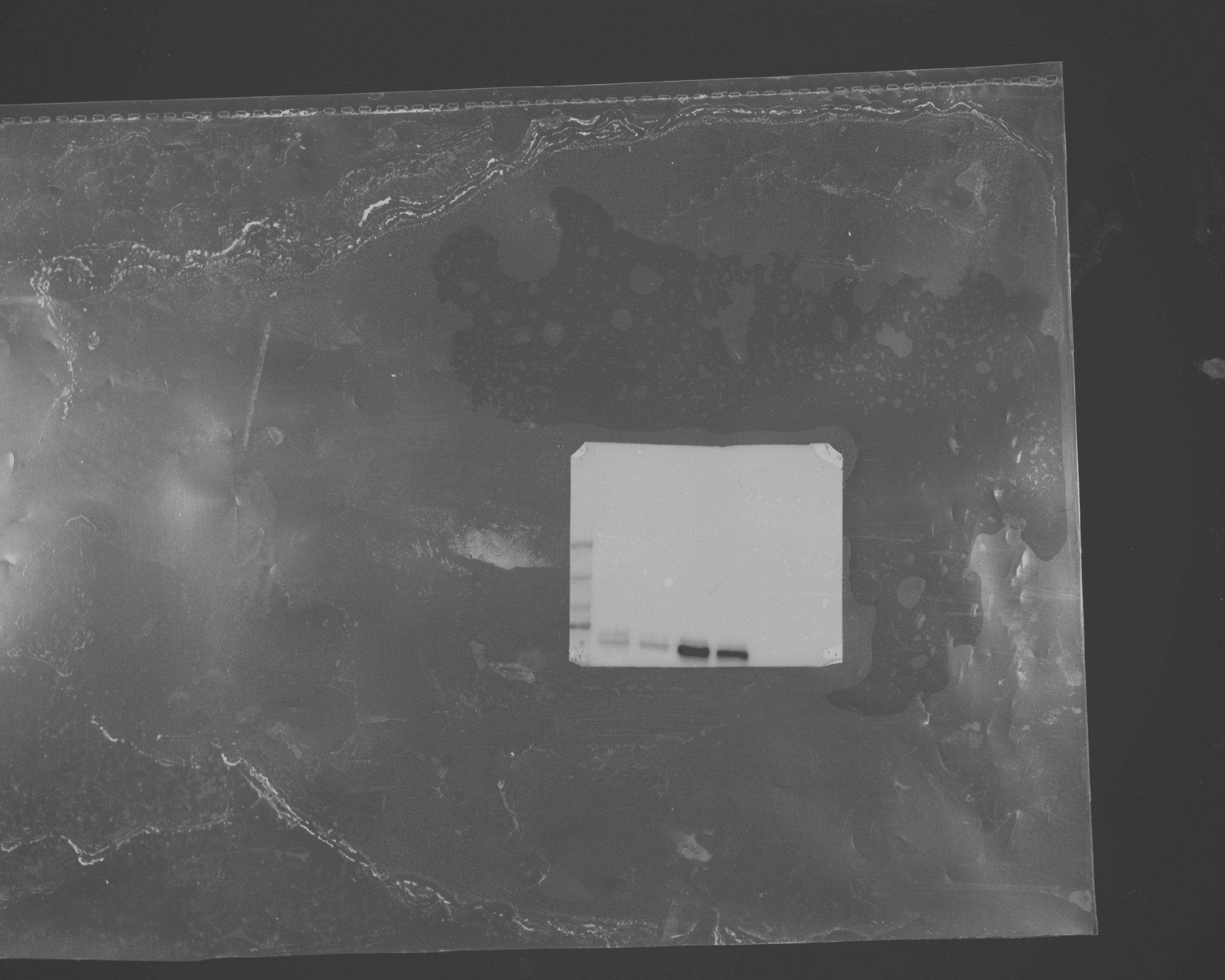

Supplement: Supplementary file 7 — Source Data Fig. 5 [file 44319_2023_27_MOESM7_ESM.zip › Figure 5/5B/western ALPP2.tif]

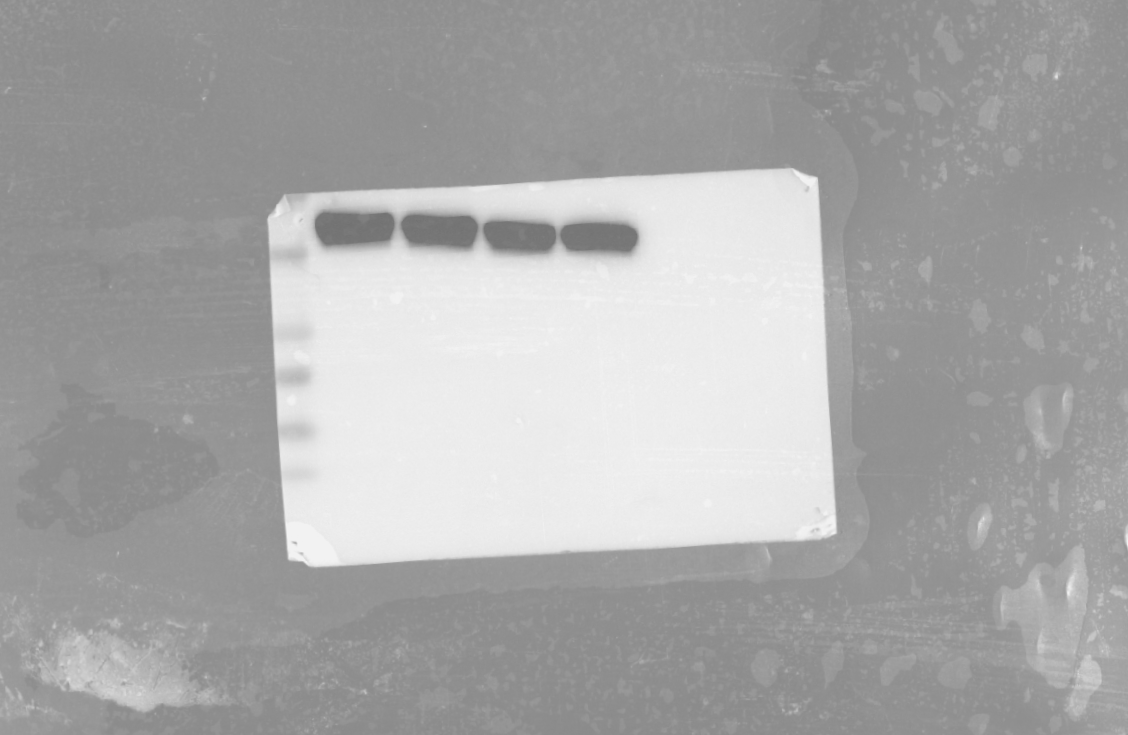

Supplement: Supplementary file 7 — Source Data Fig. 5 [file 44319_2023_27_MOESM7_ESM.zip › Figure 5/5B/western Bactin.tif]

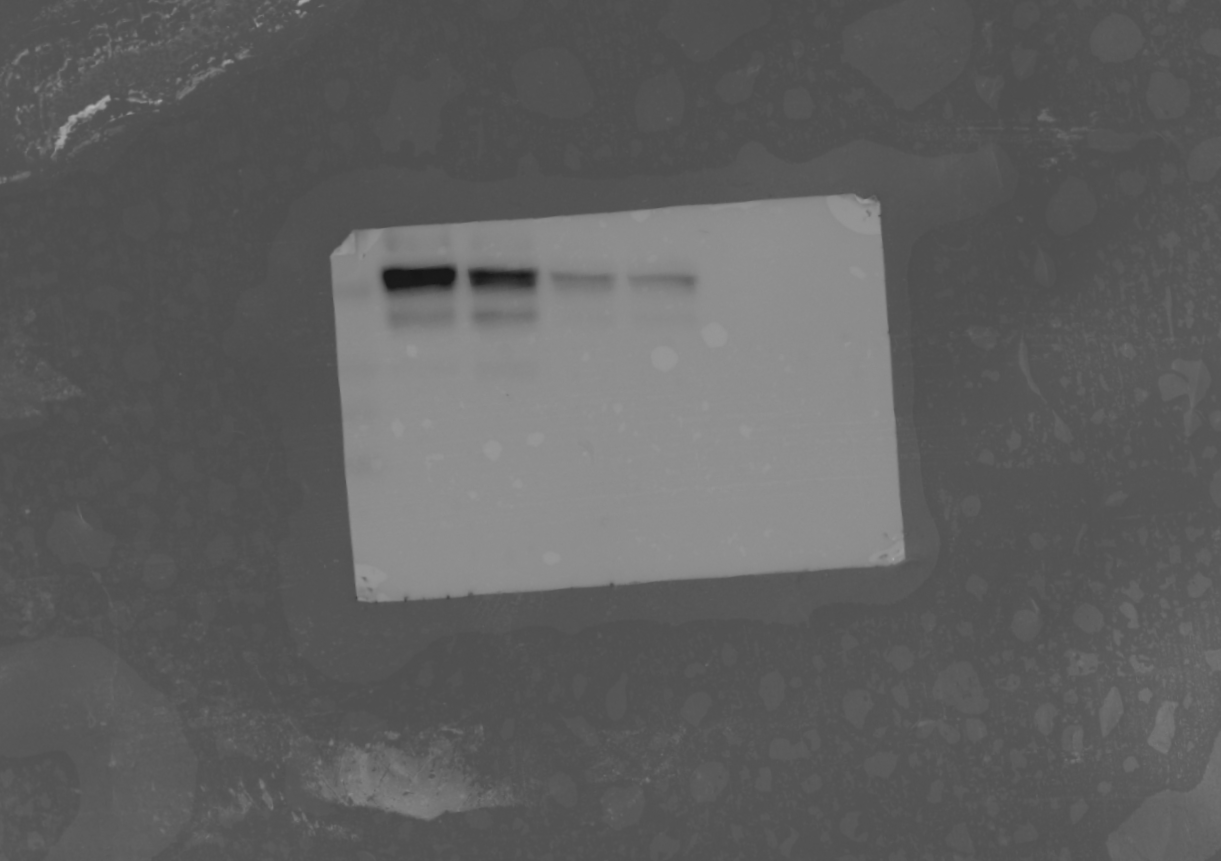

Supplement: Supplementary file 7 — Source Data Fig. 5 [file 44319_2023_27_MOESM7_ESM.zip › Figure 5/5B/western UBXN1.tif]

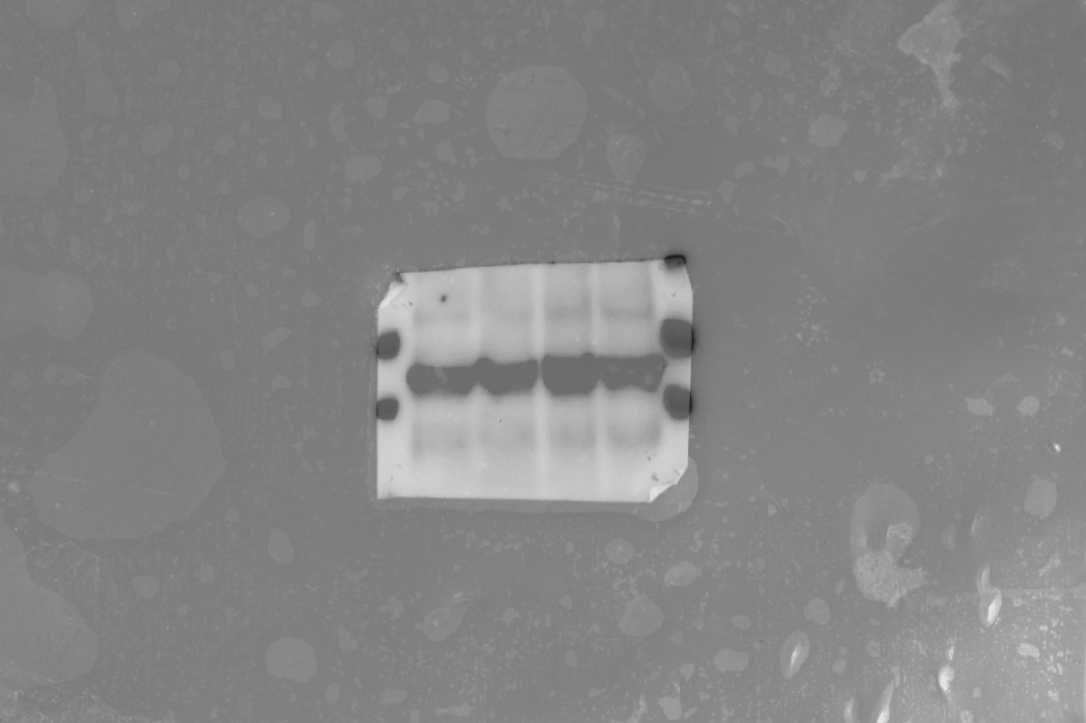

Supplement: Supplementary file 8 — Source Data Fig. 6 [file 44319_2023_27_MOESM8_ESM.zip › Figure 6/6A/western Bactin.tif]

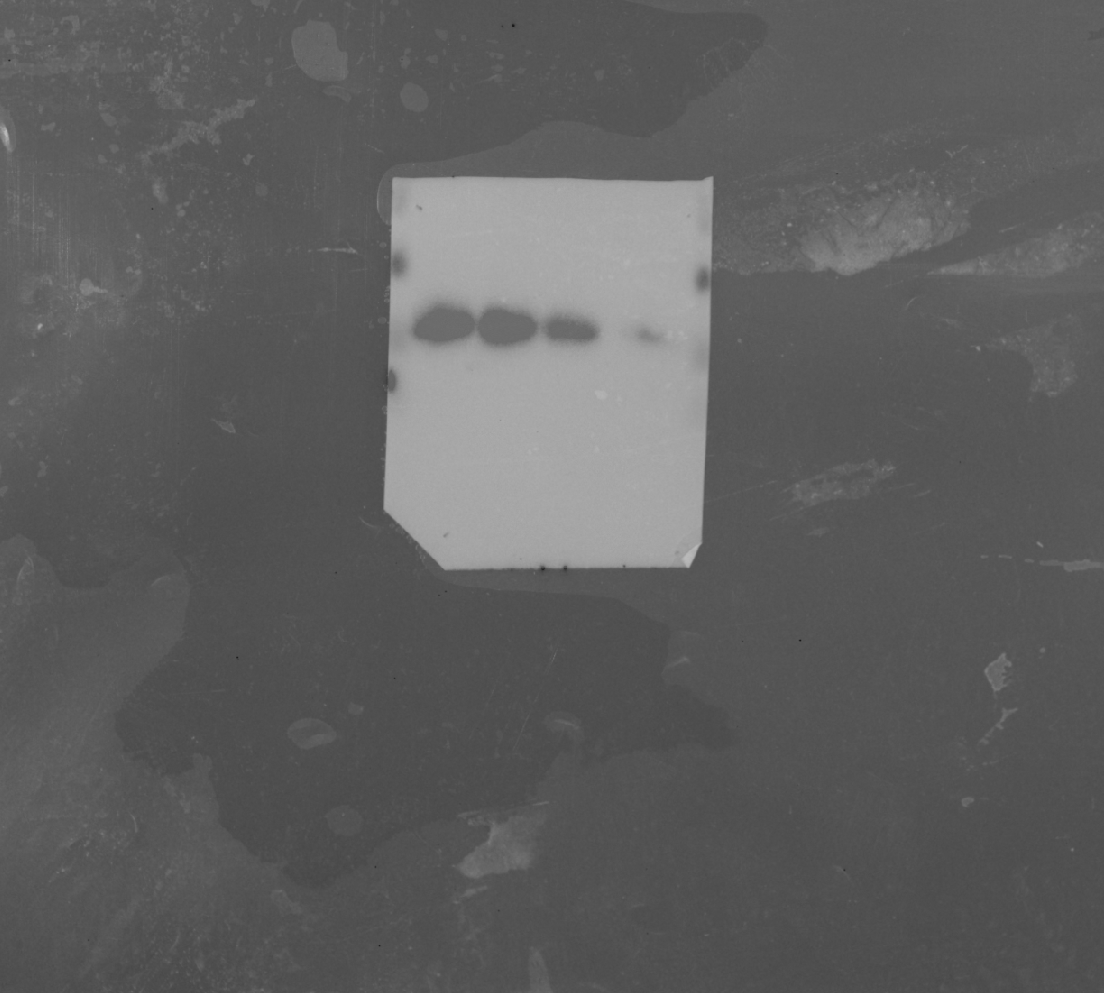

Supplement: Supplementary file 8 — Source Data Fig. 6 [file 44319_2023_27_MOESM8_ESM.zip › Figure 6/6A/western TOMM20.tif]

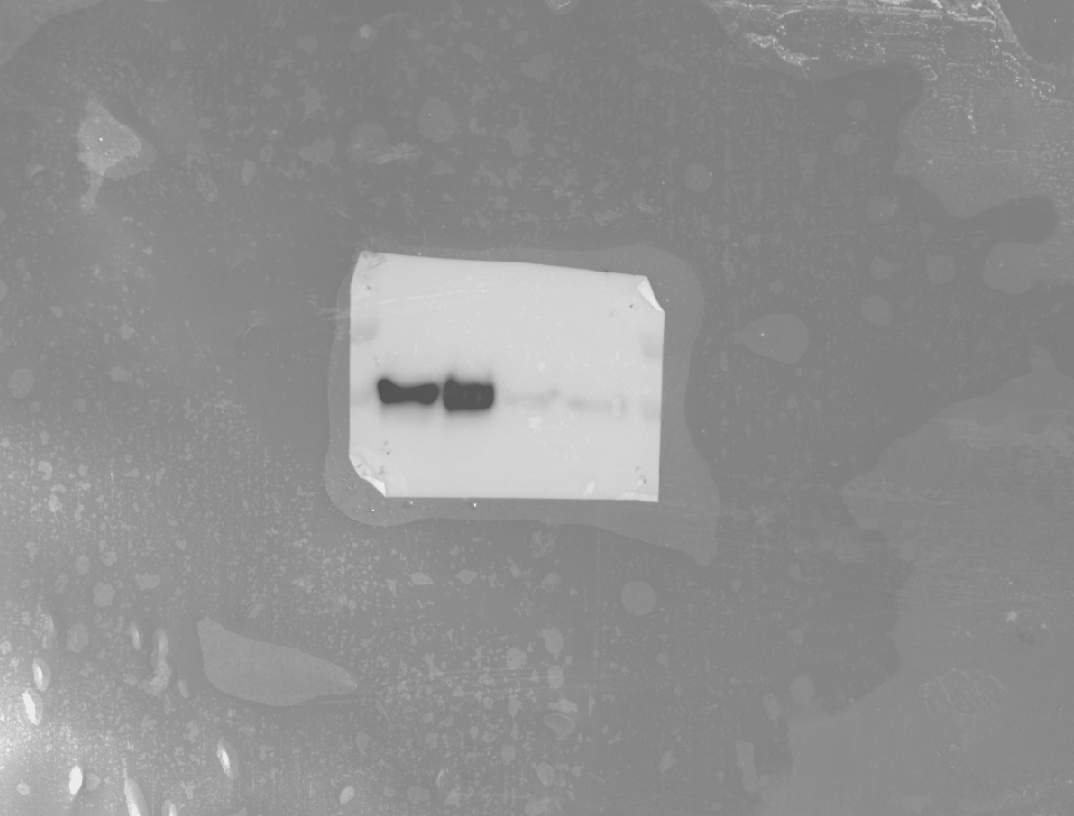

Supplement: Supplementary file 8 — Source Data Fig. 6 [file 44319_2023_27_MOESM8_ESM.zip › Figure 6/6A/western UBXN1.tif]

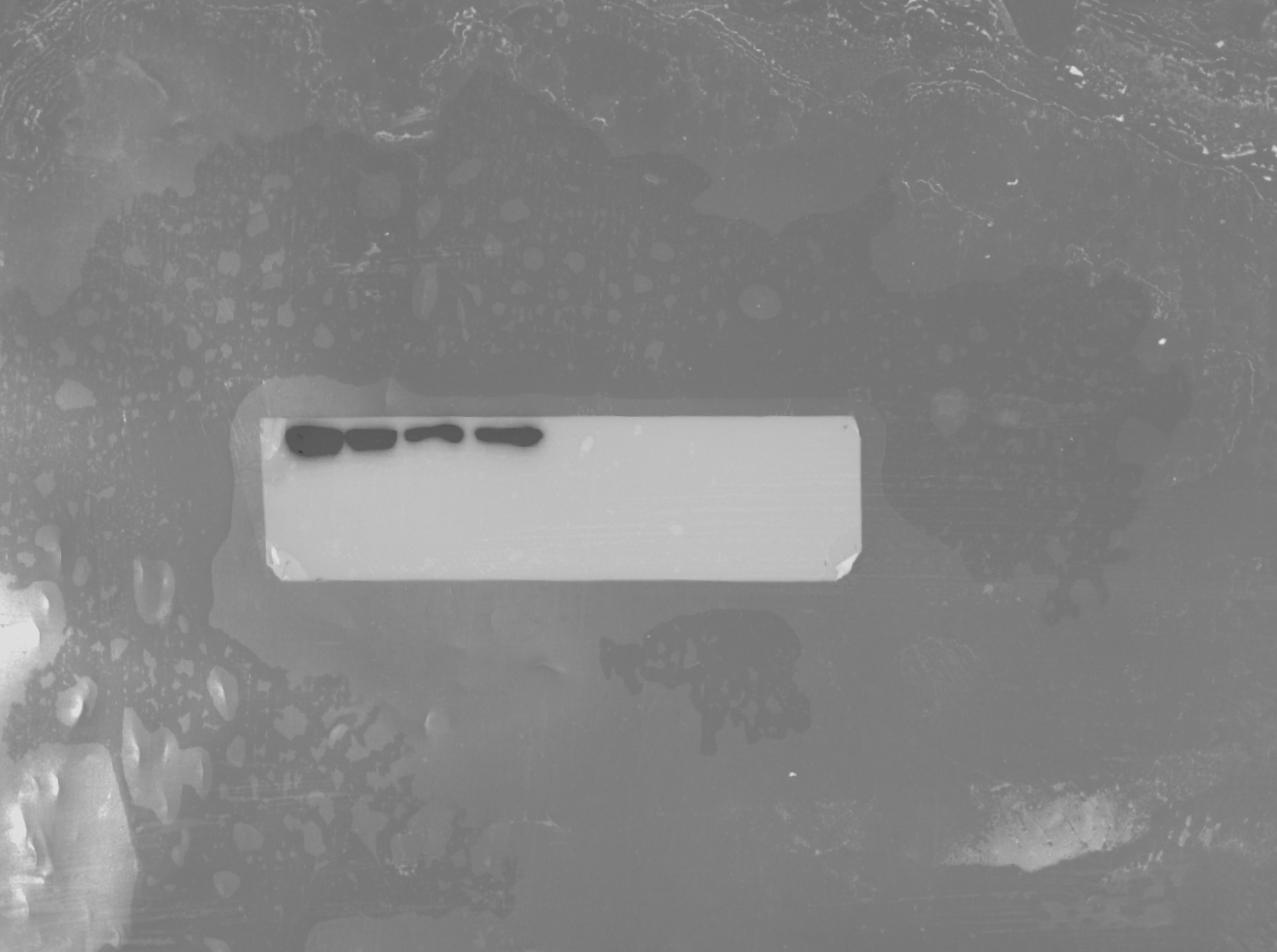

Supplement: Supplementary file 8 — Source Data Fig. 6 [file 44319_2023_27_MOESM8_ESM.zip › Figure 6/6B/western Bactin.tif]

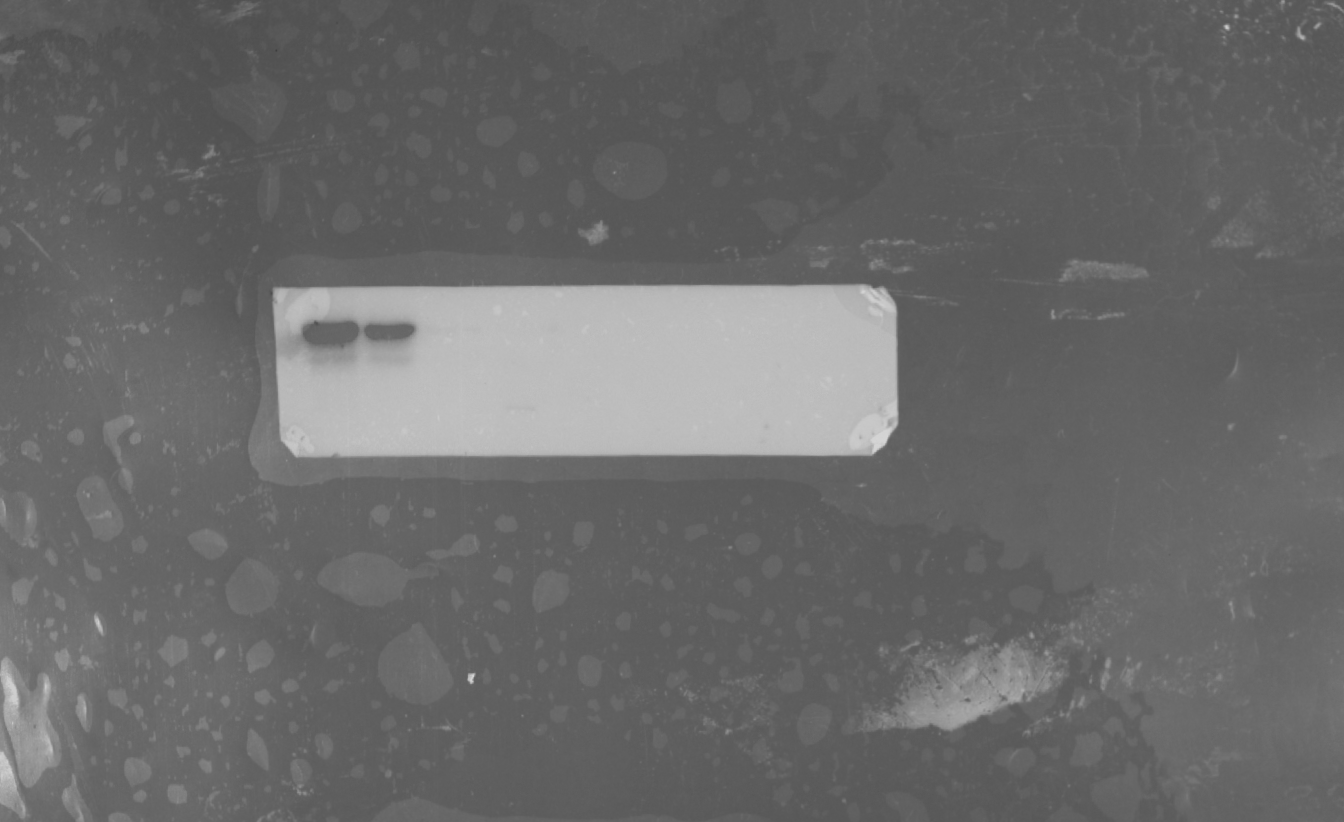

Supplement: Supplementary file 8 — Source Data Fig. 6 [file 44319_2023_27_MOESM8_ESM.zip › Figure 6/6B/western UBXN1.tif]

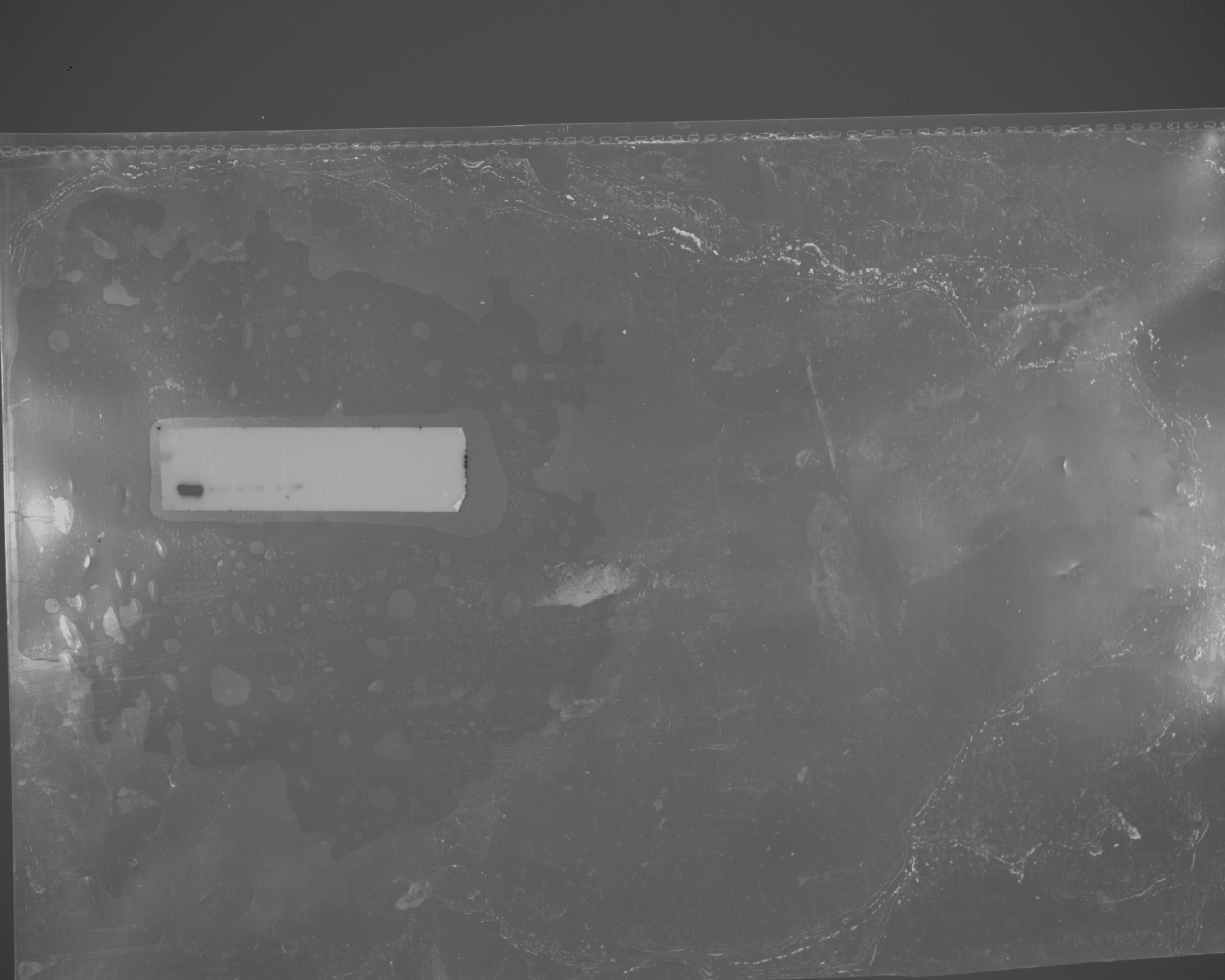

Supplement: Supplementary file 8 — Source Data Fig. 6 [file 44319_2023_27_MOESM8_ESM.zip › Figure 6/6B/western cyc1.tif]

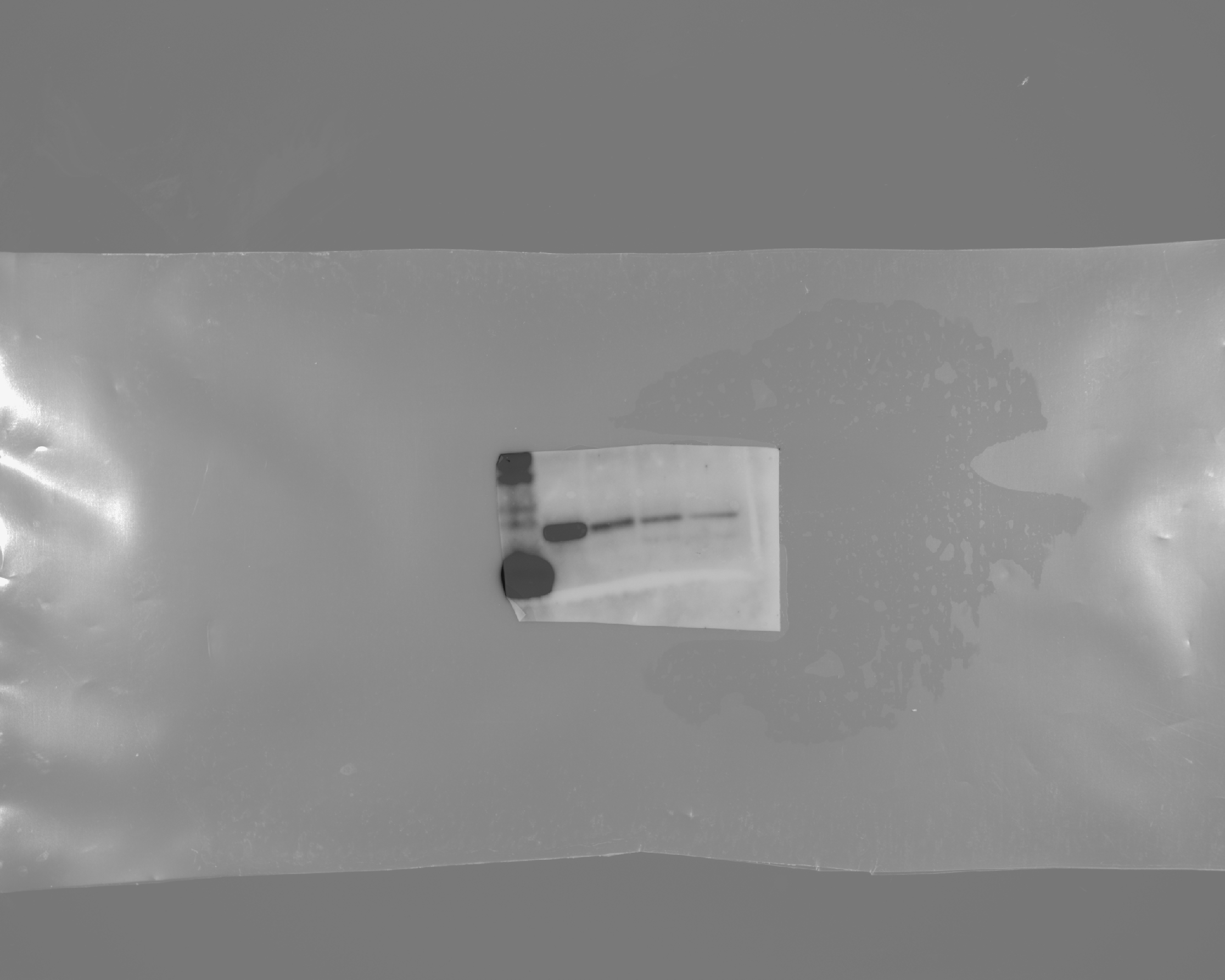

Supplement: Supplementary file 8 — Source Data Fig. 6 [file 44319_2023_27_MOESM8_ESM.zip › Figure 6/6D/western TIMM17A.jpg]

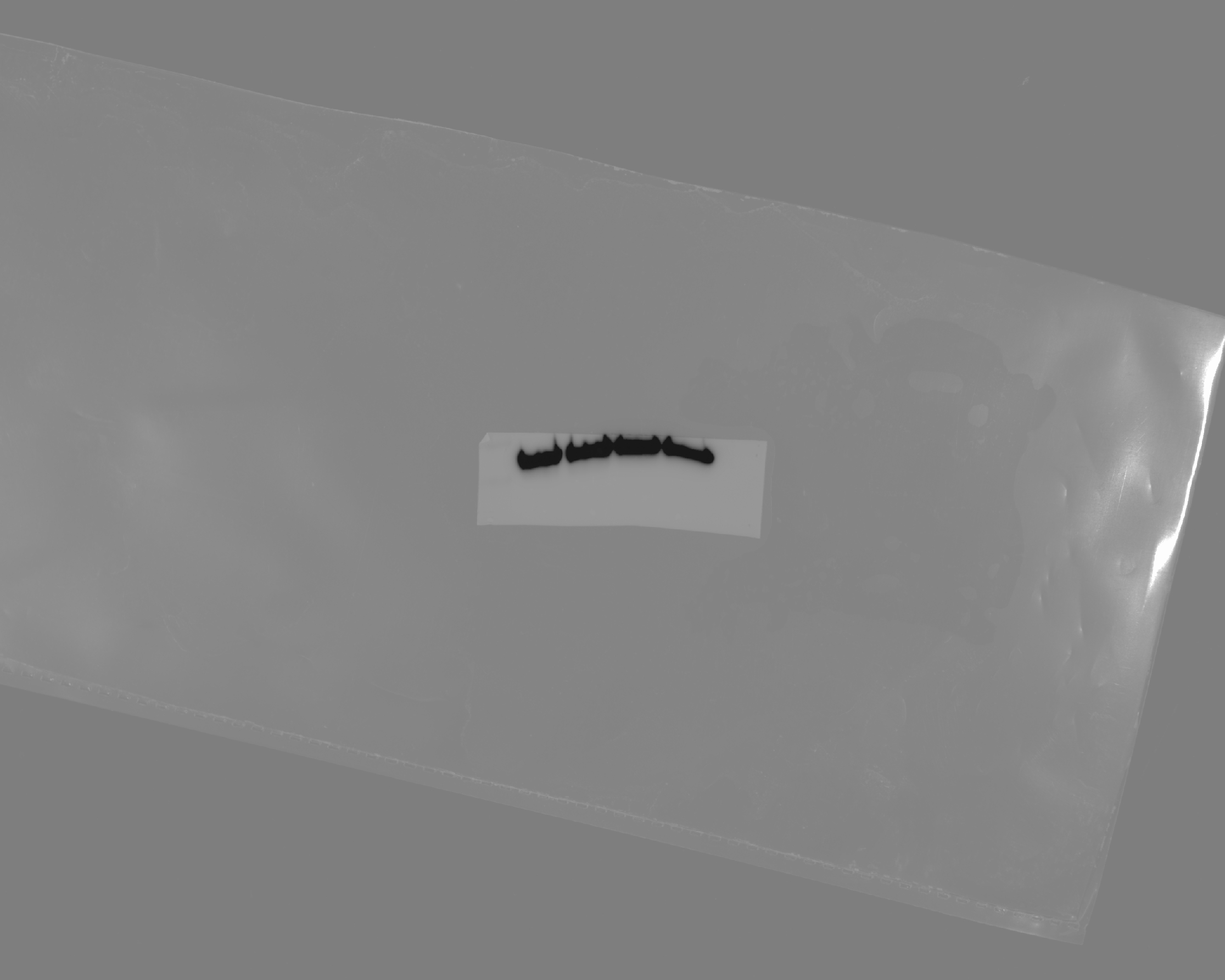

Supplement: Supplementary file 8 — Source Data Fig. 6 [file 44319_2023_27_MOESM8_ESM.zip › Figure 6/6D/western Bactin.tif]

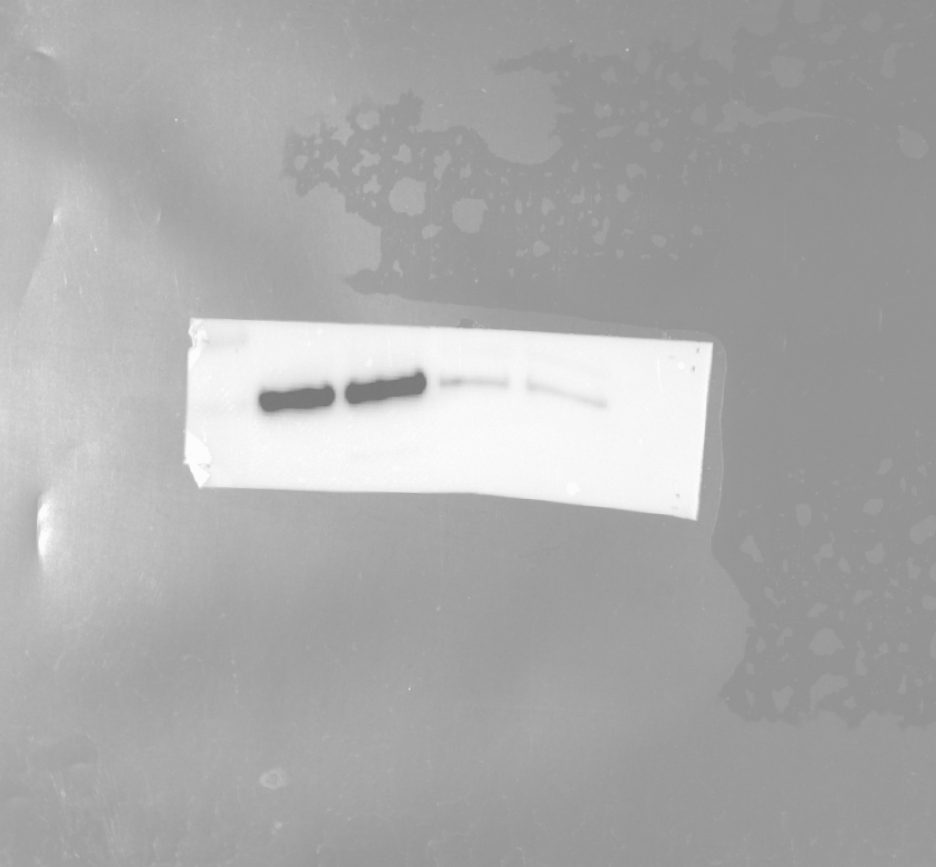

Supplement: Supplementary file 8 — Source Data Fig. 6 [file 44319_2023_27_MOESM8_ESM.zip › Figure 6/6D/western UBXN1.tif]

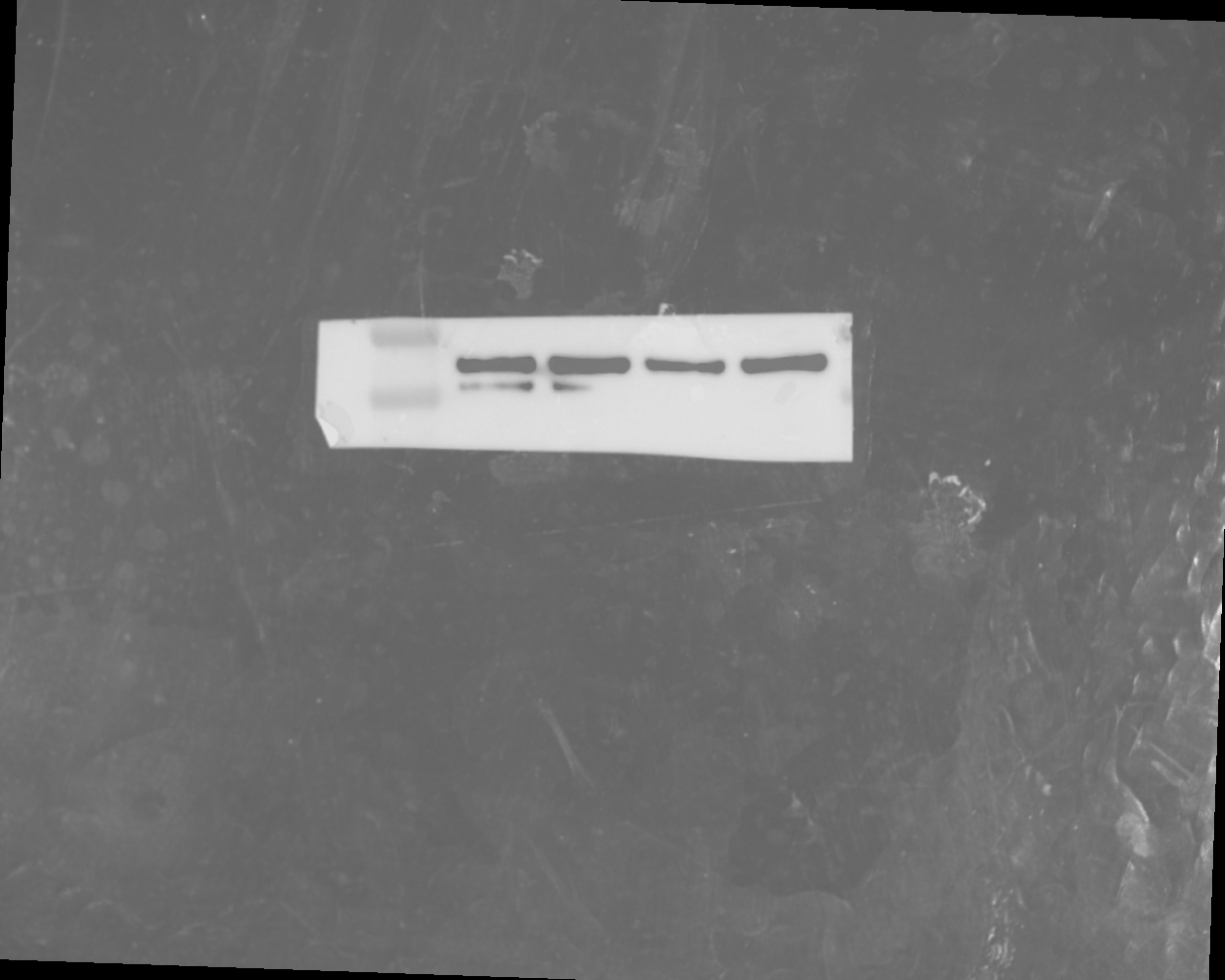

Supplement: Supplementary file 8 — Source Data Fig. 6 [file 44319_2023_27_MOESM8_ESM.zip › Figure 6/6C/western Bactin.tif]

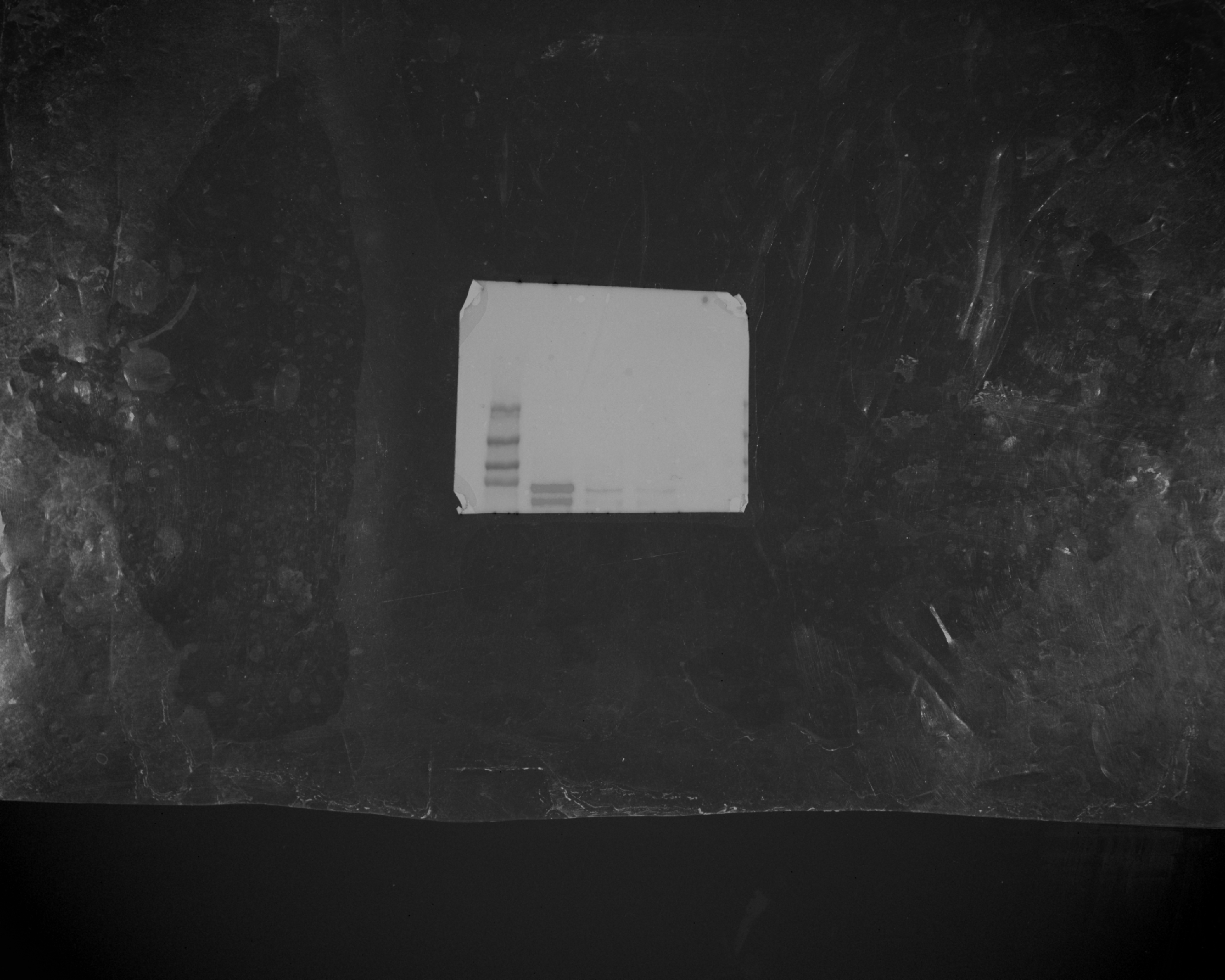

Supplement: Supplementary file 8 — Source Data Fig. 6 [file 44319_2023_27_MOESM8_ESM.zip › Figure 6/6C/western TOMM70.jpg]

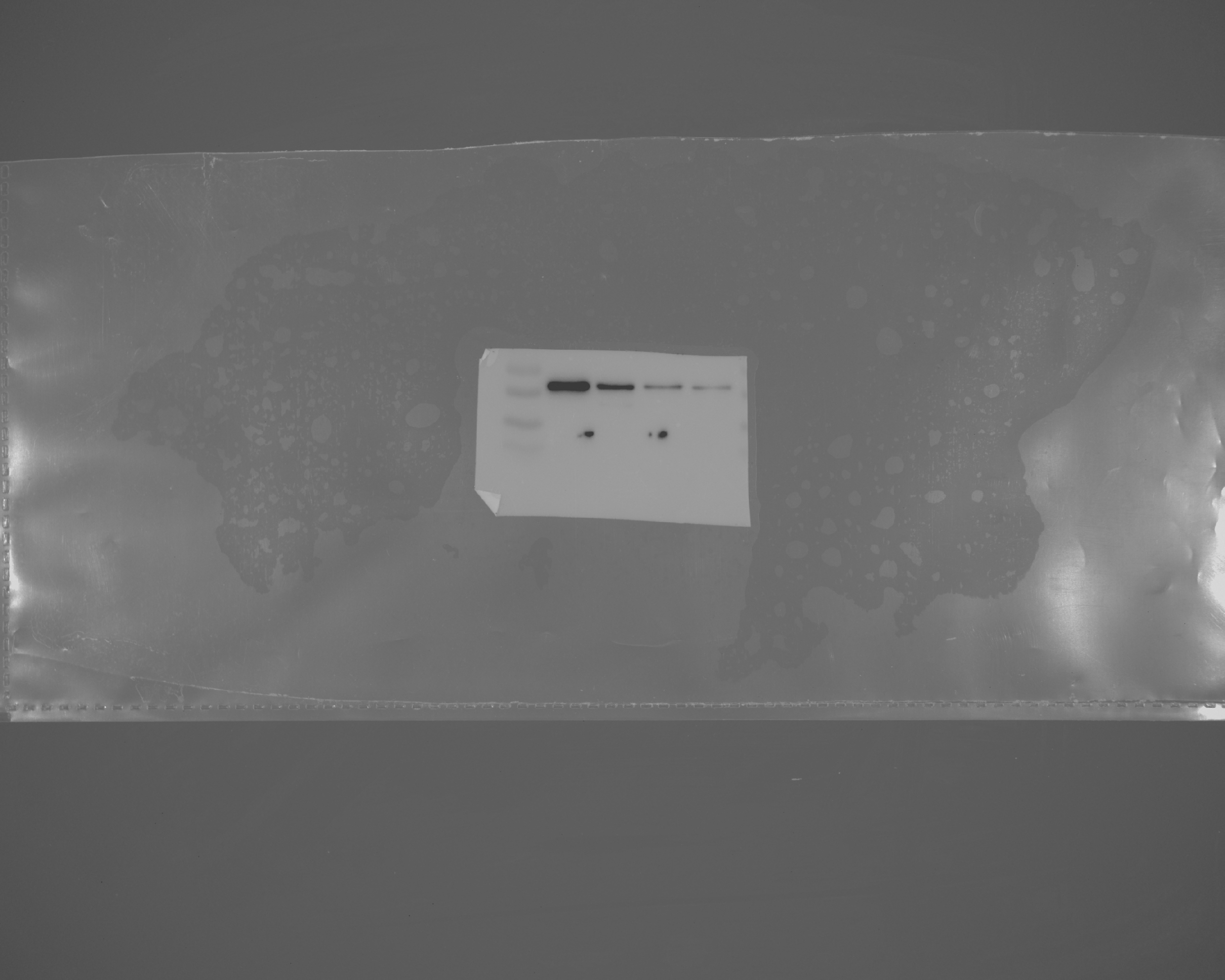

Supplement: Supplementary file 8 — Source Data Fig. 6 [file 44319_2023_27_MOESM8_ESM.zip › Figure 6/6C/western TIMM23.jpg]

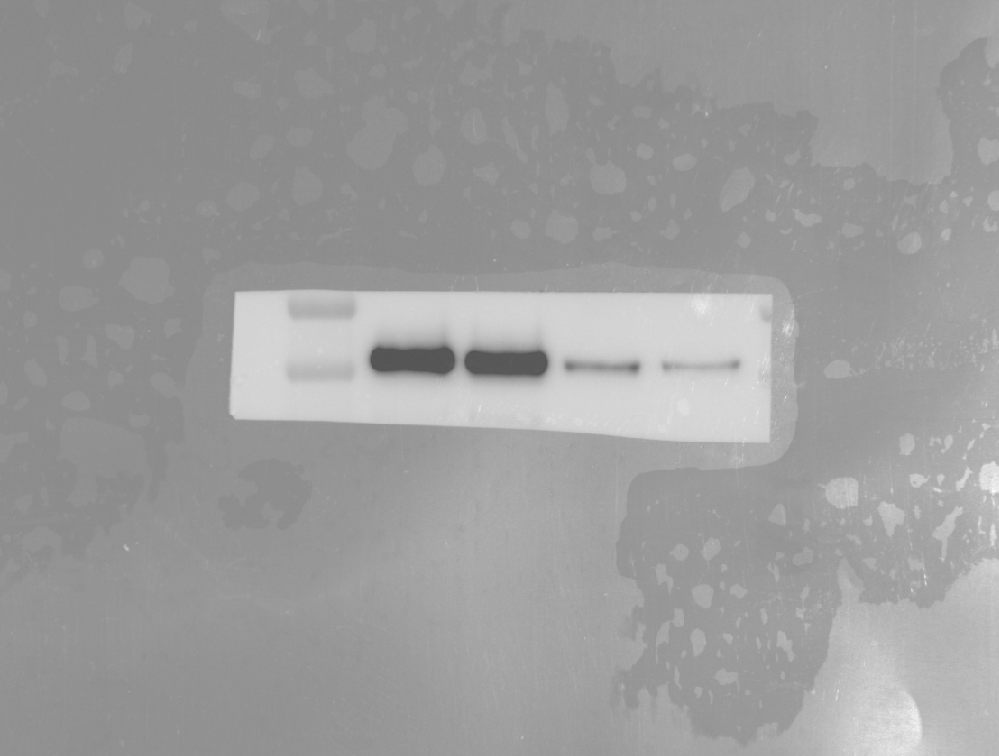

Supplement: Supplementary file 8 — Source Data Fig. 6 [file 44319_2023_27_MOESM8_ESM.zip › Figure 6/6C/western UBXN1.tif]

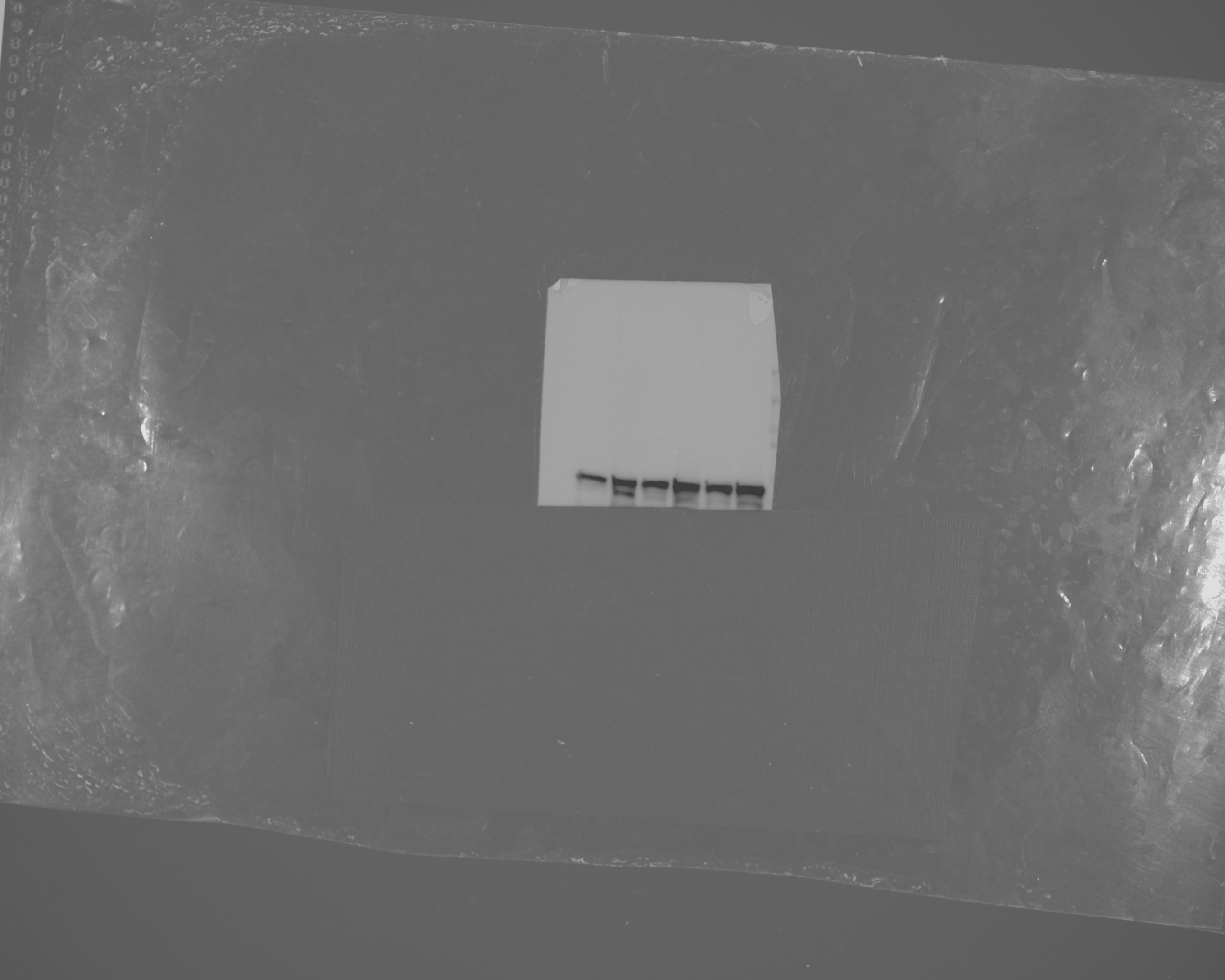

Supplement: Supplementary file 9 — Source Data Fig. 7 [file 44319_2023_27_MOESM9_ESM.zip › Figure 7/7C/Western Bactin.jpg]

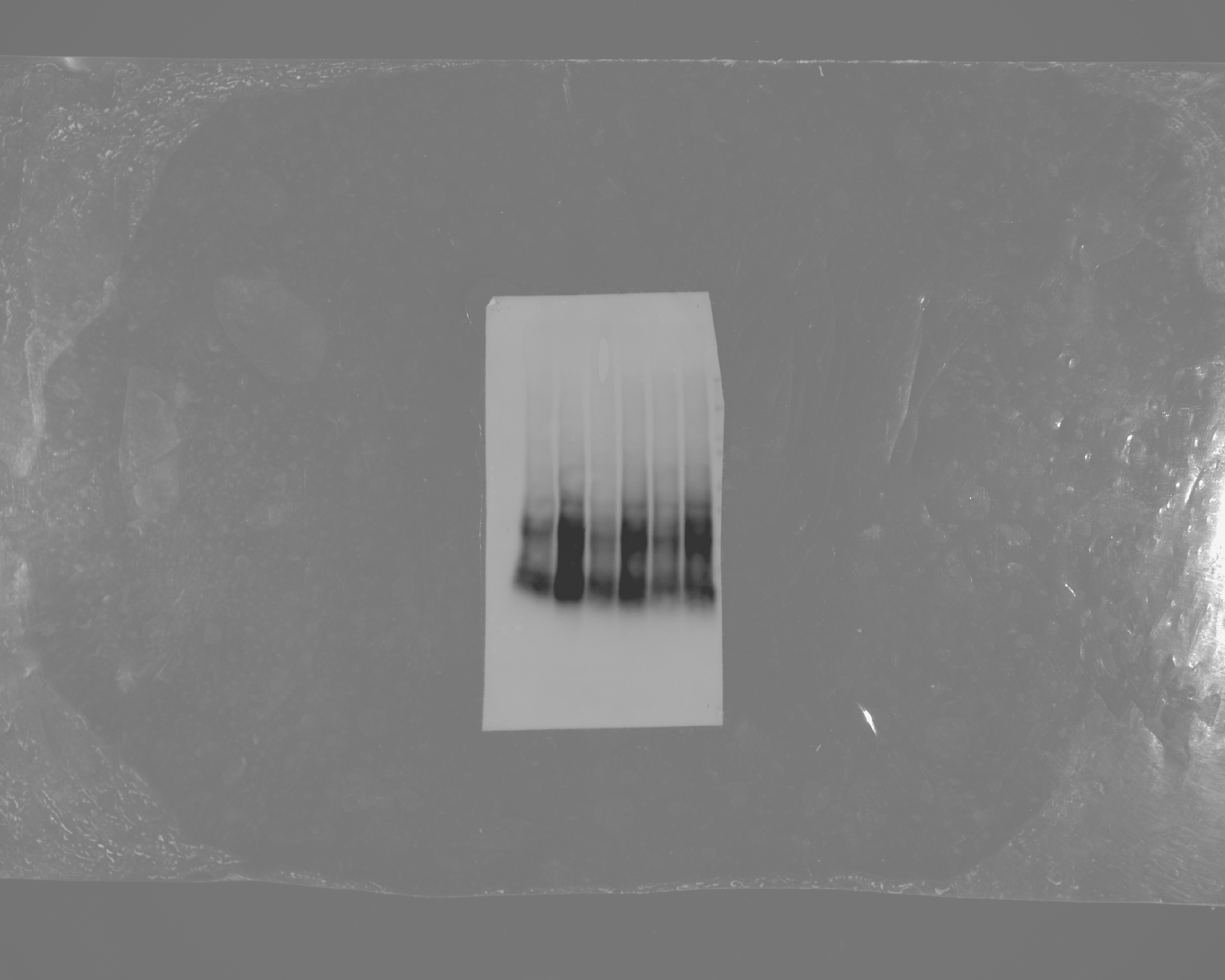

Supplement: Supplementary file 9 — Source Data Fig. 7 [file 44319_2023_27_MOESM9_ESM.zip › Figure 7/7C/Western puromycin.jpg]

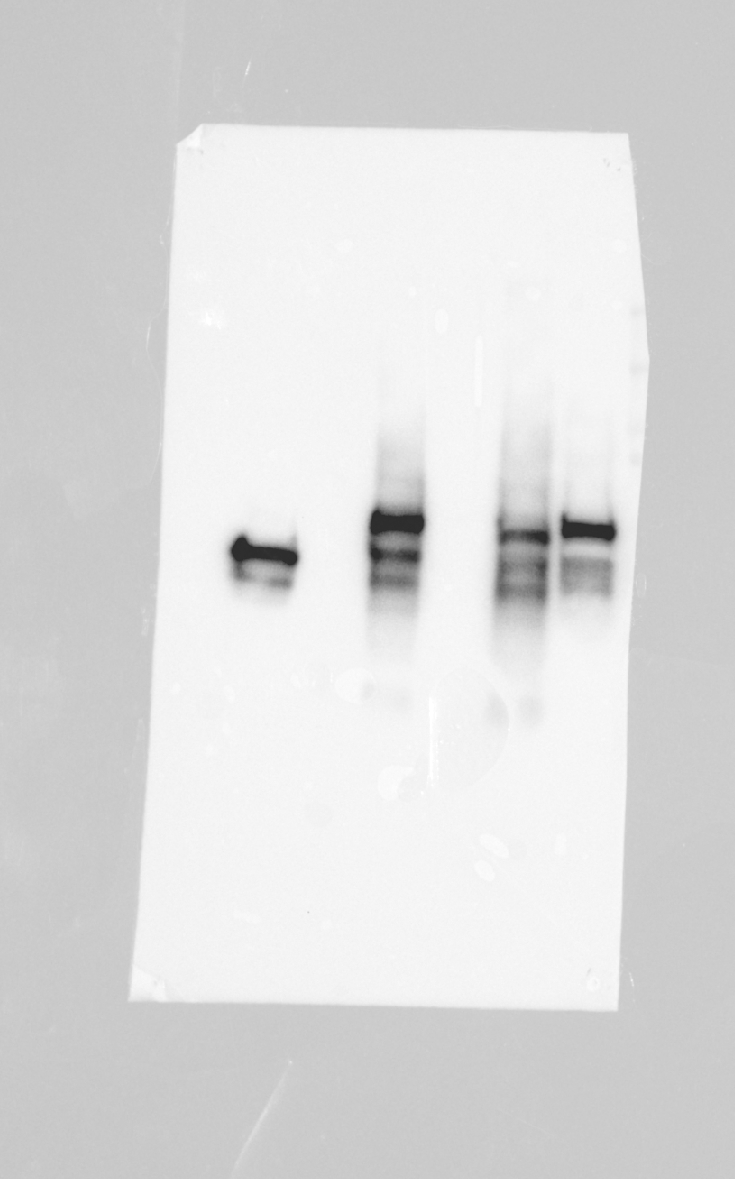

Supplement: Supplementary file 9 — Source Data Fig. 7 [file 44319_2023_27_MOESM9_ESM.zip › Figure 7/7C/Western UBXN1.tif]

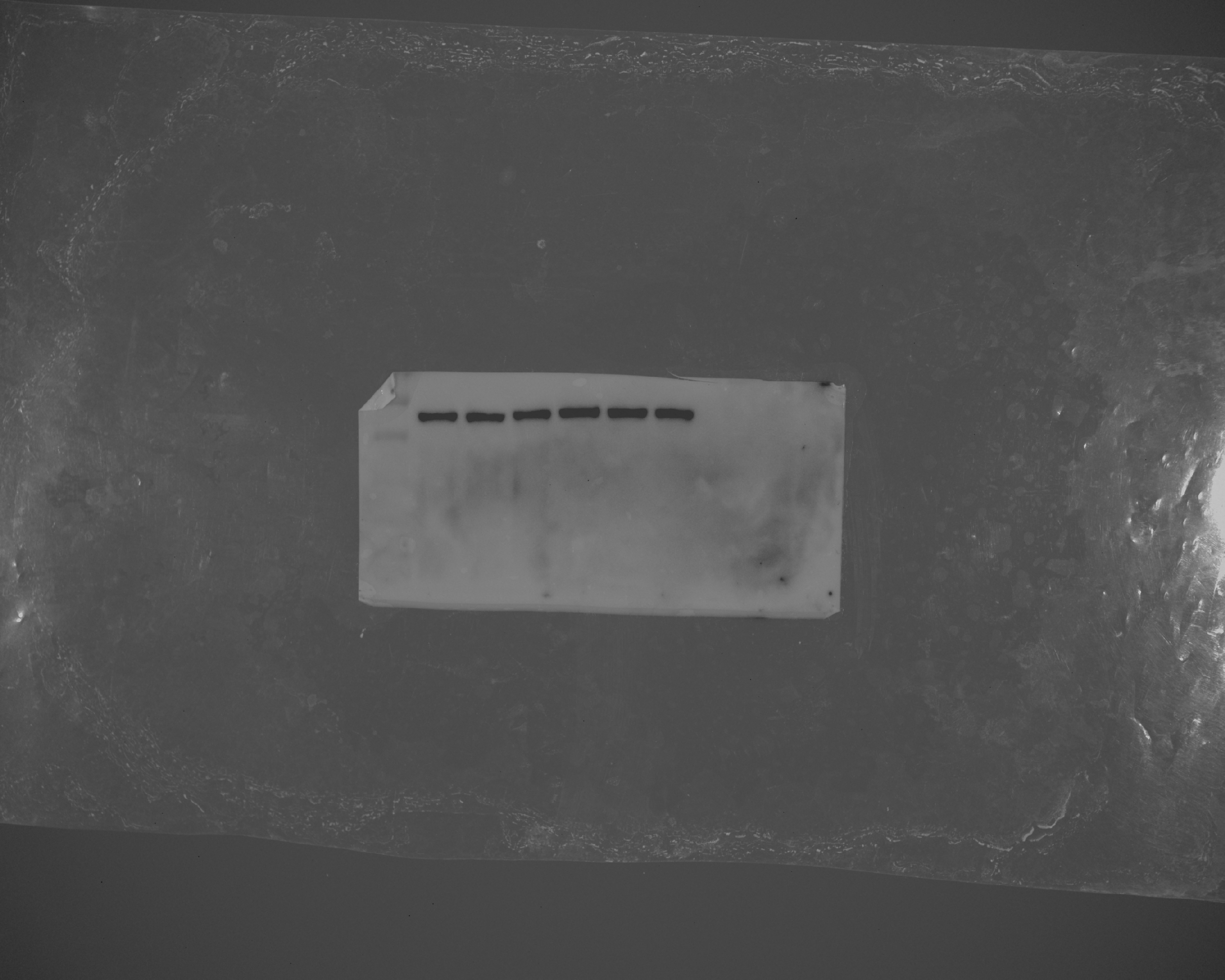

Supplement: Supplementary file 9 — Source Data Fig. 7 [file 44319_2023_27_MOESM9_ESM.zip › Figure 7/EV5C/Western Bactin.jpg]

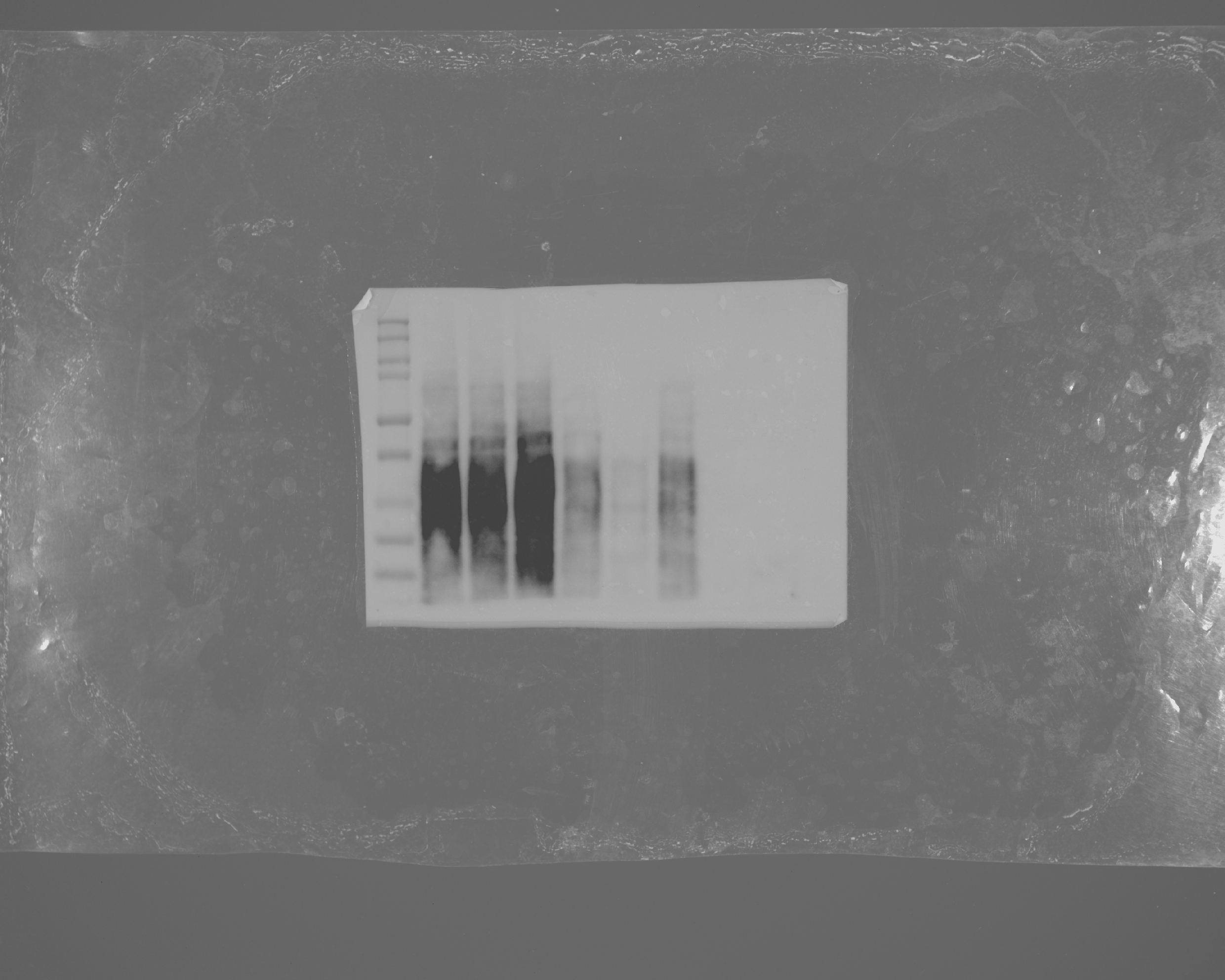

Supplement: Supplementary file 9 — Source Data Fig. 7 [file 44319_2023_27_MOESM9_ESM.zip › Figure 7/EV5C/Western puromycin.jpg]

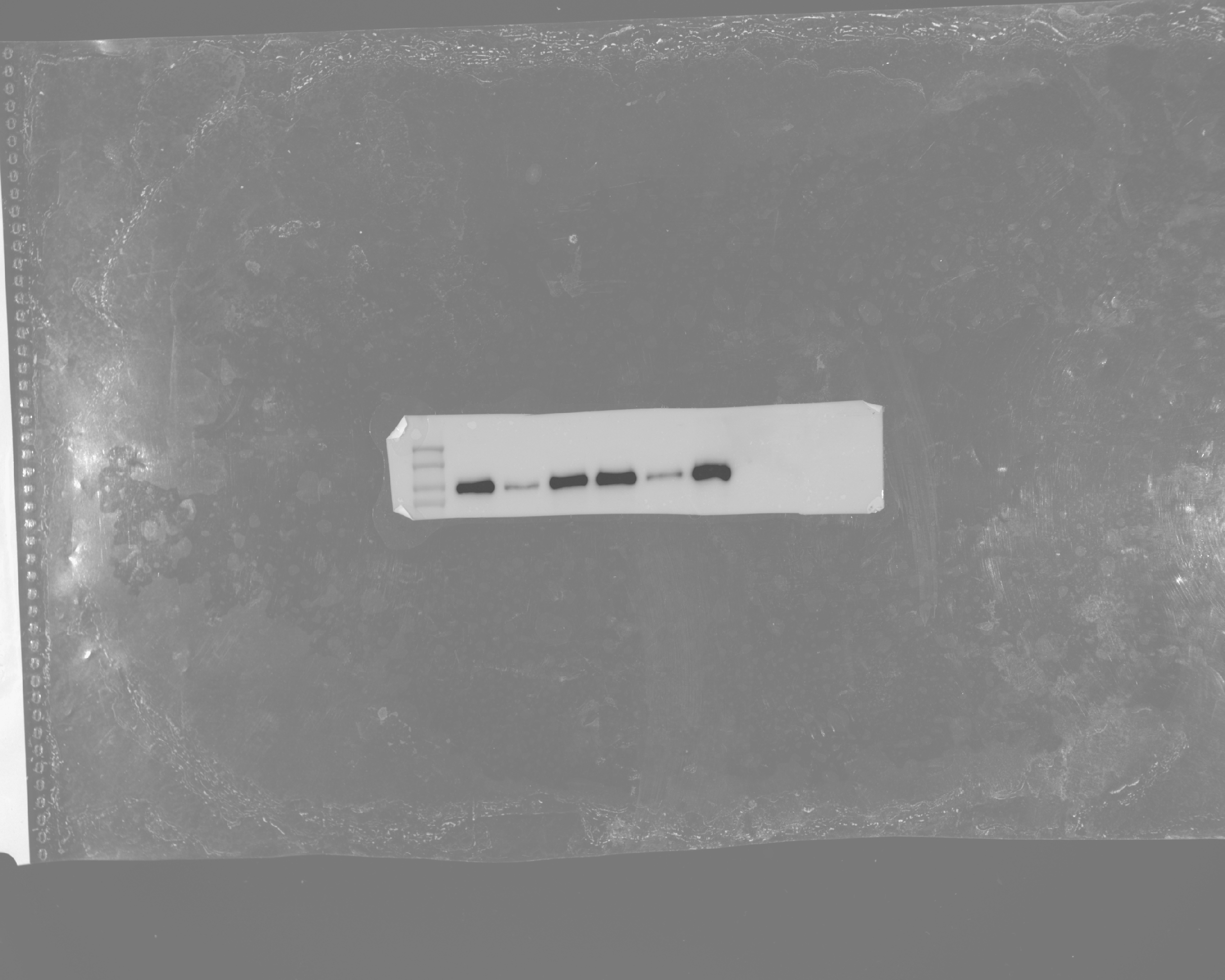

Supplement: Supplementary file 9 — Source Data Fig. 7 [file 44319_2023_27_MOESM9_ESM.zip › Figure 7/EV5C/Western p97 .jpg]

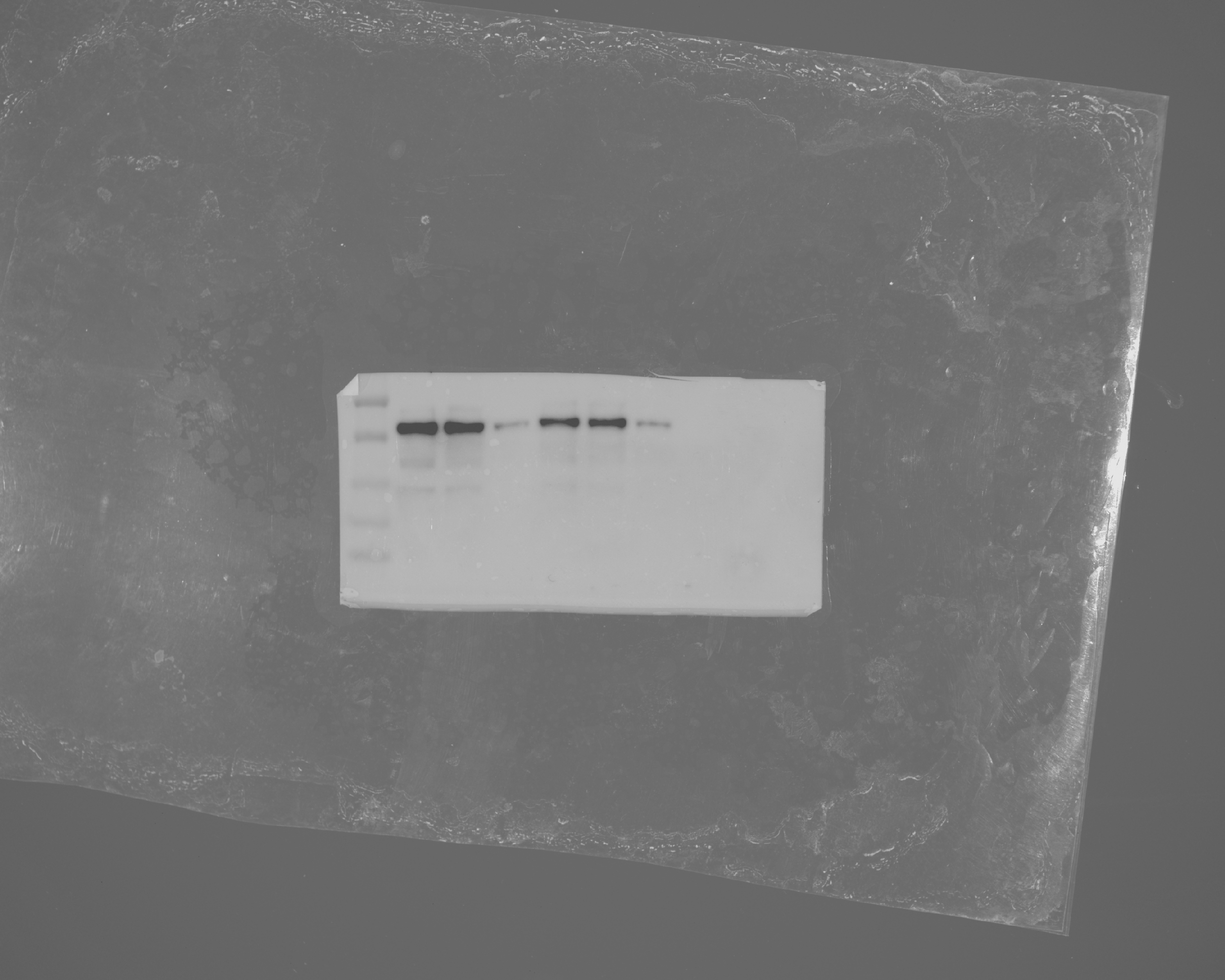

Supplement: Supplementary file 9 — Source Data Fig. 7 [file 44319_2023_27_MOESM9_ESM.zip › Figure 7/EV5C/Western UBXN1.jpg]

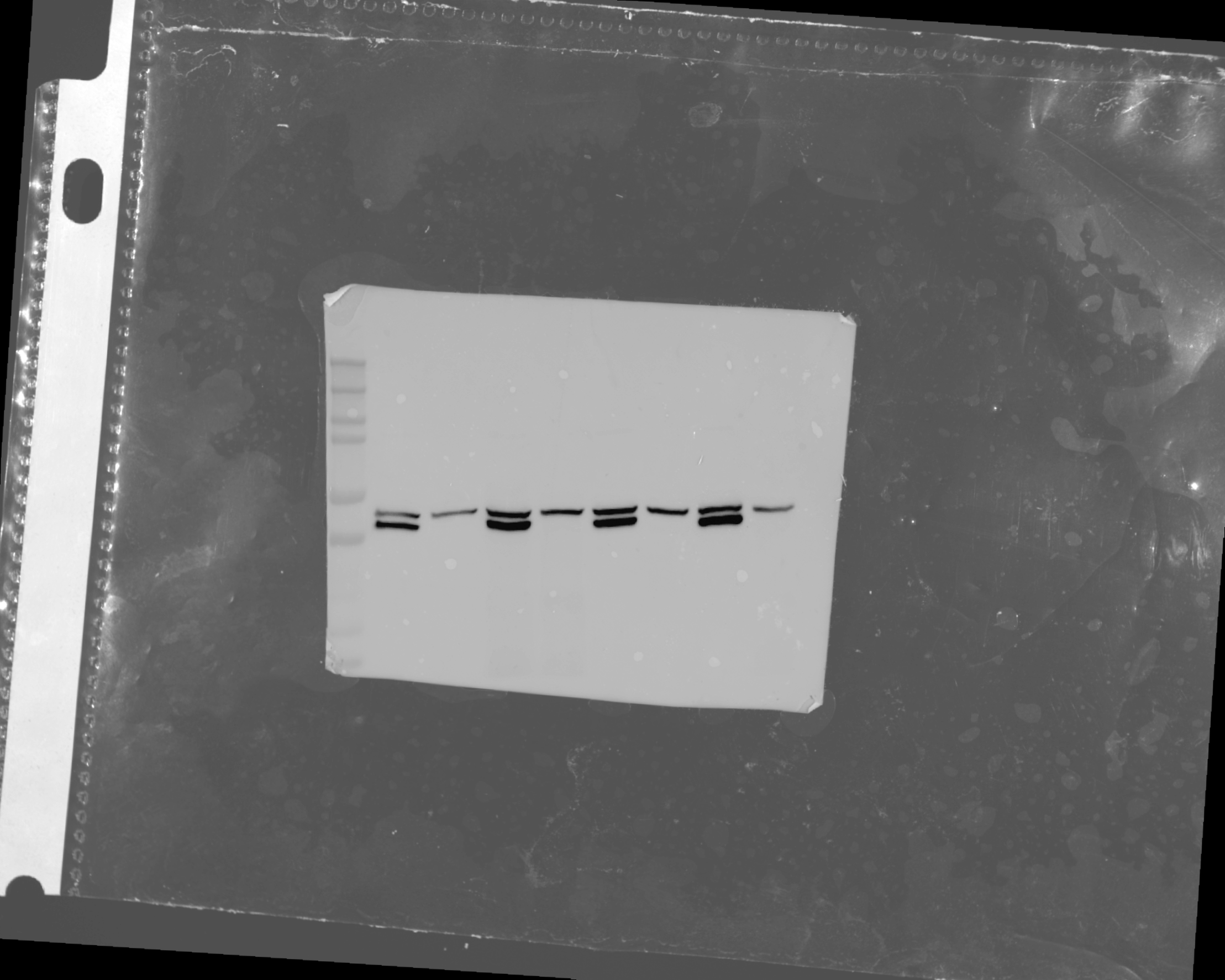

Supplement: Supplementary file 9 — Source Data Fig. 7 [file 44319_2023_27_MOESM9_ESM.zip › Figure 7/7A/Western Bactin and UBXN1.tif]

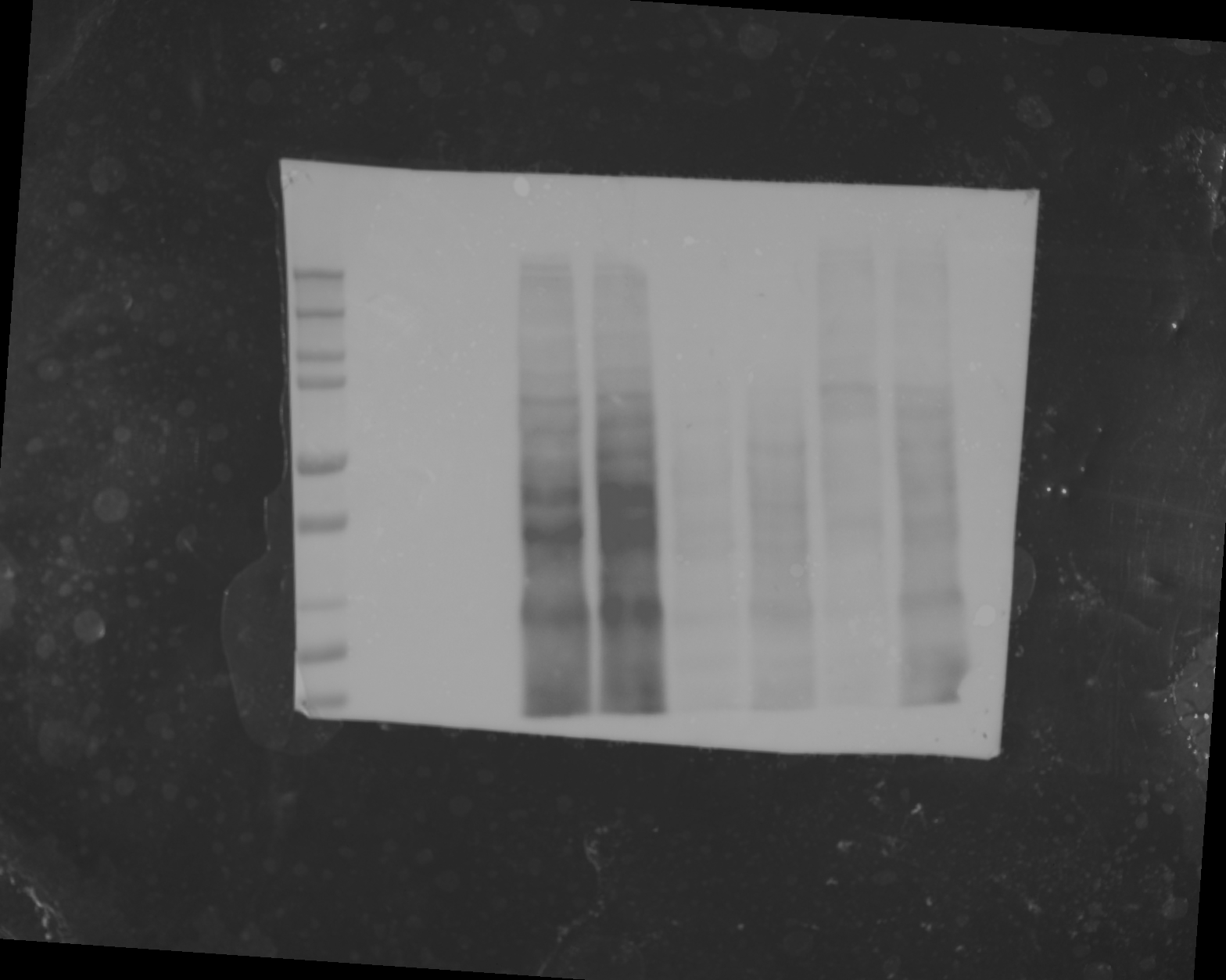

Supplement: Supplementary file 9 — Source Data Fig. 7 [file 44319_2023_27_MOESM9_ESM.zip › Figure 7/7A/Western puromycin.tif]
