# Supplementary material for: UBXN1 maintains ER proteostasis and represses UPR activation by modulating translation
Source: EMBO Rep. 2024 Jan 2;25(2):15. doi: 10.1038/s44319-023-00027-z (PMC10897191; doi:10.1038/s44319-023-00027-z)
Supplement: Supplementary file 12 — Expanded View Figures [file 44319_2023_27_MOESM12_ESM.pdf]

Expanded View Figures

Figure EV1. Acute BAG6 and UBXN1 depletion and pharmacological inhibition of p97 induces activation of the unfolded protein response.

(**A**) Immunoblot of BiP and ATF4 expression levels in HFT wildtype cells treated with 1 μM thapsigargin (Tg), 5 μM of the ATP-competitive p97 inhibitor CB-5083, or both for the indicated timepoints (0–6 hours). (**B**, **C**) Band intensity quantifications of BiP (**B**) and ATF4 (**C**) corresponding to Figure EV1A reporting the fold change compared to the untreated condition (n = three biologically independent samples). (**D**) Immunoblot of HFT wildtype cells depleted of BAG6 with siRNA for 48 hours before treatment with 1.5 mM DTT for the indicated timepoints (0–8 hours). (**E**) Band intensity quantifications of BiP and ATF4 corresponding to Figure EV1D at the 8-hour timepoint. (n = four biologically independent experiments). (**F**) Transcript levels of *xbp1s* and total *xbp1* in HEK-293T wildtype and UBXN1 KO cells quantified by quantitative real-time PCR. Cells were treated with 10 nM thapsigargin (Tg) for 4 hours as indicated. (n = three biologically independent samples). (**G**) Immunoblot of BiP and ATF4 expression in cells depleted of UBXN1 with siRNA. Cells were treated with 1.5 mM DTT for the indicated time points. (**H**) Band intensity quantifications of BiP and ATF4 from Figure EV1G at the 8-hour time point. (n = four biologically independent experiments). (**I**) Immunoblot of ATF6 activation in cells depleted of UBXN1 with siRNA. Cells were treated with 1.5 mM DTT and 1 μM Bortezomib (BTZ). (**J**) ATF6 activation was measured by band intensity quantification and calculation of the percentage of cleaved ATF6 to total ATF6. The ratio of the percentage of ATF6 activation in UBXN1 KO cells to wildtype is reported. (n = three biologically independent samples). Data information: Data are means ± SEM (*, **, ***, **** where $P < 0.05$, 0.01, 0.001, and 0.0001, respectively.) One-way ANOVA with Dunnetts multiple comparisons test (**B**, **C**). Unpaired two-tailed t test (**E**, **H**, **J**). One-way ANOVA with Tukey's multiple comparisons test (**F**).

© The Author(s)

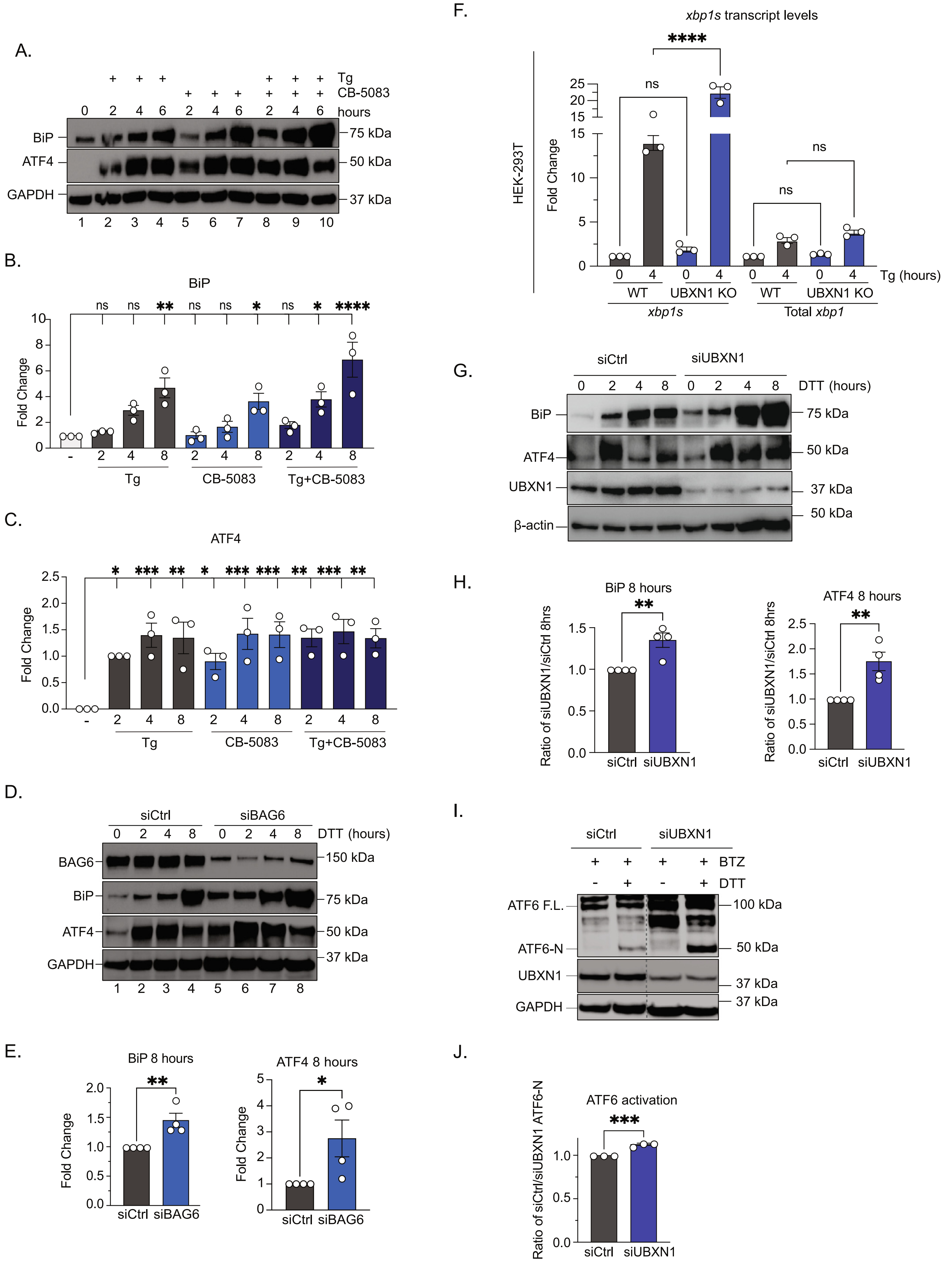
A.
Tg
CB-5083
hours
0 2 4 6 2 4 6 2 4 6
BiP
ATF4
GAPDH
75 kDa
50 kDa
37 kDa
1 2 3 4 5 6 7 8 9 10
B.
BiP
ns ns ** ns ns * ns * ****
Fold Change
- 2 4 8 2 4 8 2 4 8
Tg
CB-5083
Tg+CB-5083
C.
ATF4
* *** ** * *** *** ** *** **
Fold Change
- 2 4 8 2 4 8 2 4 8
Tg
CB-5083
Tg+CB-5083
D.
siCtrl
siBAG6
0 2 4 8 0 2 4 8 DTT (hours)
BAG6
BiP
ATF4
GAPDH
150 kDa
75 kDa
50 kDa
37 kDa
1 2 3 4 5 6 7 8
E.
BiP 8 hours
**
Fold Change
siCtrl siBAG6
ATF4 8 hours
*
Fold Change
siCtrl siBAG6
F.
xbp1s transcript levels
****
ns
ns
ns
HEK-293T
Fold Change
0 4 0 4 0 4 0 4 Tg (hours)
WT
UBXN1 KO
WT
UBXN1 KO
xbp1s
Total xbp1
G.
siCtrl
siUBXN1
0 2 4 8 0 2 4 8 DTT (hours)
BiP
ATF4
UBXN1
β-actin
75 kDa
50 kDa
37 kDa
50 kDa
H.
BiP 8 hours
**
Ratio of siUBXN1/siCtrl 8hrs
siCtrl siUBXN1
ATF4 8 hours
**
Ratio of siUBXN1/siCtrl 8hrs
siCtrl siUBXN1
I.
siCtrl
siUBXN1
+ + + + BTZ
- + - + DTT
ATF6 F.L.
ATF6-N
UBXN1
GAPDH
100 kDa
50 kDa
37 kDa
37 kDa
J.
ATF6 activation
***
Ratio of siCtrl/siUBXN1 ATF6-N
siCtrl siUBXN1

© The Author(s)

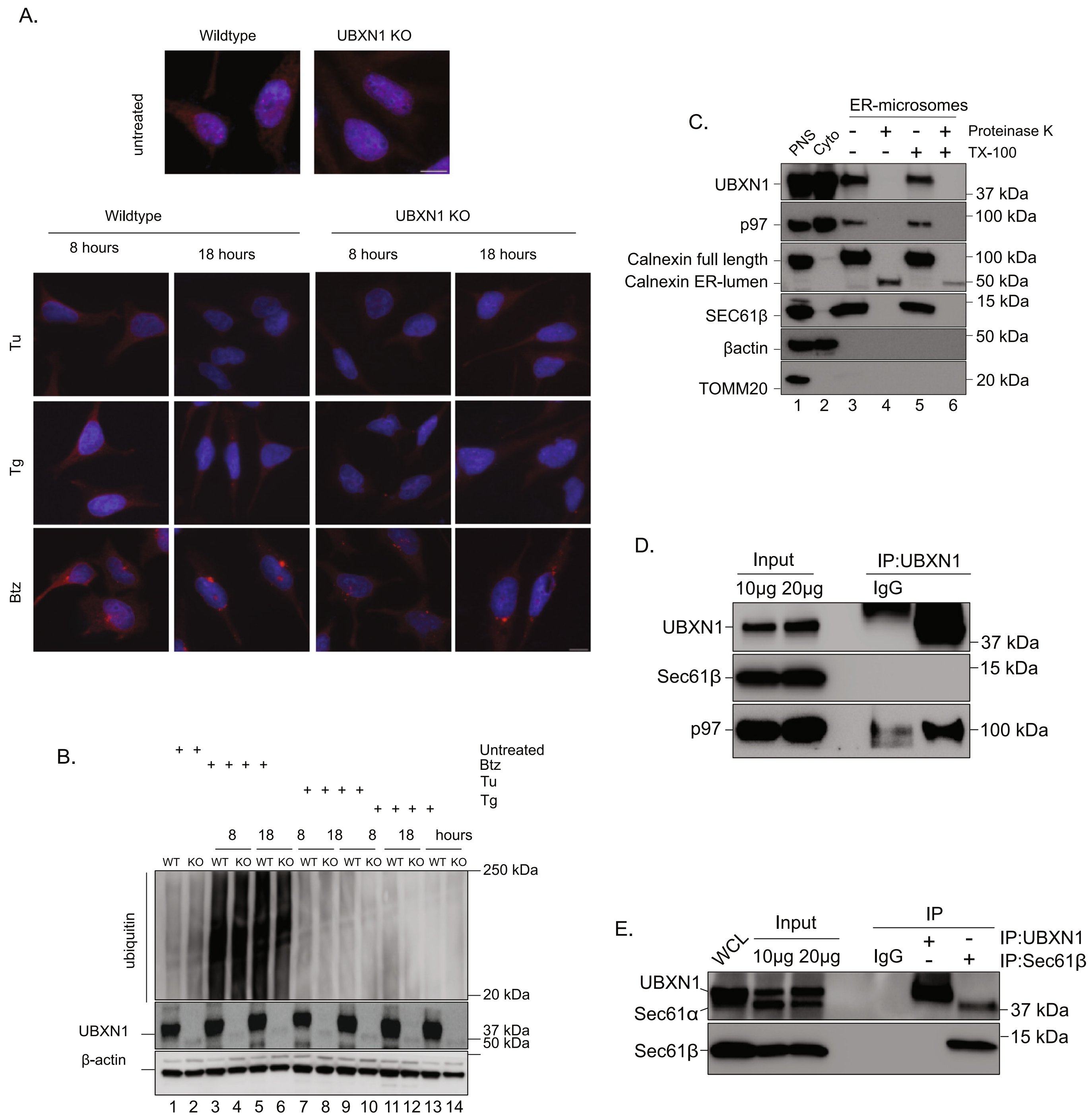

Figure EV2. UBXN1 localizes to ER-microsomes.

(**A**) Immunofluorescent staining of ubiquitin (FK2) and nuclei (Hoechst dye) in HFT wildtype and UBXN1 KO cells. Cells were treated with 1 μM of the proteasome inhibitor bortezomib (BTZ), Tunicamycin (Tu), or Thapsigargin (Tg) for 8 hours or 1 μM BTZ, 500 nM Tu, or 500 nM Tg for 18 hours. (Scale bar: 10 μm) (**B**) Immunoblot assessing total ubiquitin levels corresponding to Figure EV2A. (**C**) Subcellular fractions enriched in ER-derived microsomes were isolated from HEK-293T cells by biochemical fractionation. Protease protection assay was used to localize UBXN1 to the ER periphery by immunoblot. Calnexin and Sec61β are ER specific markers, β-actin is a cytosolic marker, and TOMM20 is a mitochondrial marker. (PNS: post-nuclear supernatant sample, cyto: cytosolic sample). (**D**) Immunoblot for the immunoprecipitation of endogenous UBXN1 from HEK-293T lysates. 10 and 20 μg of whole cell lysate was used for the input. Sec61β was used as a marker for the Sec61 translocon. (**E**) UBXN1 and Sec61β were separately immunoprecipitated from ER-microsomes isolated from HEK-293T cells. 10 and 20 μg of whole cell lysate was used for the input.

© The Author(s)

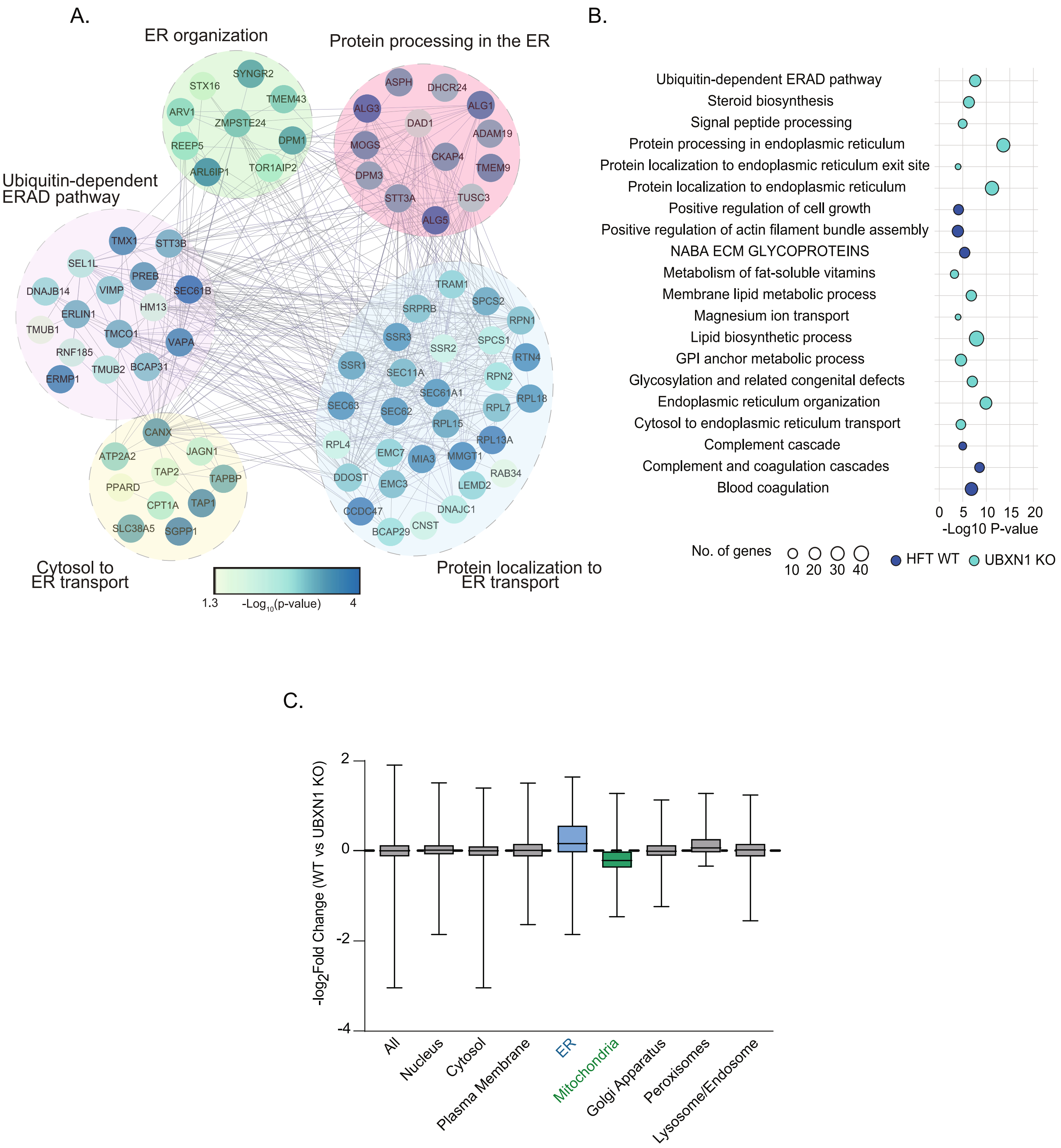

Figure EV3. Loss of UBXN1 leads to a perturbed ER proteome.

(**A**) Interaction network of the gene ontology clusters from the wildtype/UBXN1 KO quantitative proteomics study with a significant increase in abundance in UBXN1 KO cells. Proteins found in each node are labeled and the color of each circle corresponds to the $-\log_{10}$-transformed *P* value. (**B**) Bubble plot corresponding to significantly enriched gene ontology groups in the wildtype (navy) and UBXN1 KO (teal). The size of the circle corresponds to the number of genes identified in each term. (**C**) Proteins identified by proteomics categorized by organellar compartment. Proteins associated with the ER experience a significant increase in abundance in UBXN1 KO cells where mitochondrial proteins are significantly decreased n = two biological replicates. Box plots show median -log fold change (wildtype: knockout), upper quartile represents the 75th percentile and lower quartile represents 25th percentile. The whiskers represent minimum and maximum -log fold change (wildtype: knockout) for each category.

© The Author(s)

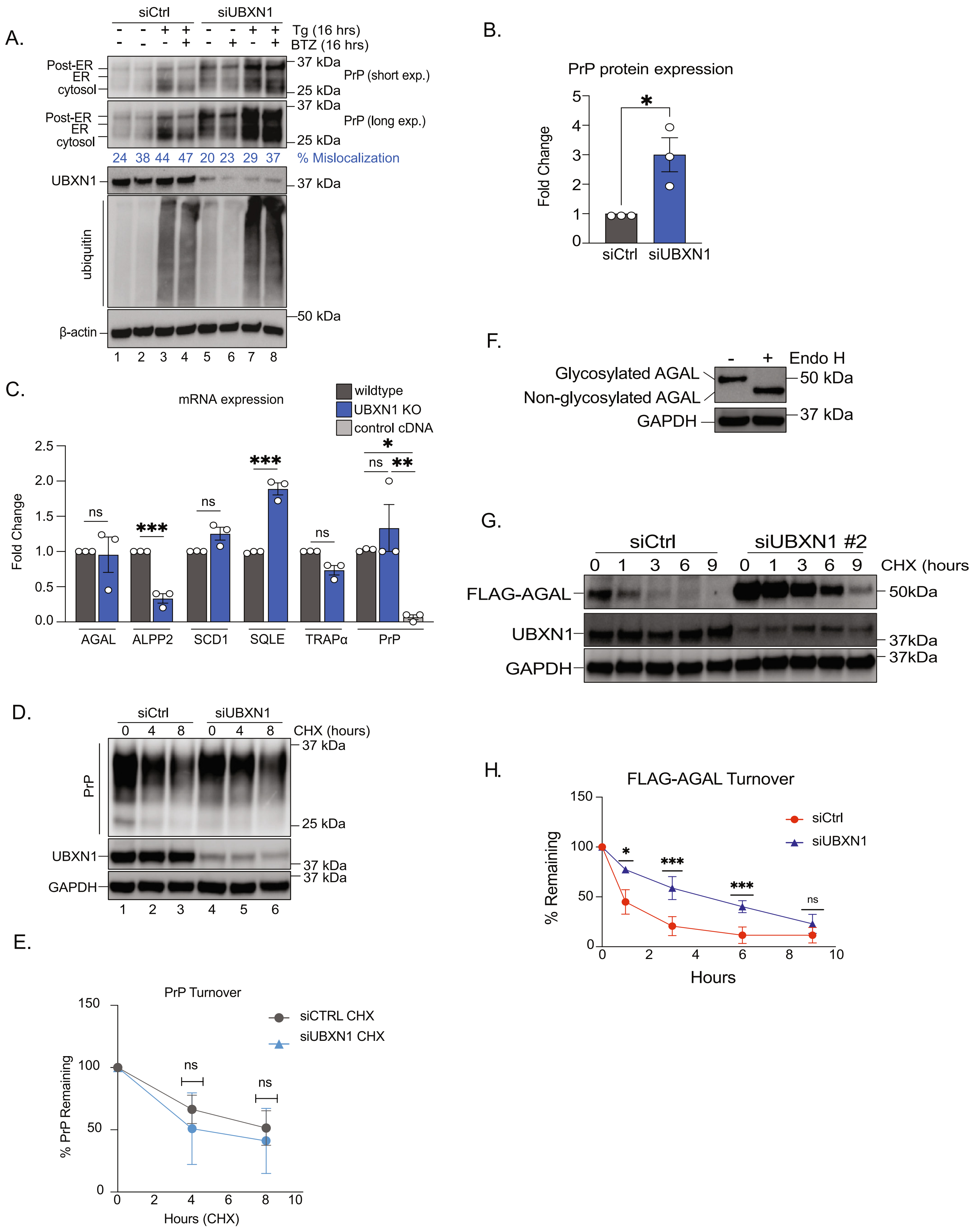
A.
siCtrl
siUBXN1
Tg (16 hrs)
BTZ (16 hrs)
Post-ER
ER
cytosol
37 kDa
PrP (short exp.)
25 kDa
PrP (long exp.)
24 38 44 47 20 23 29 37
% Mislocalization
UBXN1
ubiquitin
50 kDa
β-actin
1 2 3 4 5 6 7 8
B.
PrP protein expression
Fold Change
siCtrl siUBXN1
C.
mRNA expression
wildtype
UBXN1 KO
control cDNA
Fold Change
AGAL ALPP2 SCD1 SQLE TRAPα PrP
D.
siCtrl
siUBXN1
CHX (hours)
PrP
UBXN1
GAPDH
E.
PrP Turnover
siCTRL CHX
siUBXN1 CHX
% PrP Remaining
Hours (CHX)
F.
Endo H
Glycosylated AGAL
Non-glycosylated AGAL
GAPDH
50 kDa
37 kDa
G.
siCtrl
siUBXN1 #2
CHX (hours
FLAG-AGAL
UBXN1
GAPDH
50kDa
37kDa
H.
FLAG-AGAL Turnover
siCtrl
siUBXN1
% Remaining
Hours

Figure EV4. Depletion of UBXN1 increases the expression of ER-destined proteins.

(**A**) Immunoblot of HFT wildtype cells expressing HA-tagged prion protein (PrP). PrP was expressed in HeLa cells for before siRNA mediated depletion of UBXN1. Cells were treated with 500 nM thapsigargin, 1 μM bortezomib (BTZ), or both for 16 hours. The percent mislocalization was calculated from the ratio of the mislocalized (cytosolic) form to all forms of PrP for that sample. (**B**) Total PrP protein expression was calculated by the sum of all forms of PrP for that sample. The untreated condition was quantified (lanes 1 and 5) (n = three biologically independent experiments). (**C**) Transcript levels of several ER-destined proteins from cells depleted of UBXN1 by siRNA quantified by real-time PCR. Control is cDNA generated from cells not expressing HA-PrP. (n = three biologically independent experiments). (**D**) Immunoblot examining the turnover of HA-PrP in siRNA control and UBXN1 depleted cells. Cells were treated with 10 μg/mL cycloheximide (CHX) for the indicated timepoints (0–8 hours). (**E**) Quantification of PrP turnover from Figure EV4D. (**F**) Immunoblot of a stable HFT cell line expressing doxycycline inducible FLAG-tagged AGAL. Glycans were digested with EndoH for 4 hours to generate the non-glycosylated form. (**G**) Turnover of FLAG-AGAL in HEK293T cells depleted of UBXN1 and treated with cycloheximide to monitor half life. (**H**) Quantification of EV4G. For turnover studies, the zero-hour time point was set to 100% for each siRNA and the % of PrP or AGAL remaining for each time point was quantified as a ratio from the zero-hour timepoint. (n = three biologically independent experiments). Data information: Data are means ± SEM (*, **, and *** where $P < 0.05$, 0.01, and 0.001 respectively.) Unpaired two-tailed t test (**B**, **C**, **E**, **H**).

 © The Author(s)

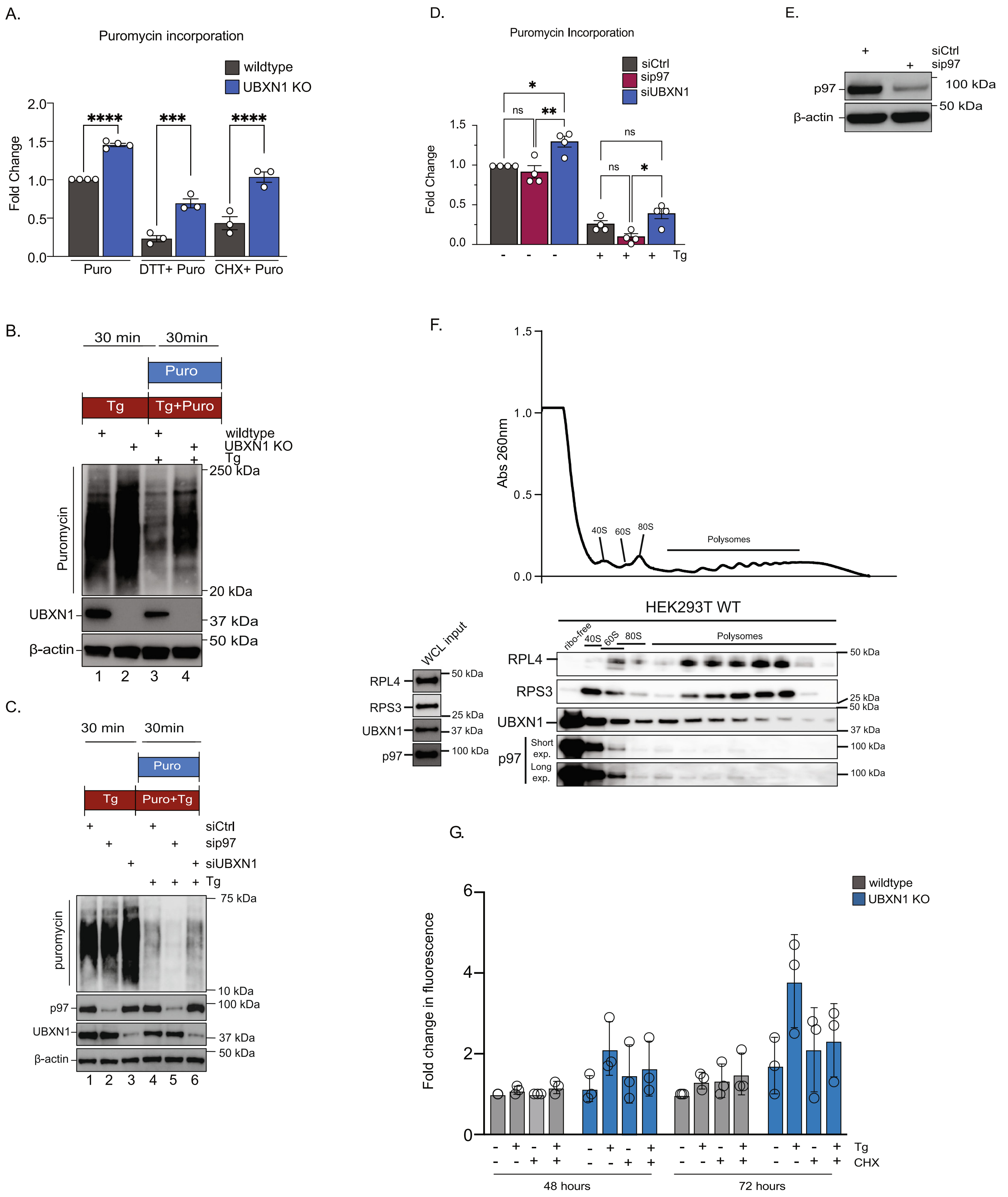
A.
Puromycin incorporation
wildtype
UBXN1 KO
Fold Change
****
***
****
Puro
DTT+ Puro
CHX+ Puro
B.
30 min
30min
Puro
Tg
Tg+Puro
wildtype
UBXN1 KO
Tg
250 kDa
20 kDa
Puromycin
UBXN1
37 kDa
β-actin
50 kDa
1 2 3 4
C.
30 min
30min
Puro
Tg
Puro+Tg
siCtrl
sip97
siUBXN1
Tg
75 kDa
10 kDa
puromycin
p97
100 kDa
UBXN1
37 kDa
β-actin
50 kDa
1 2 3 4 5 6
D.
Puromycin Incorporation
siCtrl
sip97
siUBXN1
Fold Change
ns
Tg
E.
siCtrl
sip97
p97
100 kDa
β-actin
50 kDa
F.
Abs 260nm
40S
60S
80S
Polysomes
HEK293T WT
WCL input
ribo-free
RPL4
RPS3
UBXN1
p97
Short exp.
Long exp.
50 kDa
25 kDa
37 kDa
100 kDa
G.
wildtype
UBXN1 KO
Fold change in fluorescence
Tg
CHX
48 hours
72 hours

Figure EV5. UBXN1 suppresses protein synthesis.

(**A**) Densitometry quantifications of the entire lanes corresponding to Fig. 7A ($n \geq$ three biologically independent experiments). (**B**) Immunoblot of HFT wildtype and UBXN1 KO cells pulsed with 1 μM puromycin for 30 min after pre-treatment with 1.5 μM thapsigargin for 30 minutes where indicated. (**C**) Immunoblot of 1 μM puromycin incorporation into control cells, or cells depleted of p97 or UBXN1 with siRNA for 48 hours. Cells were pre-treated with 1 μM thapsigargin for 30 minutes where indicated. (**D**) Quantification of the whole lane corresponding to each lane in the immunoblot in Figure EV5C (n = four biologically independent samples). (**E**) Immunoblot validating the siRNA knockdown corresponding to Fig. 7F. (**F**) Immunoblot of ribosome and polysome fractions collected with corresponding UV traces showing 40S, 60S, 80S and polysome fractions. RPL4 is a marker for the 60S subunit and RPS3 is a marker for the 40S subunit. Whole cell lysates (WCL) are shown for input. (**G**) UBXN1 KO cells have similar viability to wildtype cells under resting conditions. Data shown in Main Fig. 8B is shown here without normalization to untreated in each genotype. We observe no differences in viability between wildtype and UBXN1 KO cells in untreated conditions allowing for this normalization. Fold change of the fluorescence measured by fluorescence-based cytotoxicity assay. Cells were treated with 1.5 μM thapsigargin in combination with 10 μg/ml cycloheximide where indicated. Values were normalized to wildtype untreated. (n = three biologically independent samples). Data information: Data are means ± SEM (*, **, *** and **** where $P < 0.05$, 0.01, 0.001 and 0.0001, respectively.) Unpaired two-tailed t test (**A**). One-way ANOVA with Tukey's multiple comparisons test (**D**, **G**).

 © The Author(s)
